# Reaction Mechanism and Substrate Specificity of *Iso*-orotate Decarboxylase: A Combined Theoretical and Experimental Study

**DOI:** 10.3389/fchem.2018.00608

**Published:** 2018-12-19

**Authors:** Xiang Sheng, Katharina Plasch, Stefan E. Payer, Claudia Ertl, Gerhard Hofer, Walter Keller, Simone Braeuer, Walter Goessler, Silvia M. Glueck, Fahmi Himo, Kurt Faber

**Affiliations:** ^1^Department of Organic Chemistry, Arrhenius Laboratory, Stockholm University, Stockholm, Sweden; ^2^Institute of Chemistry, Organic & Bioorganic Chemistry, University of Graz, Graz, Austria; ^3^Institute of Molecular Biosciences, University of Graz, Graz, Austria; ^4^Institute of Chemistry, Analytical Chemistry, University of Graz, Graz, Austria; ^5^Austrian Centre of Industrial Biotechnology (ACIB GmbH), Graz, Austria

**Keywords:** computational chemistry, biocatalysis, *iso*-orotate decarboxylase, reaction mechanism, substrate specificity, metal identity

## Abstract

The C-C bond cleavage catalyzed by metal-dependent *iso*-orotate decarboxylase (IDCase) from the thymidine salvage pathway is of interest for the elucidation of a (hypothetical) DNA demethylation pathway. IDCase appears also as a promising candidate for the synthetic regioselective carboxylation of *N*-heteroaromatics. Herein, we report a joint experimental-theoretical study to gain insights into the metal identity, reaction mechanism, and substrate specificity of IDCase. In contrast to previous assumptions, the enzyme is demonstrated by ICPMS/MS measurements to contain a catalytically relevant Mn^2+^ rather than Zn^2+^. Quantum chemical calculations revealed that decarboxylation of the natural substrate (5-carboxyuracil) proceeds *via* a (reverse) electrophilic aromatic substitution with formation of CO_2_. The occurrence of previously proposed tetrahedral carboxylate intermediates with concomitant formation of ${\text{HCO}}_{3}^{-}$ could be ruled out on the basis of prohibitively high energy barriers. In contrast to related *o*-benzoic acid decarboxylases, such as γ-resorcylate decarboxylase and 5-carboxyvanillate decarboxylase, which exhibit a relaxed substrate tolerance for phenolic acids, IDCase shows high substrate fidelity. Structural and energy comparisons suggest that this is caused by a unique hydrogen bonding of the heterocyclic natural substrate (5-carboxyuracil) to the surrounding residues. Analysis of calculated energies also shows that the reverse carboxylation of uracil is impeded by a strongly disfavored uphill reaction.

## Introduction

**Scheme 1:**
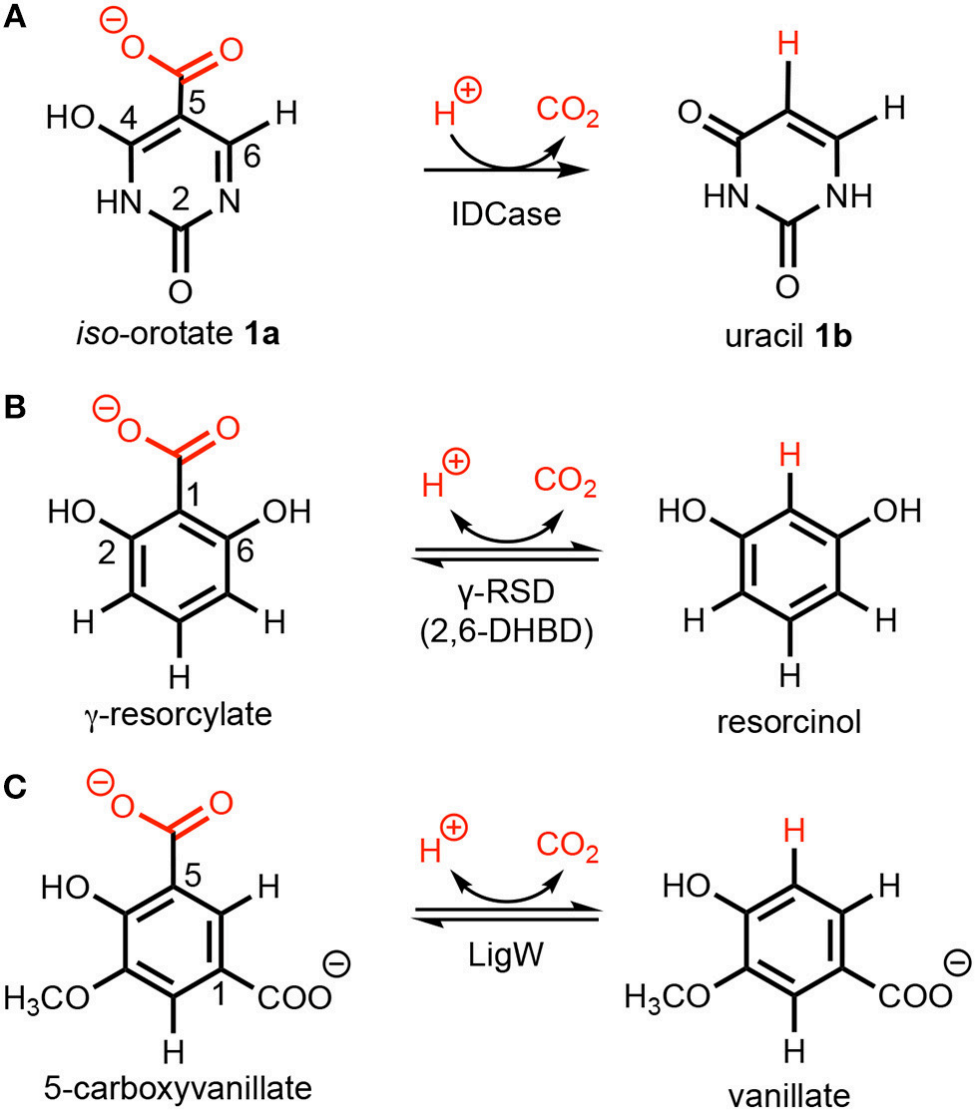
Decarboxylation of (hetero)aromatics catalyzed by **(A)** *iso*-orotate decarboxylase (IDCase), **(B)** γ-resorcylate decarboxylase (γ-RSD), and **(C)** 5-carboxyvanillate decarboxylase (LigW).

*Iso*-orotate decarboxylase (IDCase), an enzyme involved in the thymidine salvage pathway, catalyzes the non-oxidative decarboxylation of *iso*-orotate (5-carboxyuracil; 5caU; **1a**) to uracil (U; **1b**) (Smiley et al., 2005; Leal et al., 2007) (Scheme 1A). The latter can be directly converted to uridine monophosphate (UMP) by uracil phosphoribosyltransferase (UPRTase) in most organisms (Smiley et al., 2005). In the genomes of *Neurospora crassa* and *Aspergillus nidulans*, the IDCase gene is downstream from a gene encoding a dioxygenase termed thymine-7-hydroxylase, which oxidizes the methyl group of 5-methyluracil (thymin) to a carboxylate, thereby providing the substrate for IDCase (Smiley et al., 2005) and completing the pathway.

The enzyme is inactive on the regio-isomer orotic acid (6-carboxyuracil) and the reverse carboxylation of uracil (Palmatier et al., 1970), but decarboxylates 5-carboxy-2-thiouracil (Smiley et al., 1999) and 5-carboxycytosine (5caC) (Xu et al., 2013). The conversion of 5caC to cytosine (C) via decarboxylation (Schiesser et al., 2012) is suggested as the C-C cleaving step in a hypothetical DNA demethylation pathway mediated by Tet proteins (He et al., 2011; Ito et al., 2011), although such a “DNA decarboxylase” has not yet been identified. Hence, detailed knowledge of the structure and reaction mechanism of IDCase would provide valuable information on the identification of this putative DNA decarboxylase.

A number of crystal structures of IDCase from *Cordyceps militaris* and *Metarhizium anisopliae* have been obtained (Xu et al., 2013), and structural and sequence analysis showed that IDCase belongs to the amidohydrolase superfamily (Xu et al., 2013). A metal ion, identified as zinc, was observed to be coordinated by one aspartate and three histidine residues and the substrate is supposed to be directly bound to the metal by both the hydroxyl and the carboxylate group (Xu et al., 2013). The *K*_m_ and *k*_cat_ values were determined to be 22.4 ± 1.3 μM and 4.17 ± 0.09 min^−1^ for the IDCase from *C. militaris*, and 18.6 ± 1.9 μM and 2.02 ± 0.08 min^−1^ for IDCase from *M. anisopliae*, respectively (Xu et al., 2013). As a member of cog2159 of the amidohydrolase superfamily (Seibert and Raushel, 2005), IDCase shows structural and substrate similarities with other enzymes from the same family (Scheme 1), such as γ-resorcylate decarboxylase (also called 2,6-dihydroxybenzoic acid decarboxylase, 2,6-DHBD) from *Rhizobium* sp. (γ-RSD_Rs) (Wuensch et al., 2012; Sheng et al., 2018) and 5-carboxyvanillate decarboxylases from *Sphingomonas paucimobilis* (LigW_Sp) and from *Novosphingobium aromaticivorans* (LigW_Na) (Peng et al., 2002, 2005; Vladimirova et al., 2016; Sheng et al., 2017).

Interestingly, from a synthetic standpoint, *ortho*-benzoic acid decarboxylases (*o*-BDCs), such as 2,6-DHBD, have been shown to possess a remarkably broad substrate range for the reverse regioselective carboxylation of phenolic compounds to produce aromatic carboxylic acids used as pharmaceuticals as well as building blocks for organic synthesis (Ishii et al., 2004; Yoshida et al., 2004; Matsui et al., 2006; Iwasaki et al., 2007; Ienaga et al., 2013; Wuensch et al., 2014). This constitutes a biological alternative to the (chemical) Kolbe–Schmitt carboxylation process, which requires high pressure and temperature (Lindsey and Jeskey, 1957). Aiming to extend this method to the regioselective carboxylation of *N*-heteroaromatics, IDCase appeared as promising candidate.

In the present study, the metal dependence of IDCase is unambiguously established by means of ICPMS/MS experiments, followed by a detailed quantum chemical investigation to elucidate its reaction mechanism. Aiming at using IDCase in the reverse carboxylation reaction, the natural substrate and a range of synthetic analogs (such as structurally related pyrimidine and phenol derivatives) were examined. Sequence alignment of IDCase with related metal-dependent decarboxylases is performed and their active sites are compared. Finally, an energy analysis of different substrate binding modes is conducted.

## Results and Discussion

### Metal-Dependence

All structures of IDCase showed a metal at the active site, which was assumed to be Zn^2+^ based on fluorescence spectroscopy (PDB 4LAK and 4HJW) (Xu et al., 2013). In the substrate (*iso*-orotate)-bound IDCase structure (Asp323Asn mutant, PDB 4LAM), the metal is coordinated to C4-hydroxyl group of the pyrimidine ring and one oxygen of the carboxylate group and H-bonded to four amino acid residues (His12, His14, His195, and Asp323Asn).

**Figure 1:**
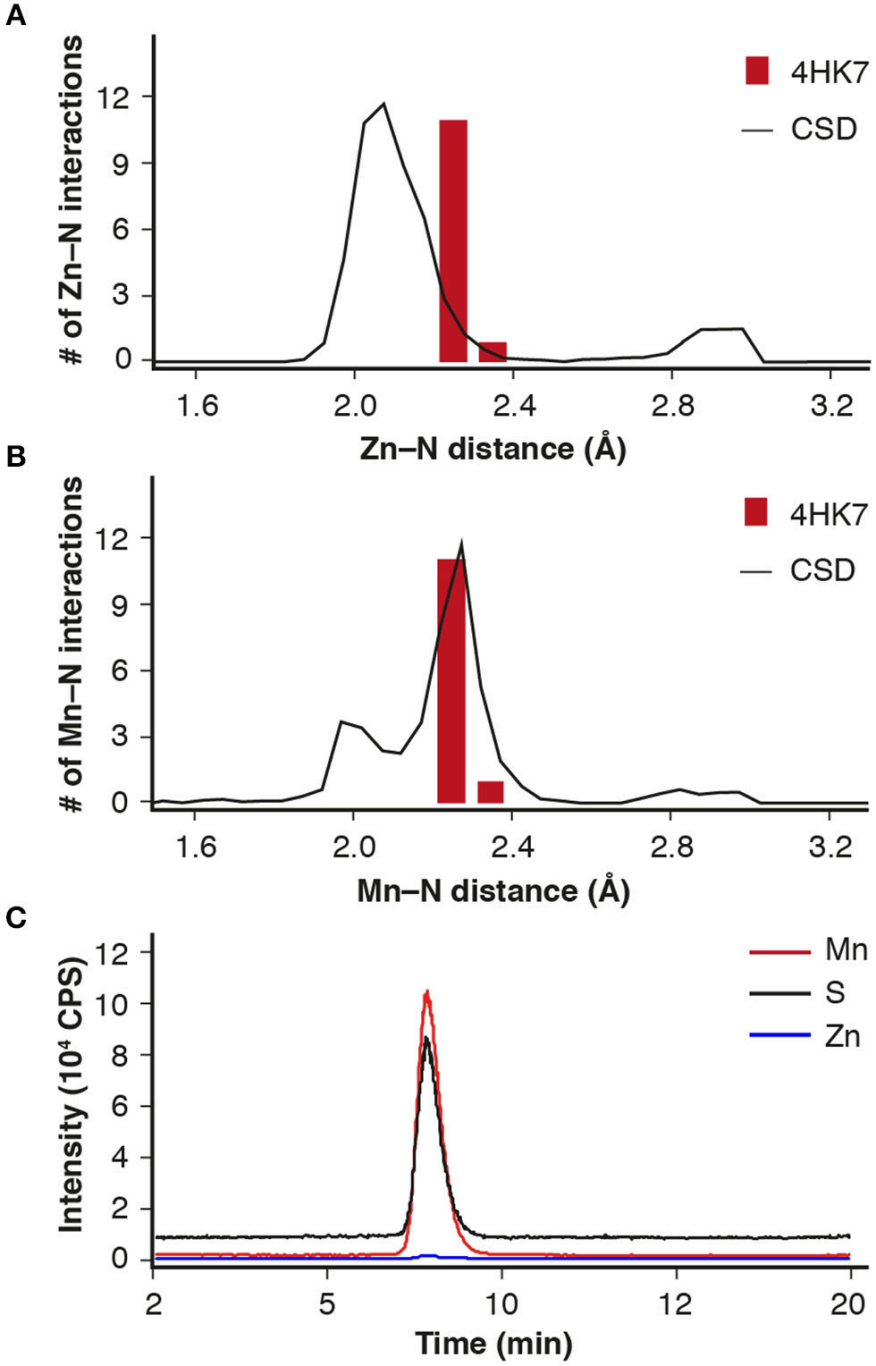
Metal-ligand distance (red bars) for IDCase (PDB 4HK7) with **(A)** Zn^2+^ and **(B)** Mn^2+^ in the active site compared with database likelihoods (CSD); **(C)** ICPMS/MS analysis of metal ions (Mn^2+^ and Zn^2+^) in IDCase (sulfur determination for quantitative analysis of protein).

Analysis of the metal-ligand distance of the (putative) Zn^2+^ in the crystal structure of IDCase (PDB 4HK7) (Zheng et al., 2014, 2017) showed that the metal–nitrogen bonds are too long for Zn^2+^, but fit nicely to a larger metal, such as Mn^2+^, which is frequently found in mechanistically related *o*-BDCs and LigWs (Sheng et al., 2017, 2018) (Figures 1A,B). In order to solve this discrepancy, ICPMS/MS measurements coupled to size exclusion chromatography were performed, which unambiguously proved the presence of Mn^2+^ (Figure 1C, red line) instead of Zn^2+^ (blue line) in the *Escherichia coli* expressed enzyme from *C. militaris*.

### Reaction Mechanism

**Figure 2:**
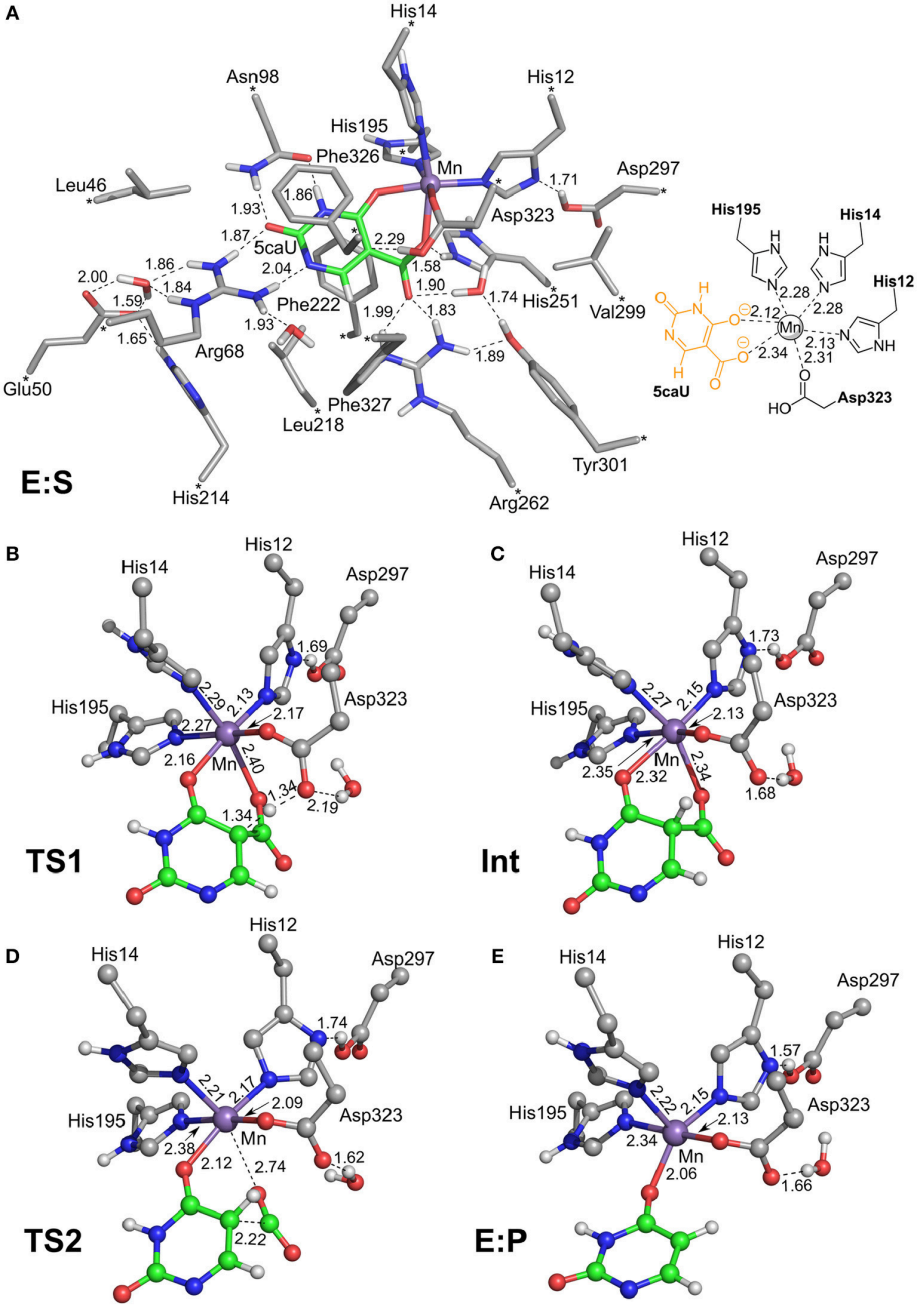
Optimized structures of intermediates and transition states along the reaction pathway proposed for IDCase. **(A)** The enzyme-substrate complex **E:S**, **(B)** the TS for the protonation step, **(C)** the intermediate after the protonation step, **(D)** the TS for the C-C bond cleavage step, and **(E)** the enzyme-product complex **E:P**. For clarity, only polar hydrogen atoms and hydrogens on the substrate are shown, and the full model is only shown for **E:S**. The atoms fixed during geometry optimization are marked with asterisks in **E:S** and selected distances are given in Å.

To investigate the reaction mechanism of IDCase, quantum chemical calculations were performed on the basis of the crystal structure of the Asp323Asn mutant from *C. militaris* in complex with the substrate (PDB 4LAM) (Xu et al., 2013). A large active site model consisting of 310 atoms was designed by modifying the mutated Asn323 back to the native Asp residue (Figure 2). Since the metal was identified above as in fact being Mn^2+^, the zinc ion previously proposed in the crystal structure is replaced by manganese. The computational methods and the details of the active site model are given in the Supplementary Material.

**Scheme 2:**
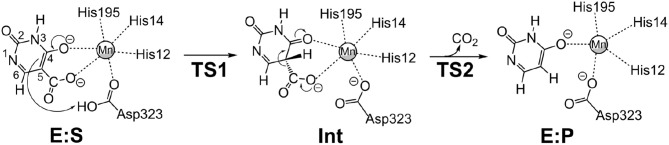
Proposed mechanism for the IDCase-catalyzed decarboxylation of 5-carboxyuracil (**1a**) on the basis of current calculations.

**Figure 3:**
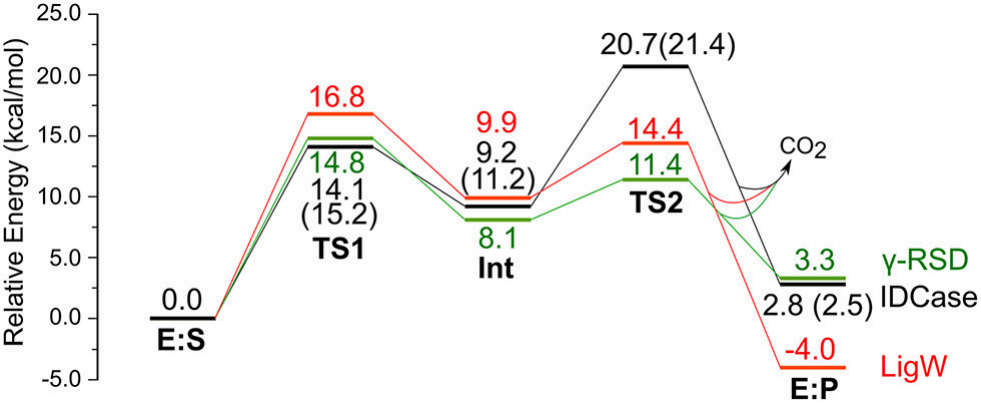
Calculated energy profiles for the decarboxylation reactions catalyzed by IDCase (black), γ-RSD (green, values taken from Sheng et al., 2018) and LigW (red, values taken from Sheng et al., 2017). The energies for IDCase with Zn instead of Mn are given in parentheses.

We envisioned that the reaction of IDCase could follow a similar mechanism as the one suggested for γ-RSD (Sheng et al., 2018) and LigW (Sheng et al., 2017), because all of them belong to cog2159 of the amidohydrolase superfamily (Seibert and Raushel, 2005). As shown in Scheme 2, the reaction would thus start with a proton transfer from Asp323 to the C5 atom of substrate, followed by C-C bond cleavage to generate CO_2_ and uracil. Overall, this sequence of events would bear a strong resemblance to those involved in the (reverse) electrophilic aromatic substitution. Indeed, this mechanistic scenario turned out to have feasible energy barriers (black line in Figure 3). The calculated barrier for the overall reaction, 20.7 kcal/mol, is in quite good agreement with the experimental value, which is ca 19 kcal/mol as converted from the experimental *k*_cat_ for IDCase from *C. militaris* (4.17 min^−1^) (Xu et al., 2013).

In the enzyme-substrate complex (**E:S** in Figure 2A), the substrate adopts a similar binding mode as in γ-RSD in complex with 2-nitroresorcinol (PDB 4QRO) (Sheng et al., 2018) and also LigW complexed with 2-nitrovanillate (PDB 4QRN) (Vladimirova et al., 2016). The barrier for the proton transfer from Asp323 to the C5 atom is calculated to be 14.1 kcal/mol, and the resulting intermediate (**Int**) is 9.2 kcal/mol higher in energy than **E:S** (Figure 3). At the transition state (**TS1**), the lengths of the breaking Asp323 O-H bond and the forming C5-H bond are both 1.34 Å (Figure 2B). The subsequent C-C bond cleavage is calculated to be the rate-limiting step with a barrier of 11.5 kcal/mol relative to **Int**, i.e., 20.7 kcal/mol higher than **E:S** (Figure 3). At **TS2**, the length of the breaking C-C bond is 2.22 Å (Figure 2D). The enzyme-product complex (**E:P**, Figure 2E) is 2.8 kcal/mol higher than **E:S** (Figure 3), including the contribution of entropy gain from the release of CO_2_.

Comparison of the calculated energy profile of the IDCase mechanism with those of LigW and γ-RSD (Figure 3) reveals some interesting features. The first step, the protonation of the substrate carbon, has very similar barriers for the three enzymes (14–17 kcal/mol), but for the subsequent C-C bond cleavage, IDCase is calculated to have a significantly higher barrier than the other two enzymes (20.7 kcal/mol for IDCase vs. 14.4 and 11.4 kcal/mol for LigW and γ-RSD, respectively). This matches the trends observed experimentally for the rate constants for these enzymes.

As discussed above, IDCase was originally suggested to be a zinc-dependent enzyme (Xu et al., 2013). Based on this, two possible mechanisms were proposed previously, both of which lead to the formation of ${\text{HCO}}_{3}^{-}$ and uracil as products (Xu et al., 2013). One mechanism involves a tetrahedral carboxylated Asp/Glu (mixed anhydride) intermediate formed by nucleophilic attack of Asp323 onto the substrate's carboxylate group, while the other one involves a hydrated carboxylate intermediate. We have examined these possibilities assuming Zn as the metal ion, but both of them turned out to be associated with prohibitively high energies and can thus be ruled out (see Supplementary Material for detailed discussion).

On the other hand, we also tested the mechanism shown in Scheme 2 with Zn instead of Mn, and the obtained barrier was only 0.7 kcal/mol higher than that with Mn (Figure 3). The optimized structures are given in the Supplementary Material. This result shows that also Zn can serve as the metal ion in IDCase, which is in stark contrast to the case of γ-RSD for which previous calculations showed that the Mn-enzyme is active while the Zn-enzyme is associated with very high energy barriers (Sheng et al., 2018).

### Substrate Specificity

**Figure 4:**
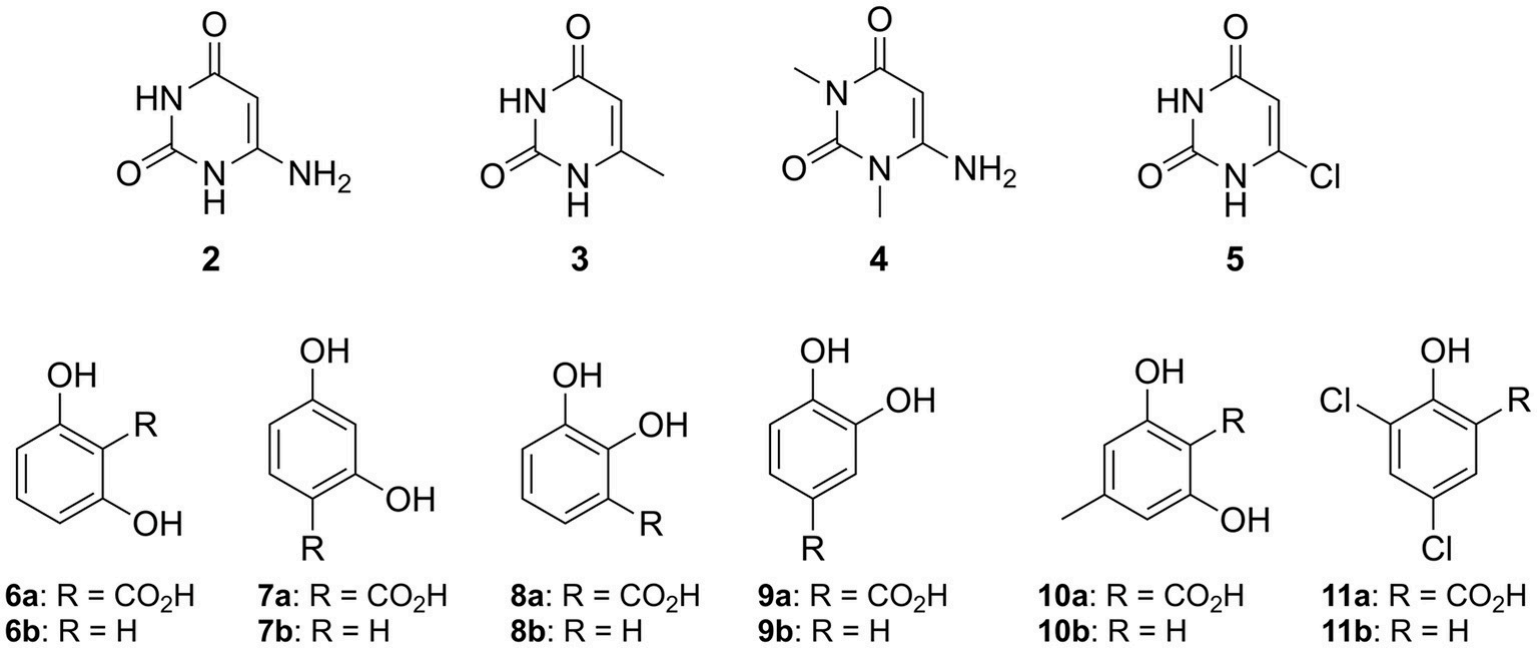
Non-natural substrates for activity screening of IDCase: Pyrimidine derivatives **(2–5)** for carboxylation and phenolic carboxylic acids **(6a−11a)** and phenols **(6b−11b)** for decarboxylation and carboxylation, respectively.

In order to explore the utility of IDCase for biocatalytic purposes, its substrate tolerance was elucidated using a range of heterocyclic and homocyclic analogs of the natural substrate [5-carboxyuracil (**1a**)] in the decarboxylation and reverse carboxylation direction, respectively (Figure 4). The activity of IDCase overexpressed in *E. coli* was verified under standard conditions in aqueous buffer pH 7.5 at 30°C by decarboxylation of 5-carboxyuracil (**1a**), which showed nearly full conversion within 24 h.

The reverse carboxylation of uracil (**1b**) using the standard carboxylation procedure in presence of 3 M bicarbonate (Wuensch et al., 2012) did not show any product formation, corroborating observations of Palmatier et al. (1970). In addition, pyrimidine derivatives (**2**–**5**), which are electronically and sterically closely related to uracil (**1b**), were investigated to explore IDCase for the carboxylation of heterocyclic compounds (Figure 4). None of them reacted.

As alternative CO_2_ source to bicarbonate, gaseous carbon dioxide under pressure (~30–40 bar) was recently successfully employed for the carboxylation of resorcinol (1,3-dihydroxybenzene) with conversion of up to 68% by *o*-benzoic acid decarboxylases (Plasch et al., 2018). Attempts to carboxylate uracil (**1b**) by IDCase using pressurized CO_2_ (30 bar) were unsuccessful.

Since the decarboxylation catalyzed by IDCase is calculated to follow a similar mechanism compared to those of γ-RSD and LigW (Sheng et al., 2017, 2018), and γ-RSD exhibited a broad substrate scope for phenols and phenolic carboxylic acids in the carboxylation and decarboxylation direction, respectively (Ishii et al., 2004; Yoshida et al., 2004; Matsui et al., 2006; Iwasaki et al., 2007; Ienaga et al., 2013; Wuensch et al., 2014), we tested whether IDCase could promote the decarboxylation of *o*-hydroxybenzoic acids **6a**–**11a**, however, without success. Furthermore, we expected that the enhanced electron-density of (*iso*-cyclic) phenols (**6b**–**11b**) compared to (heterocyclic) uracil (**1b**) might augment electrophilic aromatic substitution thereby allowing the reverse carboxylation reaction. Again, carboxylation of **6b**–**11b** failed. For reason of comparison, we performed a microwave-assisted Kolbe-Schmitt carboxylation (Stark et al., 2009) in a carbonate-based ionic liquid using the natural substrate **1b**. No product formation was detected proving that this reaction is not feasible.

In view of the structural and mechanistic similarity of IDCase with *o*-BDCs, such as γ-RSD, which show a broad substrate tolerance with up to >97% conversion toward the thermodynamically disfavored carboxylation direction (Wuensch et al., 2014; Sato et al., 2015; Plasch et al., 2017), the lack of reactivity of IDCase was puzzling. In order to explain the high substrate specificity of IDCase for 5-carboxyuracil (**1a**) and its inability to catalyze the reverse carboxylation, we inspected its active site and its mode of action in more detail.

### Sequence Alignment and Active Site Comparison

Sequence alignment of IDCase was performed with γ-RSDs (γ-RSD_Ps 27% and γ-RSD_Rs 29% identity) and LigWs (LigW_Sp 26% and LigW_Na 25% identity) by means of a fixed Arg-residue (see Supplementary Material). Despite the low sequence similarities of < 30%, striking structural similarities concerning the requirement for a divalent metal together with several conserved catalytically relevant amino acid residues in the active sites are apparent.

**Figure 5:**
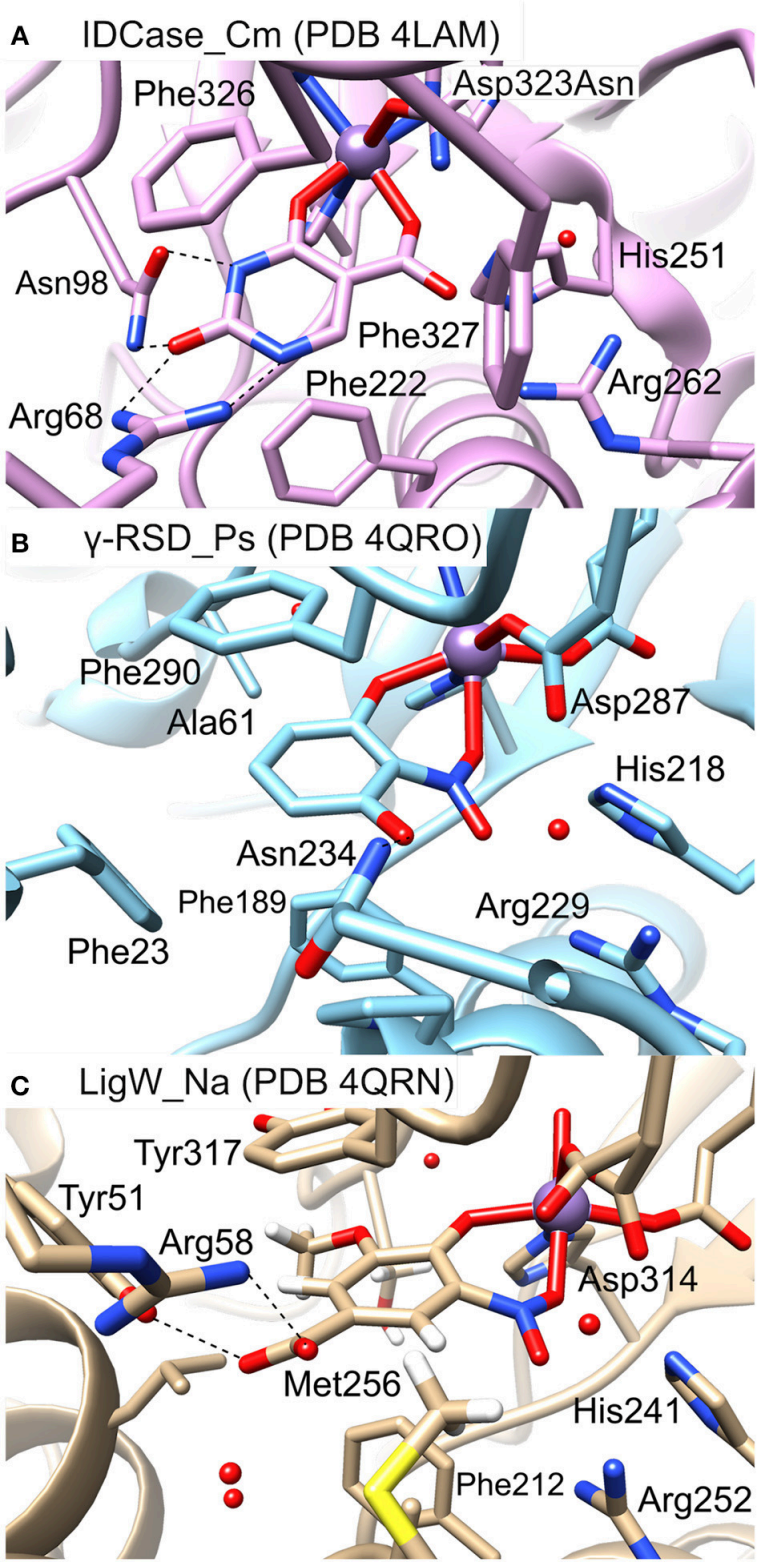
Active sites of different metal-dependent decarboxylases: **(A)** IDCase_Cm, **(B)** γ-RSD_Ps, and **(C)** LigW_Na.

In Figure 5 the active sites of IDCase_Cm, γ-RSD_Ps and LigW_Na are compared. The residues forming hydrogen bonds with the carboxylate group of 5caU in IDCase (His251, Arg262, and Asp323) are well conserved in γ-RSD (His218, Arg229, and Asp287, respectively) and LigW (His241, Arg252, and Asp314, respectively).

Three phenylalanine residues (Phe222, Phe326, and Phe327) interact with the aromatic ring of 5caU in IDCase. Two of the positions are occupied by aromatic residues in γ-RSD (Phe189 and Phe290) and LigW (Phe212 and Tyr317), while the third is either replaced by a polar residue in γ-RSD (Asn234) or replaced by Met256 in LigW. The Asn234 residue in γ-RSD assists in the substrate binding by forming a hydrogen bond with the hydroxyl group of γ-resorcylate, while the methyl group of Met256 forms a hydrophobic interaction with the aromatic proton of the 5-carboxyvanillate substrate in LigW.

Further comparison of the structures reveals important roles of the Arg68 and Asn98 residues in the substrate binding and specificity of IDCase. Namely, Arg68 forms hydrogen bonds with both N1 and the C2 carbonyl group of the substrate, while Asn98 forms hydrogen bonds with N3-H and the carbonyl group (Figure 5A). This advantageous hydrogen-bonding network between the aromatic ring of the substrate and the active site residues is missing in the case of non-natural substrates, which results in lower binding affinities for these compounds. In LigW and γ-RSD, the Arg68 and Asn98 positions are either empty or occupied by different residues. In LigW, the Tyr51 and Arg58 residues form hydrogen bonds with the C1 carboxylate group rather than the aromatic ring (Figure 5C), while in γ-RSD only Phe23 provides interaction with the aromatic ring of γ-resorcylate (Figure 5B). This analysis provides thus a basis to understand how the active sites of these enzymes are adapted to bind their respective natural substrates, which might explain the observed inability of IDCase to process non-natural substrates. Accordingly, it is conceivable that suitable mutations of the Arg68 and Asn98 residues could help to expand the substrate scope of IDCase.

### Energetic Considerations

To shed more light on the reasons for the high substrate specificity of IDCase, it is instructive to consider the different binding modes of the natural substrate and compare them to inactive non-natural substrates. In the previous study on the reaction mechanism of γ-RSD it was found that the substrate, in addition to the productive binding mode in which it binds to the metal with both the hydroxyl and the carboxylate groups (here called **Mode-A**), it can also bind in an unproductive mode only through the coordination of one oxygen atom of the carboxylate group (called **Mode-B**) (Sheng et al., 2018). Inspired by this, we wondered whether the non-natural substrates would bind to IDCase unproductively, which could explain their lack of reactivity and hence the high substrate specificity observed for this enzyme.

To examine this idea, we compared the energies of the two different binding modes for both the natural substrate 5caU (**1a**) and γ-resorcylate (**6a**) as a representative case of the non-natural substrates. Accordingly, the substrates were placed in the active site manually, and the structures were optimized and the energies evaluated.

**Figure 6:**
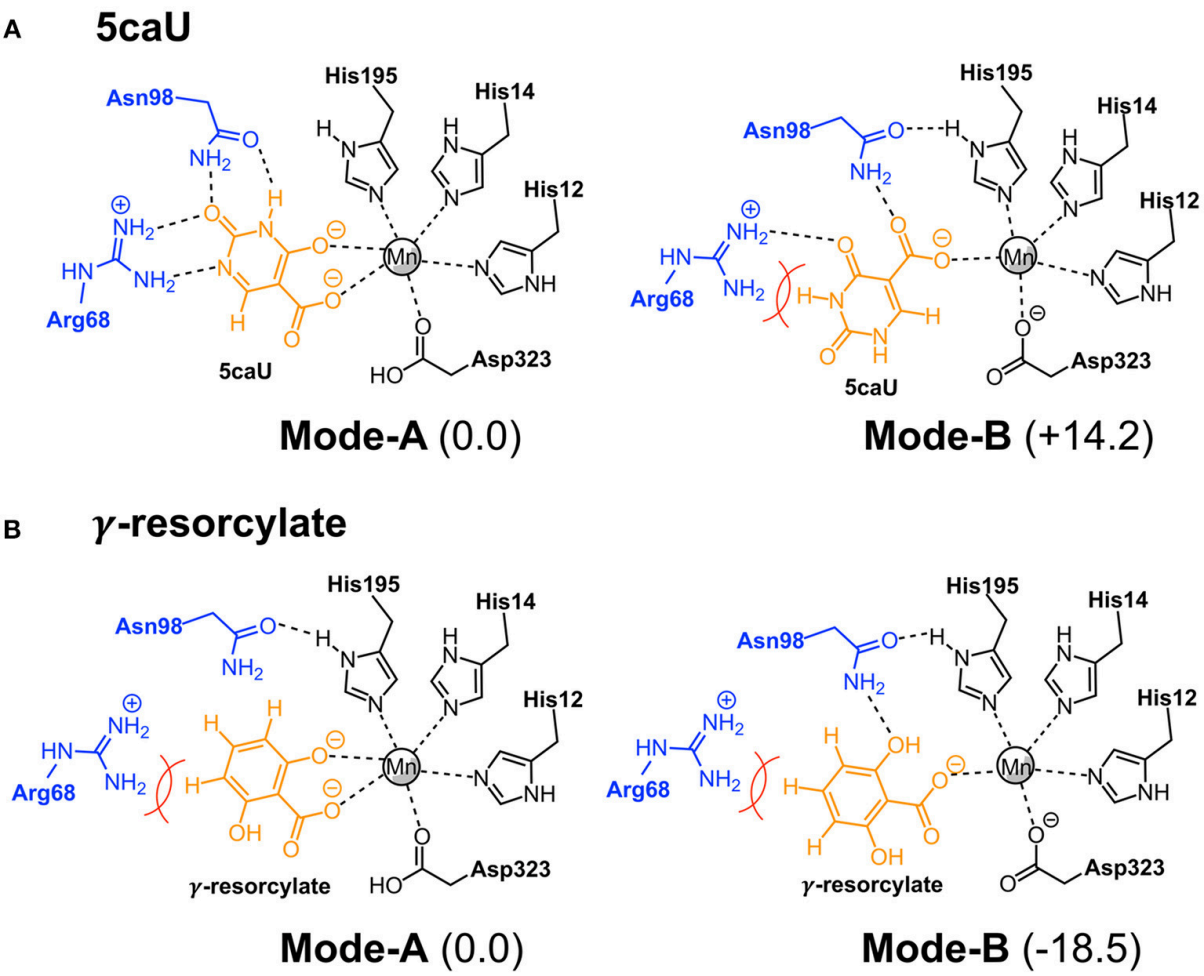
Binding modes of 5caU **(A)** and γ-resorcylate **(B)** in IDCase. Relative energies for each substrate are given in kcal/mol.

For 5caU, **Mode-A** is indeed much more favorable than **Mode-B**, with a calculated energy difference of 14.2 kcal/mol (Figure 6A). This is due to the fact that the hydrogen bonding network to the surrounding residues in **Mode-B** is not as optimal as in **Mode-A**. In particular, the hydrogen bonds to Arg68 are broken, which leads to substrate repulsion. Interestingly, in the case of γ-resorcylate the energy trend is reversed and **Mode-B** is now calculated to be 18.5 kcal/mol lower than **Mode-A** (Figure 6B). Here, Asn98 plays an important role in forming favorable hydrogen bonds to the γ-resorcylate in **Mode-B** but not in **Mode-A**. As discussed above, it was previously shown that **Mode-B**, despite its lower energy, is in fact an unproductive binding mode in the reaction of γ-RSD (Sheng et al., 2018). The situation should be similar for IDCase, which could rationalize the lack of decarboxylation activity when using γ-resorcylate and other phenolic carboxylic acids with this enzyme.

Furthermore, to gain insight into the lack of the reverse carboxylation activity of IDCase (see above), it is helpful to compare the obtained energy profile for this enzyme with those of γ-RSD and LigW. As shown in Figure 3, IDCase is calculated to have a higher barrier for the overall reaction than γ-RSD and LigW (20.7 kcal/mol for IDCase vs. 16.8 and 14.8 kcal/mol for LigW and γ-RSD, respectively). It is interesting to combine these findings about the barriers with the overall driving forces calculated for the three net reactions catalyzed by these enzymes (corresponding to the reactions of Scheme 1)^1^. The calculations show that the decarboxylation reaction of IDCase (Scheme 1A) is 11.3 and 7.5 kcal/mol more exergonic than those of γ-RSD (Scheme 1B) and LigW (Scheme 1C), respectively. This means that the barrier for the reverse carboxylation is much less favorable for IDCase compared to LigW and γ-RSD, which could explain the lack of such activity for IDCase.

## Conclusions

Combined theoretical and experimental techniques have been employed in the present study to determine the metal identity, investigate the reaction mechanism and elucidate the substrate specificity of IDCase. ICPMS/MS measurements demonstrated the IDCase from *C. militaris* contains a catalytically relevant Mn^2+^ ion rather than the previously assumed Zn^2+^ ion. Detailed analysis of the mechanism of action by quantum chemical methods revealed that decarboxylation of the natural substrate (5-carboxyuracil) proceeds via a (reverse) electrophilic aromatic substitution with formation of CO_2_, similar to that of γ-RDC and LigW, while previous proposals (yielding ${\text{HCO}}_{3}^{-}$) could be ruled out on the basis of prohibitively high energy barriers. Comparison of the crystal structure of IDCase_Cm with the structures of the related γ-RSD_Ps and LigW_Na, and an energy analysis of different substrate binding modes, suggested that the reason for the unexpected high substrate fidelity of IDCase is due to a specific substrate binding via a hydrogen-bonding network involving the N-H and C=O moieties in its natural substrate 5-carboxyuracil. In contrast to related decarboxylases acting on benzoic acids, possessing a broad substrate tolerance, the (reverse) carboxylation of uracil by IDCase is not feasible, and it is argued to be due to an enhanced energy demand of this uphill reaction.

## Author Contributions

XS performed the quantum chemical calculations. KP, SP, CE, GH, and SB performed the experimental work. WK, WG, SG, FH, and KF supervised the work. All authors contributed to the analysis of the results and to the writing of the paper.

### Conflict of Interest Statement

The authors declare that the research was conducted in the absence of any commercial or financial relationships that could be construed as a potential conflict of interest.

## Footnotes

^1^It is important to emphasize that the driving force corresponds to the net reaction catalyzed by the enzyme and should not to be confused with the energy difference between the enzyme-substrate and enzyme-product complexes.

## Supplementary Material

Data sheet 1 General experimental procedures, preparation of substrates and analytical procedures, HPLC analysis, ICP-MS measurements, structural biology, sequence alignment, computational methods, additional computational results and Cartesian coordinates of optimized structures.
