# Supplementary material for: Reaction Mechanism and Substrate Specificity of Iso-orotate Decarboxylase: A Combined Theoretical and Experimental Study
Source: Front Chem. 2018 Dec 19;6:608. doi: 10.3389/fchem.2018.00608 (PMC6305744; doi:10.3389/fchem.2018.00608)
Supplement: Data sheet 1 — General experimental procedures, preparation of substrates and analytical procedures, HPLC analysis, ICP-MS measurements, structural biology, sequence alignment, computational methods, additional computational results and Cartesian coordinates of optimized structures. [file Data_Sheet_1.PDF]

Supporting Information

Reaction Mechanism and Substrate Specificity of *Iso*-orotate Decarboxylase: A Combined Theoretical and Experimental Study

Xiang Sheng,[a] Katharina Plasch,[b] Stefan E. Payer,[b] Claudia Ertl,[b] Gerhard Hofer,[c] Walter Keller,[c] Simone Braeuer,[d] Walter Goessler,[d] Silvia M. Glueck,[b,e] Fahmi Himo,[a,]* Kurt Faber[b,]*

[a] Department of Organic Chemistry, Arrhenius Laboratory, Stockholm University, 10691 Stockholm, Sweden

[b] Institute of Chemistry, Organic & Bioorganic Chemistry, University of Graz, Heinrichstrasse 28, 8010 Graz, Austria

[c] Institute of Molecular Biosciences, University of Graz, Humboldtstrasse 50, 8010 Graz, Austria

[d] Institute of Chemistry, Analytical Chemistry, University of Graz, Universitätsplatz 1, 8010 Graz, Austria

[e] Austrian Centre of Industrial Biotechnology (ACIB GmbH), Petersgasse 14, 8010 Graz, Austria

Table of Contents

1. General biotransformation procedures....S2
2. Preparation of substrates and analytical procedures....S3
3. HPLC analysis....S3
4. ICPMS/MS measurements....S4
5. Validation of metal coordination....S4
6. Sequence alignment....S5
7. Computational methods....S6
8. Additional computational results....S7
9. References....S12
10. Cartesian Coordinates....S15

1. General biotransformation procedures

Cloning, expression and purification of IDCase *Cordyceps militaris*: see Reference [1].

Decarboxylation: Purified enzyme (50 µL IDCase) was added to TRIS-HCl buffer (650 µL, pH 7.5, 50 mM). The substrate (5 mM final concentration, dissolved in 250 µL TRIS-HCl buffer) was added to the enzyme solution (950 µL final volumes) which was transferred into a tightly sealed glass vial and the reaction mixture was allowed to react for 24 h at 30 °C with 120 rpm shaking. The reaction was stopped by adding 5-nitrouracil as inhibitor [1] [50 µL of 100 mM 5-nitrouracil stock solution in H_2O (pH 14), 5 mM final concentration] followed by centrifugation (10 min, 14000 rpm). The resulting supernatant was directly used for measurements on a reversed-phase HPLC system using NH_4OAc 20 mM, pH 5.2 as eluent (0.6 mL min^{-1}) on an achiral column (Phenomenex Luna, C18 100 Å, 250 x 4.6 mm, 5 µm, column temperature 24 °C). All screening experiments were carried out at least in triplicate.

Carboxylation: For the carboxylation with bicarbonate the rehydrated enzyme (50 µL IDCase) with substrate (5 mM final concentration, dissolved in 250 µL TRIS-HCL buffer) was transferred into a glass vial containing $KHCO_3$ (3 M, 300 mg) to give a final pH of 8.5 (950 µL final volume). The vials were tightly sealed with screw caps and were shaken for 18 h at 30 °C with 120 rpm.

For the carboxylation with 30 bar CO_2, the rehydrated enzyme (150 µL IDCase) and substrate (5 mM final concentration) were dissolved in TRIS-HCl buffer (750 µL) and were transferred into a pressure reactor with an additional gas inlet applying 30 bar CO_2 gas (2950 µL final volume). The pressure reactor was tightly sealed and the mixture was stirred at 50 rpm for 24 h at 30 °C.

Screening: The screening of phenolic compounds (**6a/b** – **11a/b**) was done with purified enzyme (50 µL IDCase), which was added to TRIS-HCl buffer (825 µL, pH 7.5, 50 mM). The substrate (10 mM) was dissolved in MeOH (50 µL, 5% v/v) and ascorbic acid (50 µL, 10 mM in H_2O, to prevent auto-oxidation) were added to the enzyme solution (1 mL final volume), which was transferred into a tightly sealed glass vial (carboxylation: with 3 M $KHCO_3$/decarboxylation: no $KHCO_3$) and the reaction mixture was shaken at 120 rpm for 24 h at 30 °C. After 24 h the reaction was stopped by adding 5-nitrouracil as inhibitor [1] [50 µL of 100 mM 5-nitrouracil stock solution in H_2O (pH 14), 5 mM final concentration]. A sample of 100 µL was diluted with 900 µL of H_2O/MeCN/trifluoroacetic acid (TFA, 50:50:3) to precipitate the enzyme, which was removed by centrifugation (10 min, 14000 rpm). The resulting supernatant was directly used for measurements on a reversed-phase HPLC system using H_2O/MeCN (0.1% TFA) as eluent (gradient: MeCN/H_2O 5%–100%) on an achiral column (Phenomenex Luna, C18 100 Å, 250 x 4.6 mm, 5 µm).

Microwave-assisted carboxylation [2]: In a high precision glass vial (2 mL) with a stirring bar (Biotage) the ionic liquid tributylmethylammonium methylcarbonate solution (50% MeOH/H_2O 2:3) and uracil (final concentration 1 M) were added in a 2.9:1 ratio and quickly closed with a

septum. The reaction was performed in the Biotage® Initiator+ microwave at 160 °C (±10 °C) for 10 min at around 10 bar. The reaction mixture was colorless at the beginning of the reaction and turned yellowish. Analysis was done with the same procedure than for the general biotransformation (see above).

2. Preparation of substrates and analytical procedures

Compounds **1a** and **1b** were purchased by Sigma Aldrich. For the synthesis of substrates **2-5** see references 3-6. For the spectra of substrate **2-5** see references 7-11. For substrates **6a/b-8a/b**, **10a/b** see reference 12 and substrates **9a/b** see reference 13. Substrates **11a/b** were a kind gift from BASF.

3. HPLC analysis

Table S1. *HPLC analysis of compounds* ***1a, 1b, 2-5***

entry	compound	retention time [min]
1	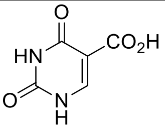 **1a**	5.6
2	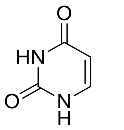 **1b**	9.6
3	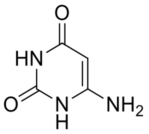 **2**	8.9
4	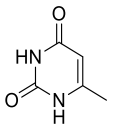 **3**	18.7
5	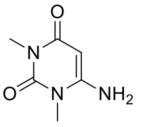 **4**	11.8
6	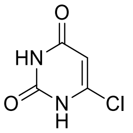 **5**	22.1

4. ICPMS/MS measurements

Metal salt experiments: Purified enzyme (25 µL, 0.85 g L^{-1}) was diluted in a TRIS-HCl buffer (400 µL, 50 mM, pH 7.4) and 25 µL of the metal salt solution of $ZnSO_4$, $MgSO_4$ and $MnSO_4$ (0.5, 0.25 and 0.05 mM, respectively) was added.

Metal analysis: The metal content of purified enzyme was measured on an ICPMS/MS (8800 ICPQQQ, Agilent Technologies, Waldbronn Germany) system in oxygen reaction mode (30 % O_2) for a better detection limit of sulfur (m/z ratios: 24-24 Mg, 55-55 Mn, 66-66 Zn, 32-48 S) with TRIS buffer (10 mM, 250 mM NaCl) as eluent (0.3 mL min^{-1} flow) on a GE Superdex S200 self-packed column (150 x 5 mm, room temperature, 10 µL injection volume). In order to avoid blockage of the ICPMS/MS interface, high matrix introduction mode was used with a four times aerosol dilution.

5. Validation of metal coordination

Estimation of metal-ligand binding distances: The metal ion binding geometry of IDCase was examined using the CheckMyMetal (CMM) Metal Binding Site Validation Server [14] on the PDB deposited structure (4HK7) and compared to the same coordinates with the Zn^{2+} ions replaced by Mn^{2+}. Metal ions not bound to the active site were removed prior to the analysis.

6. Sequence alignment

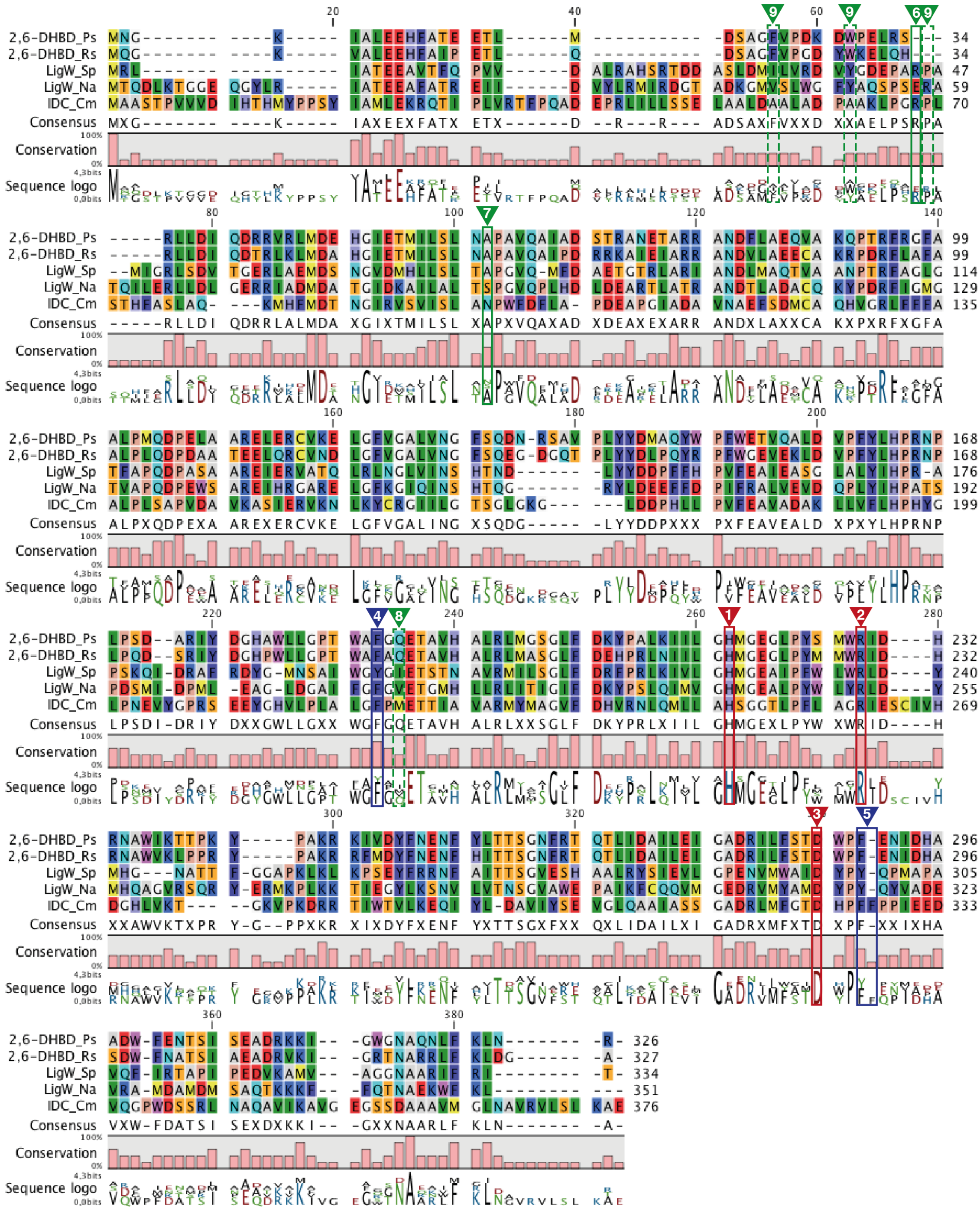

Figure S1. *Protein sequence alignment of IDCase with γ-RSD (2,6-DHBD_Ps; 2,6-DHBD_Rs) and LigWs (LigW_Sp and LigW_Na). Red boxes highlight the conserved His (1) Arg (2) and Asp (3) residues among the decarboxylases. Blue boxes highlight aromatic residues in close contact with the substrates (4,5). Green boxes highlight non-conserved Arg- (6) and Asn-residues (7) involved in substrate binding in IDC. Green dashed boxes indicate residues involved in substrate binding in γ-RSD (8) and LigW (9). Protein sequence alignment and figure were prepared with CLC Main Workbench 7.0.3.*

7. Computational methods

7.1 Technical details

Calculations were preformed employing the Gaussian 09 program [15] with the B3LYP hybrid density functional method [16,17]. Dispersion effects were described by the D3 version of Grimme's empirical method with Becke-Johnson damping [18,19] and were included in all calculations, including the geometry optimizations. Geometry optimizations were carried out with the LANL2DZ pseudopotential [20] for the metal atoms (Mn and Zn) and the 6-31G(d,p) basis set for all other atoms. Single-point energies were calculated at the same level of theory as the geometry optimization using SMD solvation model [21] with the value of dielectric constant $\varepsilon = 4$. At the same level of theory as the geometry optimization, frequency calculations were preformed to obtain zero-point energies (ZPE). To get more accurate energies, single-point calculations on the optimized structures were performed with LANL2DZ for the metal atoms (Mn and Zn) and the larger basis set 6-311+G(2d,2p) for other atoms. The presented values are the large basis set energies (which include dispersion effect) corrected for ZPE and solvation effects. Following previous studies on other decarboxylation reactions [13,22,23], the entropy gain from the release of CO_2 is estimated to be 11.1 kcal/mol, corresponding to the translational entropy for the free molecule at room temperature. This value was added to the energy of the corresponding step.

7.2 Active site model

The active site model is designed on the basis of the crystal structure of acyltransferase from *Pseudomonas protegens* in complex with MAPG (PDB id: 5MG5) [1]. The mutated Asn323 is modified back to the native Asp residue. Since the metal was identified as in fact being Mn^{2+}, the zinc ion previously proposed in the crystal structure is replaced by manganese. The model consists of the Mn^{2+} ion along with its ligands (His12, His14, His195, Asp323 and substrate), residues that potentially form hydrogen bonds with the substrate directly or via water molecules (Arg68, Asn98, His251, Arg262 and Tyr301), as well as other residues that contribute to assemble the active site (Leu46, Glu50, His214, Leu218, Phe222, Asp297, Val299, Phe326 and Phe327). Three crystallographic water molecules are also included in the model. All amino acids were truncated at the α-carbons, and hydrogen atoms were added manually. To avoid unrealistic movement during the geometry optimizations, truncated α-carbons were kept fixed at their crystallographic positions. The total size of the active site model used is 310 atoms.

8. Additional computational results

8.1. Previously suggested mechanisms with Zn ion

IDCase was originally suggested to be a zinc-dependent enzyme, and two possible mechanisms have been proposed previously, both of which lead to the formation of HCO_3^- and uracil as products [1] (Scheme S1).

In the first one (Scheme S1a), the reaction starts with a nucleophilic attack of Asp323 onto the substrate's carboxylate group, leading to the formation of a tetrahedral carboxylated Asp/Glu (mixed anhydride) intermediate. The latter collapses concomitantly with protonation of the C5 atom of the substrate, forming uracil and a carboxylated aspartate intermediate, which is cleaved by a water molecule to release HCO_3^- and regenerate Asp323. Although the Asp/Glu carboxylate residue is well-known to act as a nucleophile in carboxy-peptidases, epoxide hydrolases and haloalkane dehalogenases, nucleophilic attack of Asp onto a carboxylate group is not very likely, because they are both negatively charged and the formation of a carboxylated Asp/Glu intermediate is not a common feature within the amidohydrolase superfamily.

In the second proposal (Scheme S1b), Asp323 serves as a general base to deprotonate a water molecule generating a hydroxide ion, which then performs a nucleophilic attack onto the carboxylate group of the substrate to form a hydrated carboxylate intermediate, which collapses via C-C bond cleavage with protonation of the substrate to form HCO_3^- and uracil. Quantum chemical calculations have shown that similar doubly negatively charged oxy-anion intermediates in the reactions catalyzed by LigW [24] and phenolic acid decarboxylase [25] have prohibitively high energies.

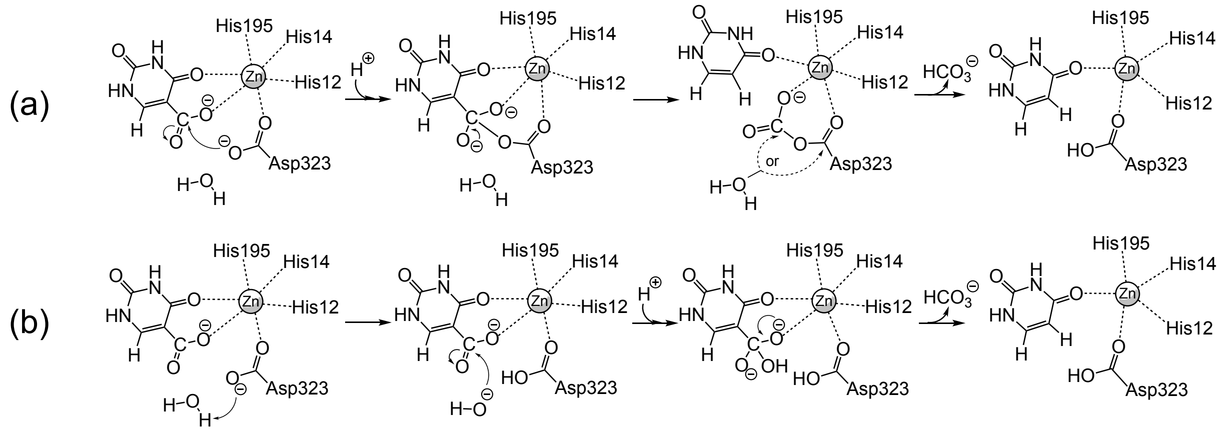

***Scheme S1**. Previously proposed mechanisms for the IDCase-catalyzed decarboxylation of 5-carboxyl uracil (**1a**).*

In order to scrutinize these two mechanisms, we optimized the structures for the enzyme-substrate complex and the structures of the proposed intermediates and their corresponding energies were evaluated (Figure S2). All attempts to create a covalent bond between Asp323 and the carboxylate group of the substrate (Scheme S1a) resulted in a fall-back into the enzyme–substrate complex. Forcing the formation of such an intermediate by constraining the C–O bond to 1.5 Å resulted in a very high energy (26.6 kcal/mol higher than enzyme−substrate complex). Regarding the scenario in Scheme S1b, the hydrated carboxylate intermediate was calculated to have an even higher energy (+40.1 kcal/mol relative to enzyme−substrate complex). Since the transition states leading to these intermediates should have even higher energies, both pathways can be ruled out.

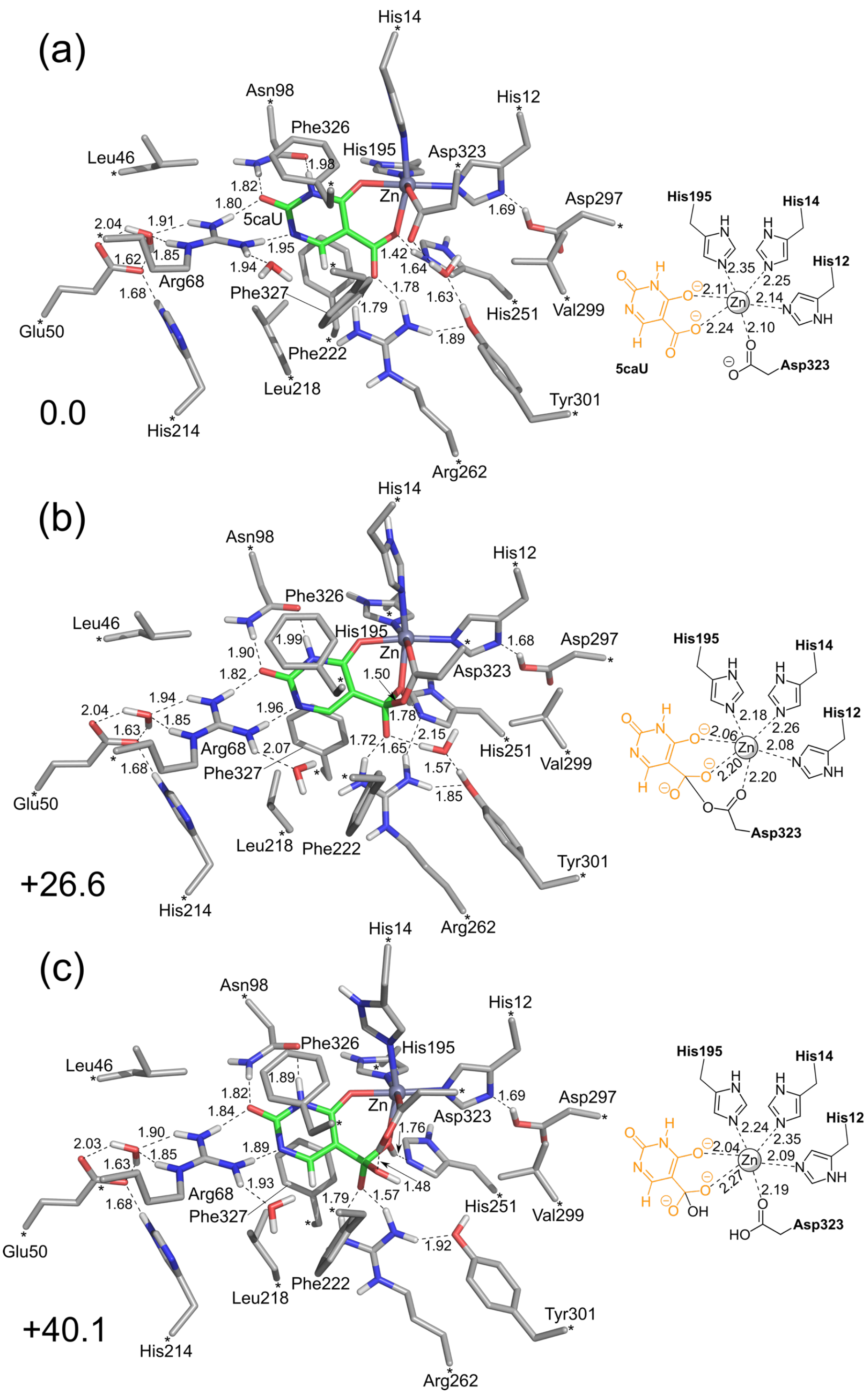

Figure S2. *Optimized structures involved in the previously suggested mechanisms: (a) the enzyme-substrate complex; (b) the intermediate formed by nucleophilic attack of Asp323 onto the carboxylate group of the substrate, with a constrained C–O bond distance of 1.5 Å; (c) the hydrated carboxylate intermediate formed by addition of water on the carboxylate group. Energies relative to the enzyme−substrate complex (a) are given in kcal/mol. Distances are given in Å.*

8.2. Alternative mechanism for IDCase with Zn instead of Mn

We have tested the mechanism shown in Scheme 2 in the main text with a Zn ion instead of Mn. The optimized structures involved in this mechanism shown in Figure S3. The obtained energies are given in Figure 3 in the main text.

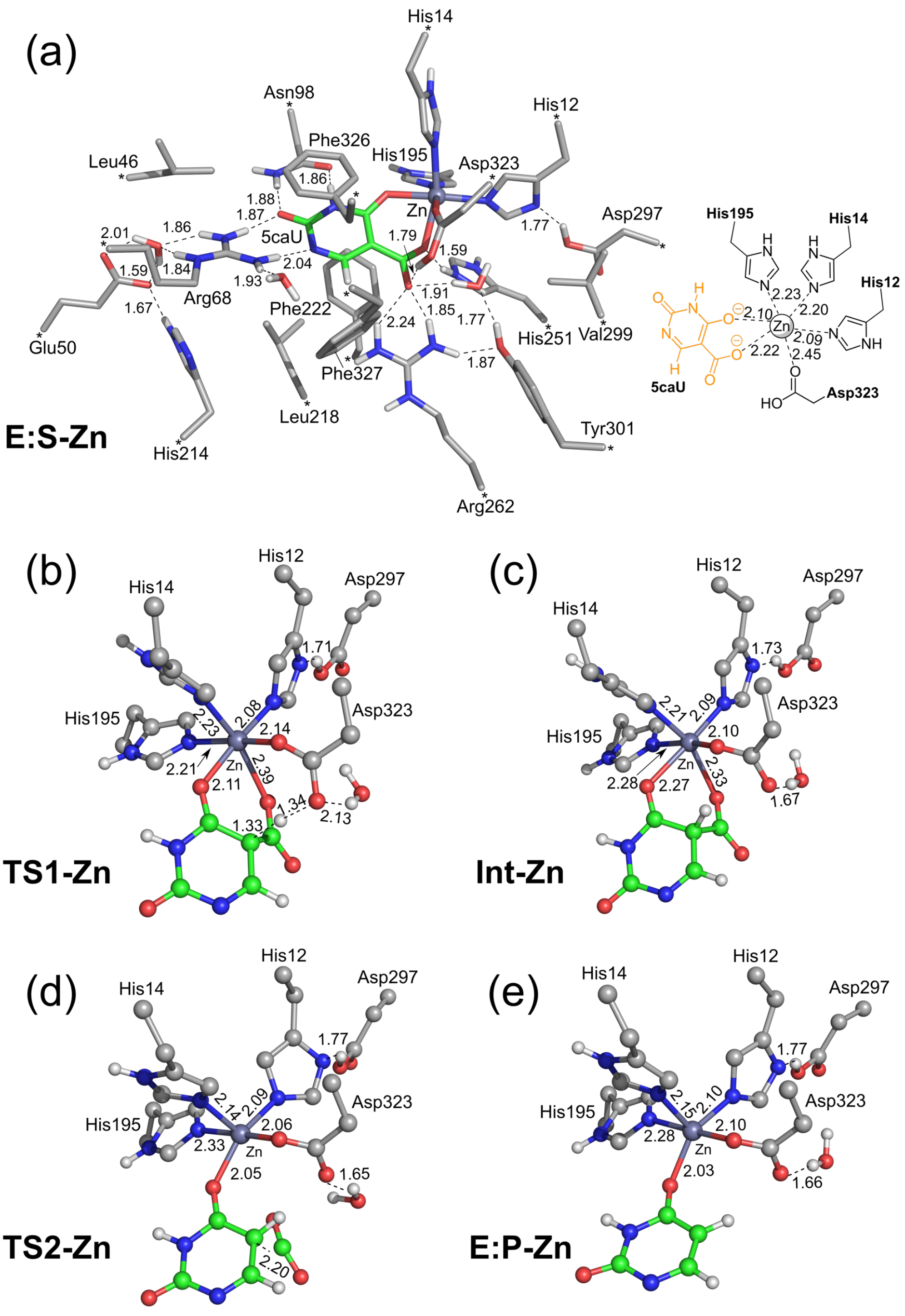

Figure S3*. Optimized structures of the transition states and intermediates along the reaction pathway as in Scheme 2 for IDCase with Zn instead of Mn. For clarity, the full model is only shown for* ***E:S-Zn****. Distances are given in Å.*

8.3. Binding modes of 5caU (1a) and γ-resorcylate (6a) in IDCase

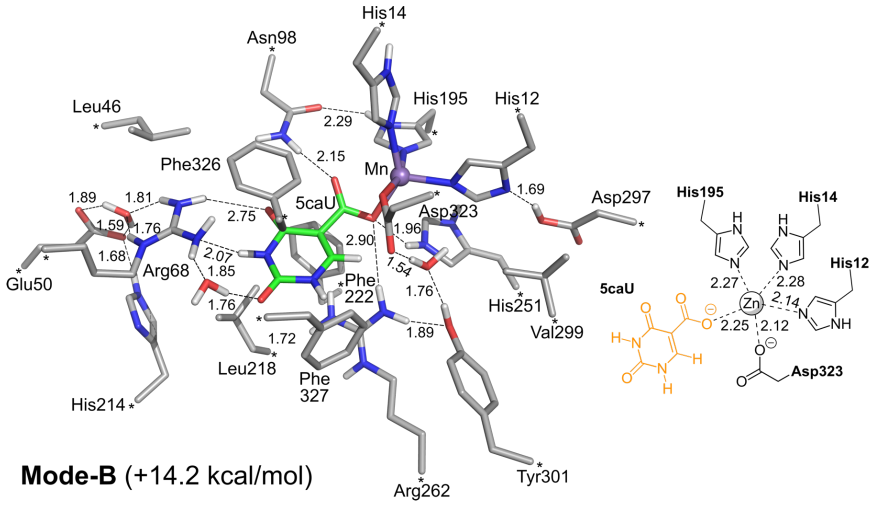

***Figure S4**. Optimized structure of the other considered binding mode (**Mode-B**) for the natural 5caU substrate (**1a**) in IDCase. Energy calculated relative to **Mode-A** is given.*

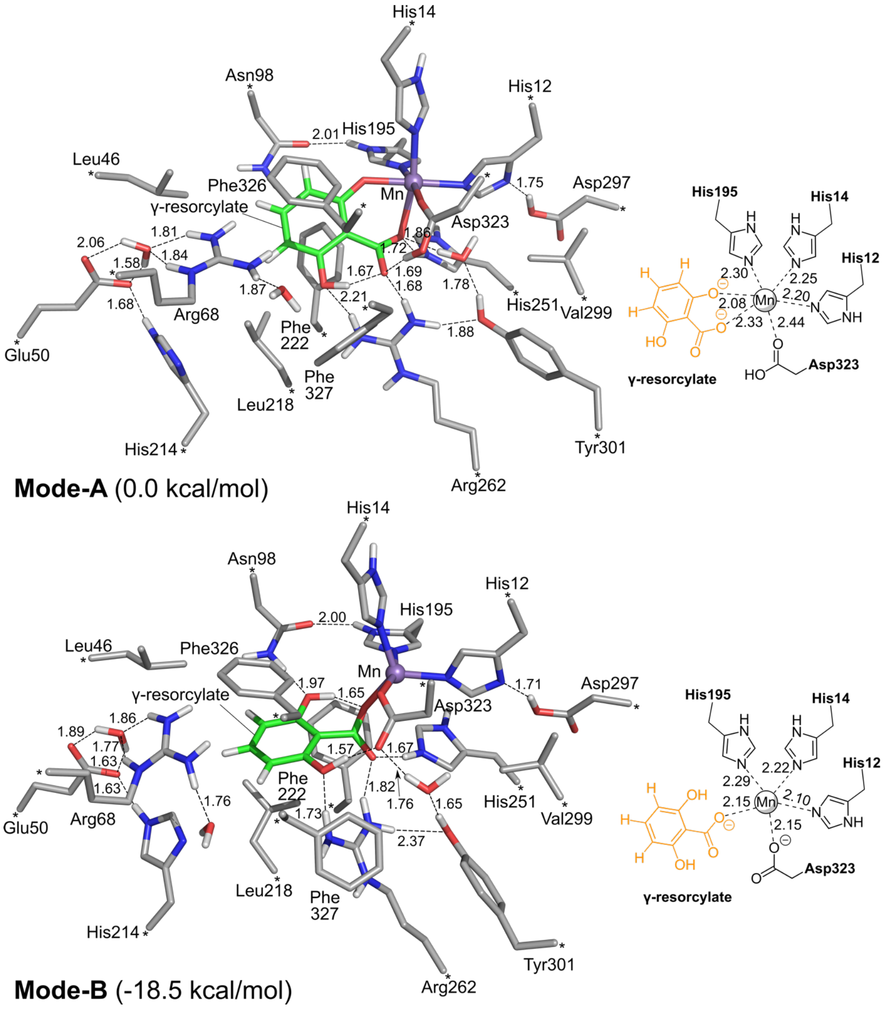

***Figure S5**. Optimized structure of two considered binding modes (**Mode-A** and **Mode-B**) for the non-natural γ-resorcylate substrate (**6a**) in IDCase. Relative energies are indicated.*

9. References

1. Xu, S.; Li, W.; Zhu, J.; Wang, R.; Li, Z.; Xu, G.-L.; Ding, J. Crystal structures of isoorotate decarboxylases reveal a novel catalytic mechanism of 5-carboxyl-uracil decarboxylation and shed light on the search for DNA decarboxylase. *Cell Res.* **2013**, *23*, 1296–1309.
2. Stark, A.; Huebschmann, S.; Sellin, M.; Kralisch, D.; Trotzki, R.; Ondruschka, B. Microwave-assisted Kolbe-Schmitt synthesis using ionic liquids or Dimcarb as reactive solvent. *Chem. Eng. Technol.* **2009**, *32*, 1730–1738.
3. Sariri, R.; Khalili, G. Synthesis of purine antiviral agents, hypoxanthine and 6-mercaptopurine. *Russ. J. Org. Chem.* **2002**, *38*, 1053–1055.
4. Novakov, I. A.; Orlinson, B. S.; Navrotskii, M. B. Desulfurization of 2-thioxo-1,2,3,4-tetrahydropyrimidin-4-ones with oxiranes and 2-haloacetonitriles. *Russ. J. Org. Chem.* **2005**, *41*, 607–609.
5. Abu-Hashem, A. A.; Hussein, H. A. R. Synthesis and antitumor activity of new pyrimidine and caffeine derivatives. *Lett. Drug Des. Disov.* **2015**, *12*, 471–478.
6. Mansurova, M.; Koay, M. S.; Gaertner, W. Synthesis and electrochemical properties of structurally modified flavin compounds. *Eur. J. Org. Chem.* **2008**, 5401–5406.
7. Nasibullina, R. A.; Gimadieva, A. R.; Yakupova, L. R.; Safiullin, R. L. Free-radical chain oxidation of 1,4-dioxane inhibited by 2-thio-6-aminouracil. *Kinet. Catal.* **2016**, *57*, 154–158.
8. Mojtahedi, M. M.; Saidi, M. R.; Shirzi, J. S.; Bolourtchian, M. Microwave promoted efficient synthesis of substituted uracils and thiouracils under solvent-free conditions. *Synth. Commun.* **2002**, *32*, 851–855.
9. Thureau, P.; Ancian, B.; Viel, S.; Thevand, A. NMR diffusion and nuclear Overhauser investigation of the hydration properties of thymine: influence of the methyl group. *Chem. Commun.* **2006**, 1884–1886.
10. Castillo, J.-C.; Quiroga, J.; Rodriguez, J.; Coquerel, Y. Time-efficient synthesis of pyrido-[2,3-d]pyrimidinones via α-oxoket-enes. *Eur. J. Org. Chem.* **2016**, 1994–1999.
11. Murthy Bandaru, S. S.; Bhilare, S.; Chrysochos, N.; Gayakhe, V.; Trentin, I.; Schulzke, C.; Kapdi, A. R. Pd/PTABS: Catalyst for room temperature amination of heteroarenes. *Org. Lett.* **2018**, *20*, 473–476.
12. Wuensch, C.; Gross, J.; Steinkellner, G.; Lyskowski, A.; Gruber, K.; Glueck, S. M.; Faber K. Regioselective *ortho*-carboxylation of phenols catalyzed by benzoic acid decarboxylases: a biocatalytic equivalent to the Kolbe–Schmitt reaction. *RSC Adv.* **2014**, *4*, 9673–9379.
13. Payer, S. E.; Marshall, S. A.; Bärland, N.; Sheng, X.; Reiter, T.; Dordic, A.; Steinkellner, G.; Wuensch, C.; Kaltwasser, S.; Fisher, K.; Rigby, S. E. J.; Macheroux, P.; Vonck, J.; Gruber, K.;

Faber, K.; Himo, F.; Leys, D.; Pavkov-Keller, T.; Glueck, S. M. Regioselective *para*-carboxylation of catechols by a prenylated flavin dependent decarboxylase. *Angew. Chem. Int. Ed.* **2017**, *56*, 13893–13897.

14. Zheng, H.; Cooper, D. R.; Porebski, P. J.; Shabalin, I. G.; Handing, K. B.; Minor, W. CheckMyMetal: a macromolecular metal-binding validation tool. *Acta Crystallogr., Sect. D: Struct. Biol.*, **2017**, *73*, 223–233.

15. Frisch, M. J.; Trucks, G. W.; Schlegel, H. B.; Scuseria, G. E.; Robb, M. A.; Cheeseman, J. R.; Scalmani, G.; Barone, V.; Mennucci, B.; Petersson, G. A.; Nakatsuji, H.; Caricato, M.; Li, X.; Hratchian, H. P.; Izmaylov, A. F.; Bloino, J.; Zheng, G.; Sonnenberg, J. L.; Hada, M.; Ehara, M.; Toyota, K.; Fukuda, R.; Hasegawa, J.; Ishida, M.; Nakajima, T.; Honda, Y.; Kitao, O.; Nakai, H.; Vreven, T.; Montgomery, J. A., Jr.; Peralta, J. E.; Ogliaro, F.; Bearpark, M.; Heyd, J. J.; Brothers, E.; Kudin, K. N.; Staroverov, Keith T.; V. N.; Kobayashi, R.; Normand, J.; Raghavachari, K.; Rendell, A.; Burant, J. C.; Iyengar, S. S.; Tomasi, J.; Cossi, M.; Rega, N.; Millam, J. M.; Klene, M.; Knox, J. E.; Cross, J. B.; Bakken, V.; Adamo, C.; Jaramillo, J.; Gomperts, R.; Stratmann, R. E.; Yazyev, O.; Austin, A. J.; Cammi, R.; Pomelli, C.; Ochterski, J. W.; Martin, R. L.; Morokuma, K.; Zakrzewski, V. G.; Voth, G. A.; Salvador, P.; Dannenberg, J. J.; Dapprich, S.; Daniels, A. D.; Farkas, O.; Foresman, J. B.; Ortiz, J. V.; Cioslowski, J.; Fox, D. J. Gaussian 09, revision D.01; Gaussian, Inc.: Wallingford, CT, **2013**.

16. Lee, C.; Yang, W.; Parr, R. G. Development of the Colle-Salvetti Correlation-energy Formula into a Functional of the Electron Density. *Phys. Rev. B* **1988**, *37*, 785−789.

17. Becke, A. D. Density functional Thermochemistry. III. The Role of Exact Exchange. *J. Chem. Phys.* **1993**, *98*, 5648−5652.

18. Grimme, S.; Antony, J.; Ehrlich, S.; Krieg, H. A Consistent and Accurate Ab Initio Parametrization of Density Functional Dispersion Correction (DFT-D) for the 94 Elements H−Pu. *J. Chem. Phys.* **2010**, *132*, 154104.

19. Grimme, S.; Ehrlich, S.; Goerigk, L. Effect of the Damping Function in Dispersion Corrected Density Functional Theory. *J. Comput. Chem.* **2011**, *32*, 1456−1465.

20. Hay, P. J.; Wadt, W. R. Ab Initio Effective Core Potentials for Molecular Calculations. Potentials for the Transition Metal Atoms Sc to Hg. *J. Chem. Phys.* **1985**, *82*, 270−283.

21. Marenich, A. V.; Cramer, C. J.; Truhlar, D. G. Universal Solvation Model Based on Solute Electron Density and on A Continuum Model of the Solvent Defined by the Bulk Dielectric Constant and Atomic Surface Tensions. *J. Phys. Chem. B* **2009**, *113*, 6378−6396.

22. Lind, M. E. S.; Himo, F. Theoretical Study of Reaction Mechanism and Stereoselectivity of Arylmalonate Decarboxylase. *ACS Catal.* **2014**, *4*, 4153−4160.

23. Sheng, X.; Lind, M. E. S.; Himo F. Theoretical Study of the Reaction Mechanism of Phenolic Acid Decarboxylase. *FEBS J.* **2015**, 282, 4703–4713.

24. Sheng, X.; Zhu, W.; Huddleston, J. P.; Xiang, D. F.; Raushel, F. M.; Richards, N. G., Himo, F. A Combined Experimental-Theoretical Study of the LigW-Catalyzed Decarboxylation of 5-Carboxyvanillate in the Metabolic Pathway for Lignin Degradation. *ACS Catal.* **2017**, *7*, 4968−4974.

25. Sheng, X.; Himo, F. Theoretical Study of Enzyme Promiscuity: Mechanisms of Hydration and Carboxylation Activities of Phenolic Acid Decarboxylase. *ACS Catal.*, **2017**, *7*, 1733–1741.

10. Cartesian Coordinates

The intermediates and transition states along the reaction pathway proposed for IDCase.

E:S (0.0 kcal/mol)

C	-7.49600000	-5.24950000	2.84280000
C	-7.99610000	-4.18020000	1.86830000
C	-6.87240000	-3.45140000	1.19800000
N	-7.06910000	-2.57320000	0.15210000
C	-5.51800000	-3.51200000	1.46470000
C	-5.84210000	-2.14120000	-0.17400000
N	-4.85100000	-2.66990000	0.58610000
C	-1.74740000	-8.03730000	5.06390000
C	-0.94410000	-6.90670000	4.39360000
C	-1.62060000	-6.23250000	3.23900000
N	-2.08750000	-6.90800000	2.11890000
C	-1.85610000	-4.90780000	2.96230000
C	-2.56700000	-5.99800000	1.23030000
N	-2.43680000	-4.77340000	1.71170000
C	10.10370000	-4.32370000	-1.98450000
C	8.61620000	-4.46540000	-1.65280000
C	8.23100000	-3.96800000	-0.24920000
C	6.70870000	-3.98890000	-0.07620000
C	8.90780000	-4.77310000	0.86610000
C	13.58990000	1.82650000	-3.98250000
C	12.82390000	0.90520000	-3.02560000
C	11.30770000	1.00480000	-3.19090000
C	10.49240000	0.17420000	-2.19320000
O	11.06880000	-0.60900000	-1.41150000
O	9.21420000	0.36780000	-2.22750000
C	10.62660000	1.25190000	4.97380000
C	9.21490000	1.04610000	4.40900000
C	8.82130000	2.10500000	3.37060000
C	7.39480000	1.96570000	2.83170000
N	7.25160000	0.71000000	2.09410000
C	6.08790000	0.25360000	1.60240000
N	4.90720000	0.72700000	2.01660000
N	6.12370000	-0.71790000	0.68510000
C	3.20480000	-7.71420000	-2.42010000
C	2.76210000	-6.37300000	-3.03670000
C	2.68550000	-5.22680000	-2.03380000
O	1.58890000	-4.83790000	-1.58370000
N	3.84800000	-4.67190000	-1.65850000
C	-3.47760000	-5.34990000	-5.77150000
C	-4.01840000	-5.87060000	-4.42930000
C	-3.35260000	-5.22530000	-3.25820000
N	-1.97610000	-5.26700000	-3.07550000
C	-3.81730000	-4.46600000	-2.21120000
C	-1.65260000	-4.55360000	-1.96940000
N	-2.75290000	-4.04860000	-1.42510000
C	7.64560000	7.12810000	-1.13490000
C	6.57710000	6.03660000	-1.31880000
C	7.15630000	4.65600000	-1.25190000
N	7.51060000	4.07440000	-0.04520000
C	7.50810000	3.81790000	-2.29180000
C	8.06960000	2.91700000	-0.37310000
N	8.09030000	2.71370000	-1.71270000
C	2.62210000	4.79200000	-3.38210000
C	3.16210000	4.27320000	-2.04290000
C	3.73110000	2.84330000	-2.06710000
C	4.25680000	2.46080000	-0.68120000
C	2.71520000	1.80700000	-2.56100000
C	0.06730000	1.96910000	-7.19220000
C	-0.02150000	2.03560000	-5.65680000
C	0.15110000	0.71500000	-4.92680000
C	1.20370000	-0.15720000	-5.23860000
C	-0.70660000	0.35880000	-3.87580000
C	1.39770000	-1.33990000	-4.52430000
C	-0.51650000	-0.82690000	-3.15980000
C	0.53830000	-1.68330000	-3.47940000
C	-7.75580000	1.30330000	-3.26660000
C	-7.05340000	0.13710000	-3.95140000
C	-5.71240000	-0.12200000	-3.35510000
N	-4.92750000	-1.21450000	-3.71380000
C	-5.01040000	0.51000000	-2.36070000
C	-3.81580000	-1.25640000	-2.96500000
N	-3.84700000	-0.21140000	-2.14460000
C	-12.40040000	-1.63260000	-1.33850000
C	-10.93160000	-1.87350000	-0.94690000
C	-9.93110000	-1.36700000	-1.98180000

O	-10.26770000	-0.82550000	-3.01830000	N	-0.30180000	3.43790000	-1.66680000
O	-8.62490000	-1.52900000	-1.71950000	Mn	-2.72630000	-2.85150000	0.51210000
C	-9.26380000	2.39530000	1.08870000	O	-1.27270000	1.33420000	0.03490000
C	-8.63100000	1.00480000	0.95110000	C	-1.21390000	0.06080000	0.07710000
C	-7.10260000	1.09890000	0.99780000	O	-2.27910000	-0.64510000	-0.11740000
C	-9.14820000	0.03730000	2.02150000	C	0.06620000	-0.59720000	0.32510000
C	-8.73060000	8.24850000	2.45060000	C	0.23360000	-2.00710000	0.10740000
C	-7.33240000	8.35140000	1.81000000	O	-0.66220000	-2.88230000	0.04450000
C	-6.78180000	7.04630000	1.26700000	N	1.52050000	-2.42220000	-0.08670000
C	-5.89980000	6.25800000	2.01800000	C	2.64980000	-1.64160000	0.07630000
C	-7.12520000	6.58940000	-0.01400000	O	3.76180000	-2.13820000	-0.15910000
C	-5.35680000	5.07460000	1.51210000	N	2.48510000	-0.33940000	0.49490000
C	-6.59650000	5.40890000	-0.53630000	C	1.25630000	0.13710000	0.54070000
C	-5.69900000	4.65600000	0.22450000	O	8.82660000	-0.52690000	0.15330000
O	-5.14290000	3.53170000	-0.35210000	O	4.06300000	2.31830000	4.29740000
C	-4.21780000	-1.82190000	4.64480000	O	-3.90270000	1.63080000	1.12370000
C	-3.45520000	-0.57470000	4.19600000	H	8.03440000	-3.89500000	-2.39020000
C	-2.36550000	-0.89380000	3.21380000	H	8.30530000	-5.51690000	-1.75840000
O	-2.28320000	-1.94810000	2.59430000	H	8.56190000	-2.92540000	-0.16890000
C	1.40640000	-0.43230000	6.16920000	H	6.40250000	-3.59220000	0.89700000
C	1.63490000	-0.18380000	4.66870000	H	6.22050000	-3.37760000	-0.84350000
C	2.01120000	-1.44790000	3.92750000	H	6.31950000	-5.01370000	-0.15020000
C	3.35800000	-1.77790000	3.72770000	H	8.63490000	-5.83470000	0.80470000
C	1.03490000	-2.33800000	3.45850000	H	9.99740000	-4.70450000	0.81250000
C	3.72080000	-2.95690000	3.07650000	H	8.60260000	-4.40750000	1.85280000
C	1.39130000	-3.52350000	2.81280000	H	10.72430000	-4.95650000	-1.34230000
C	2.73770000	-3.83620000	2.61980000	H	13.08180000	1.14440000	-1.98800000
C	1.48360000	3.30670000	6.92320000	H	13.13110000	-0.13600000	-3.17100000
C	0.33400000	3.35860000	5.91020000	H	10.97750000	2.04770000	-3.08850000
C	0.74380000	3.68040000	4.48710000	H	11.00490000	0.70330000	-4.20320000
C	1.80080000	4.55770000	4.19460000	H	13.32100000	2.87710000	-3.82130000
C	0.04560000	3.11230000	3.41420000	H	8.48420000	1.06370000	5.22990000
C	2.15870000	4.84000000	2.87180000	H	9.13900000	0.04950000	3.95840000
C	0.38260000	3.40830000	2.09260000	H	9.52580000	2.08120000	2.52860000
C	1.44980000	4.26410000	1.81560000	H	8.91290000	3.10620000	3.80860000
C	-4.39320000	9.18190000	-0.83630000	H	7.15410000	2.81870000	2.18270000
C	-4.19740000	8.04140000	-1.84200000	H	6.69060000	1.96610000	3.67260000
C	-3.06220000	7.08110000	-1.45830000	H	8.06830000	0.35920000	1.57390000
C	-2.83170000	6.01610000	-2.53850000	H	7.04250000	-1.00390000	0.34320000
N	-1.60180000	5.23600000	-2.34940000	H	5.27070000	-1.17620000	0.36770000
C	-1.50490000	4.01500000	-1.80330000	H	4.81530000	1.38300000	2.78590000
N	-2.57650000	3.31870000	-1.42200000	H	4.06060000	0.39570000	1.55290000

H	10.71840000	2.22790000	5.46320000
H	3.44800000	-6.09520000	-3.84540000
H	1.76370000	-6.47190000	-3.47060000
H	3.86480000	-3.85560000	-1.03930000
H	4.72680000	-4.98410000	-2.04120000
H	3.20580000	-8.49810000	-3.18160000
H	5.81260000	6.14530000	-0.53850000
H	6.07240000	6.17470000	-2.28340000
H	7.41730000	3.92570000	-3.36220000
H	8.50670000	1.85210000	-2.12750000
H	8.47490000	2.18270000	0.30570000
H	8.37730000	7.08950000	-1.94770000
H	3.94870000	4.95120000	-1.69560000
H	2.37620000	4.32320000	-1.27130000
H	4.58470000	2.84590000	-2.75890000
H	4.67890000	1.45320000	-0.69120000
H	5.03840000	3.14460000	-0.34240000
H	3.44560000	2.46790000	0.05730000
H	3.15450000	0.80580000	-2.57040000
H	2.35190000	2.01150000	-3.57130000
H	1.84470000	1.75430000	-1.89430000
H	1.79120000	4.18670000	-3.76860000
H	0.74600000	2.73460000	-5.29420000
H	-0.98440000	2.47240000	-5.36600000
H	1.89150000	0.09570000	-6.03950000
H	-1.52510000	1.02410000	-3.60910000
H	2.22950000	-1.98980000	-4.77930000
H	-1.17860000	-1.08230000	-2.34060000
H	0.69380000	-2.59930000	-2.91770000
H	-0.65900000	1.25770000	-7.59720000
H	-6.94980000	0.31910000	-5.02920000
H	-7.67600000	-0.75410000	-3.81840000
H	-5.25250000	1.39190000	-1.78920000
H	-3.19470000	-0.15890000	-1.30990000
H	-3.04870000	-2.01050000	-2.99590000
H	-7.22000000	2.24730000	-3.41430000
H	-10.69560000	-1.38950000	0.00910000
H	-10.73520000	-2.94210000	-0.78690000
H	-13.06740000	-2.01480000	-0.56120000
H	-8.90390000	0.59510000	-0.03040000
H	-6.65100000	0.10790000	0.88650000
H	-6.73380000	1.74770000	0.19880000

H	-6.76730000	1.52760000	1.95070000
H	-8.72080000	-0.96080000	1.88080000
H	-8.87360000	0.38510000	3.02500000
H	-10.24030000	-0.04660000	1.99160000
H	-9.02280000	2.83480000	2.06420000
H	-6.62970000	8.75040000	2.55070000
H	-7.36730000	9.08560000	0.99630000
H	-5.61690000	6.58130000	3.01630000
H	-7.81330000	7.17280000	-0.62010000
H	-4.66620000	4.48240000	2.10610000
H	-6.86990000	5.06220000	-1.52800000
H	-4.76740000	2.91810000	0.32870000
H	-8.72320000	7.55290000	3.29550000
H	2.42530000	0.56210000	4.54430000
H	0.72300000	0.24520000	4.24090000
H	4.12580000	-1.09220000	4.07010000
H	-0.01650000	-2.10640000	3.59830000
H	4.77030000	-3.18040000	2.91320000
H	0.61510000	-4.18560000	2.44180000
H	3.02020000	-4.74560000	2.09800000
H	2.32330000	-0.79140000	6.64660000
H	-0.40630000	4.09880000	6.24390000
H	-0.19010000	2.39640000	5.90360000
H	-0.77090000	2.42500000	3.61410000
H	2.34550000	5.03380000	5.00450000
H	-0.18860000	2.95540000	1.28950000
H	2.98530000	5.51440000	2.66840000
H	1.72850000	4.48590000	0.79080000
H	1.91410000	4.29710000	7.10030000
H	-3.98780000	8.46260000	-2.83460000
H	-5.12980000	7.47250000	-1.93150000
H	-2.13270000	7.64900000	-1.31850000
H	-3.28540000	6.59450000	-0.50110000
H	-2.75580000	6.49160000	-3.52190000
H	-3.67620000	5.32470000	-2.59640000
H	-0.73090000	5.71830000	-2.52130000
H	-2.36750000	2.44990000	-0.92410000
H	-3.48500000	3.73840000	-1.22880000
H	0.53480000	3.82440000	-2.07460000
H	-0.25210000	2.54750000	-1.17580000
H	-4.64080000	8.78740000	0.15430000
H	-6.87360000	-5.98840000	2.32710000

H	-8.63880000	-3.46660000	2.40220000
H	-8.63560000	-4.64040000	1.10370000
H	-8.31280000	-1.99460000	-0.87380000
H	-4.97770000	-4.09890000	2.19100000
H	-5.67460000	-1.42300000	-0.95900000
H	-1.95110000	-8.85880000	4.36810000
H	-0.71270000	-6.13050000	5.12920000
H	0.02540000	-7.30070000	4.05970000
H	-2.08300000	-7.90910000	1.99240000
H	-1.63820000	-4.04490000	3.56900000
H	-2.99550000	-6.25530000	0.27400000
H	-3.60440000	-4.26520000	-5.85170000
H	-3.89340000	-6.96020000	-4.38250000
H	-5.09420000	-5.68470000	-4.35590000
H	-1.31040000	-5.74040000	-3.66850000
H	-4.83180000	-4.19070000	-1.96010000
H	-0.64390000	-4.44500000	-1.60020000
H	-4.70970000	-2.30200000	3.79620000
H	-3.00740000	-0.03160000	5.03460000
H	-4.11610000	0.14510000	3.69300000
H	1.16600000	1.19150000	0.79170000
H	9.74360000	-0.85830000	0.06940000
H	8.76500000	-0.15670000	-0.78980000
H	4.61530000	2.79340000	4.93000000
H	3.40920000	2.96980000	3.99480000
H	-2.95100000	1.54300000	0.91090000
H	-4.27310000	0.74910000	0.98020000
O	-1.46420000	0.07830000	3.06510000
H	-0.79180000	-0.22100000	2.41480000
H	-5.17930000	-1.90410000	-4.40770000
H	14.67230000	1.73520000	-3.84460000
H	13.36680000	1.58850000	-5.02930000
H	8.18270000	6.97140000	-0.19520000
H	7.20050000	8.12920000	-1.11860000
H	3.40330000	4.76900000	-4.14880000
H	2.27650000	5.82930000	-3.30100000
H	10.87460000	0.48200000	5.71050000
H	11.37830000	1.20830000	4.17840000
H	10.43360000	-3.28620000	-1.85720000
H	10.30380000	-4.61560000	-3.02060000
H	2.52650000	-8.01560000	-1.61650000
H	4.21350000	-7.64770000	-2.00210000
H	-1.18510000	-8.45300000	5.90370000
H	-2.70670000	-7.66970000	5.43890000
H	-6.89330000	-4.80260000	3.64040000
H	-8.33050000	-5.77750000	3.31260000
H	-12.59190000	-0.56560000	-1.47350000
H	-12.63630000	-2.13070000	-2.28170000
H	-8.89220000	3.08070000	0.31910000
H	-10.35450000	2.34840000	1.00350000
H	-9.46740000	7.88490000	1.72740000
H	-9.06120000	9.22610000	2.81330000
H	-8.76800000	1.40160000	-3.65970000
H	-7.84450000	1.10950000	-2.19490000
H	1.06110000	1.65920000	-7.52670000
H	-0.13530000	2.95120000	-7.62880000
H	-2.41070000	-5.56450000	-5.89010000
H	-4.00100000	-5.81740000	-6.60960000
H	-3.54390000	-2.54860000	5.10690000
H	-4.98080000	-1.54960000	5.37790000
H	1.09470000	0.48380000	6.67850000
H	0.63320000	-1.19180000	6.32840000
H	-3.48740000	9.79060000	-0.74410000
H	-5.20940000	9.83990000	-1.14790000
H	1.13120000	2.93090000	7.88760000
H	2.28380000	2.64940000	6.56880000
H	1.62940000	-3.39250000	-0.41800000

TS1 (14.1 kcal/mol)

C	-7.45446500	-5.25602400	2.77224800
C	-7.98262600	-4.16466500	1.83454400
C	-6.87666800	-3.42043200	1.15034500
N	-7.08314600	-2.58158900	0.07329300
C	-5.52425500	-3.44041000	1.42897000
C	-5.86263900	-2.13661100	-0.26151200
N	-4.86807000	-2.61807700	0.52409700
C	-1.70338200	-8.05620100	4.97146900
C	-0.87088300	-7.03652800	4.17217100
C	-1.59364300	-6.35814900	3.04918500
N	-2.08782100	-7.02469000	1.93430200
C	-1.86939800	-5.03494200	2.80725200
C	-2.61958600	-6.10769900	1.08152500
N	-2.49865900	-4.89122800	1.58237800
C	10.15085100	-4.23927500	-2.01611900

C	8.66664600	-4.38889700	-1.67310300
C	8.28539200	-3.86851000	-0.27680000
C	6.76519000	-3.91228200	-0.08853300
C	8.98615200	-4.63712300	0.84917900
C	13.62709900	1.94021100	-3.93998400
C	12.85768900	1.01251000	-2.99127800
C	11.34160900	1.11333000	-3.15894800
C	10.52294400	0.27649000	-2.16900100
O	11.09526100	-0.51116200	-1.38898500
O	9.24451200	0.47026200	-2.20663900
C	10.65086300	1.25968200	5.00455100
C	9.23371500	1.06940100	4.44944900
C	8.82725700	2.16082900	3.45079500
C	7.40321200	2.02148800	2.90607000
N	7.27681300	0.78185800	2.13869600
C	6.11831300	0.29966300	1.66656400
N	4.93251200	0.72869600	2.11358300
N	6.16310400	-0.66146700	0.73730100
C	3.25991500	-7.63931500	-2.50058500
C	2.78687300	-6.28869700	-3.07164000
C	2.73736100	-5.16142400	-2.04751300
O	1.64859100	-4.72984000	-1.61771200
N	3.91199900	-4.66287800	-1.63230500
C	-3.42221700	-5.25199600	-5.83618400
C	-3.94504300	-5.78135400	-4.49052600
C	-3.28091100	-5.13237100	-3.32025600
N	-1.90396500	-5.16097000	-3.13821500
C	-3.75349300	-4.38847600	-2.26574800
C	-1.58963800	-4.45850600	-2.02113700
N	-2.69458700	-3.97070000	-1.47339200
C	7.66699200	7.19719700	-1.04316900
C	6.58397600	6.11749800	-1.21826300
C	7.16123800	4.73457900	-1.21880400
N	7.54764500	4.10845900	-0.04464200
C	7.49498400	3.94134100	-2.29901500
C	8.10815000	2.97029800	-0.43081700
N	8.10002500	2.82056100	-1.77788100
C	2.65208400	4.87556100	-3.32434900
C	3.07200400	4.38680000	-1.93085500
C	3.70164700	2.98238500	-1.89347000
C	4.25976200	2.67650800	-0.50070200
C	2.71824800	1.88469800	-2.31784500

C	0.10926200	2.08983300	-7.16972100
C	0.06863500	2.12570700	-5.62975600
C	0.33551400	0.80291500	-4.93233300
C	1.46481600	0.03334400	-5.24976300
C	-0.50480800	0.34286500	-3.90857100
C	1.74858200	-1.14881900	-4.56619300
C	-0.22656900	-0.84522600	-3.22475300
C	0.90319700	-1.59716300	-3.54850200
C	-7.71856200	1.36384200	-3.26425000
C	-7.03657200	0.20672800	-3.98295400
C	-5.68601500	-0.07281200	-3.41837700
N	-4.91067500	-1.15293600	-3.83012100
C	-4.96234500	0.52452900	-2.41730800
C	-3.78310900	-1.21871600	-3.10524900
N	-3.79612300	-0.20328300	-2.24994800
C	-12.35989600	-1.60321800	-1.37648000
C	-10.89063200	-1.87641600	-1.01585000
C	-9.89914000	-1.33044500	-2.03853500
O	-10.24351400	-0.75024600	-3.05201300
O	-8.59403400	-1.50679500	-1.78984400
C	-9.23579100	2.40405600	1.10049700
C	-8.65320200	0.98829000	0.98447900
C	-7.12829900	1.00440500	1.14231900
C	-9.28812000	0.03250100	2.00086200
C	-8.71721700	8.24291100	2.52825300
C	-7.41763200	8.34682500	1.70470900
C	-6.83137000	7.01932900	1.26205700
C	-5.77405900	6.41954200	1.95907000
C	-7.31337100	6.35676100	0.12330000
C	-5.19817600	5.22017100	1.53199600
C	-6.75384900	5.15671900	-0.31689100
C	-5.68756700	4.59193400	0.38575000
O	-5.11206100	3.44114600	-0.12393200
C	-4.18644600	-1.84176200	4.61749400
C	-3.31522200	-0.66169500	4.18316200
C	-2.36115900	-0.99124400	3.04960000
O	-2.39150500	-2.12114300	2.51847100
C	1.43242800	-0.45726900	6.16633300
C	1.38594700	-0.33241700	4.63315700
C	1.90492800	-1.57368300	3.94350800
C	3.27821100	-1.73495400	3.71270600
C	1.03966200	-2.60347300	3.54985300

C	3.77329900	-2.88687000	3.10193300
C	1.53100900	-3.76480900	2.94872200
C	2.90044400	-3.90875000	2.72133600
C	1.50037300	3.27327600	6.96196700
C	0.24738800	3.31854700	6.07539100
C	0.50307900	3.53045300	4.59534800
C	1.46816500	4.43973200	4.13001400
C	-0.25256600	2.83150300	3.64494900
C	1.67832200	4.63264000	2.76024900
C	-0.06587500	3.04000500	2.27736600
C	0.90759900	3.93401000	1.82779400
C	-4.37663600	9.22197600	-0.74110500
C	-4.40478800	7.98044000	-1.63771800
C	-3.18499000	7.07132000	-1.43951800
C	-3.18356300	5.89247100	-2.41914900
N	-1.90968500	5.16550500	-2.44260600
C	-1.64876200	3.96339300	-1.90199200
N	-2.58528600	3.22525800	-1.31377000
N	-0.40133400	3.46976100	-1.97948000
Mn	-2.74915000	-2.87548400	0.51601800
O	-0.97023000	1.18805500	-0.45861000
C	-1.09744500	-0.01102100	-0.12199700
O	-2.15445500	-0.70416900	-0.32460500
C	0.02899600	-0.67040200	0.64420100
C	0.25367600	-2.08437500	0.30654800
O	-0.61707000	-2.96102800	0.20348800
N	1.54044500	-2.44384700	0.02453700
C	2.65123200	-1.64441900	0.18226900
O	3.76305700	-2.08008400	-0.12162300
N	2.47381600	-0.34728000	0.66356100
C	1.26020300	0.09923300	0.78499600
O	8.84234300	-0.42790800	0.16492900
O	3.83247500	2.26091700	4.28688600
O	-3.84687800	1.38548700	1.16256400
H	8.07439500	-3.83844300	-2.41752300
H	8.36585200	-5.44529900	-1.75689900
H	8.59845400	-2.81876600	-0.22176600
H	6.46248200	-3.50274300	0.88058000
H	6.26074700	-3.32382400	-0.86376600
H	6.39405900	-4.94529800	-0.13864200
H	8.73368700	-5.70488200	0.81192900
H	10.07368100	-4.54835400	0.78472300

H	8.68196000	-4.25677800	1.83054000
H	10.77983200	-4.86253100	-1.37293400
H	13.11345700	1.24439300	-1.95145300
H	13.16507800	-0.02778400	-3.14324000
H	11.01123900	2.15557400	-3.04983200
H	11.04074800	0.81924200	-4.17391600
H	13.35855400	2.98982000	-3.77211000
H	8.51169400	1.05774100	5.27797400
H	9.15582000	0.08801200	3.96683100
H	9.53110700	2.17626700	2.60798500
H	8.90617700	3.14675200	3.92421500
H	7.15678100	2.88535100	2.27391800
H	6.69583500	1.99472300	3.74443700
H	8.09384200	0.45334700	1.60519400
H	7.08368300	-0.91781100	0.37331800
H	5.31473700	-1.11456800	0.41053700
H	4.82535100	1.40786600	2.85971900
H	4.08744100	0.39347700	1.65367400
H	10.74721700	2.22001000	5.52322000
H	3.44256500	-5.98719500	-3.89706400
H	1.77492700	-6.38434500	-3.47298500
H	3.93873300	-3.85531400	-1.00730500
H	4.78913400	-4.99863000	-1.99864100
H	3.23558500	-8.40623600	-3.27860200
H	5.85274100	6.20336400	-0.40433800
H	6.04094000	6.28846000	-2.15642100
H	7.37932100	4.09120900	-3.36187700
H	8.51751300	1.98366700	-2.23714900
H	8.53726900	2.21470700	0.20862900
H	8.38557300	7.15686800	-1.86752600
H	3.79284500	5.10039100	-1.51691000
H	2.20636100	4.40559200	-1.25031800
H	4.54723800	2.98361100	-2.59539300
H	4.71231600	1.68249800	-0.47985300
H	5.02877700	3.39365400	-0.20413000
H	3.46047000	2.69030200	0.25197800
H	3.20547700	0.90549500	-2.33151000
H	2.29556900	2.04438500	-3.31321500
H	1.88995600	1.80940100	-1.60175500
H	1.91322600	4.21592900	-3.79963000
H	0.81310900	2.85683100	-5.28322000
H	-0.90469400	2.50751900	-5.29890500

H	2.14256700	0.37071400	-6.02841200
H	-1.37657800	0.92989700	-3.63013900
H	2.63790600	-1.71634400	-4.82371300
H	-0.88300600	-1.18283500	-2.42967000
H	1.12455800	-2.51517100	-3.01133800
H	-0.63182600	1.38852500	-7.56513100
H	-6.95203400	0.40932800	-5.05875300
H	-7.66337400	-0.68220400	-3.85568900
H	-5.19029100	1.38434900	-1.80750400
H	-3.10401900	-0.17425500	-1.43842400
H	-3.01646500	-1.97023600	-3.17745100
H	-7.18099500	2.30771200	-3.40810600
H	-10.63763800	-1.44363600	-0.04030700
H	-10.70277300	-2.95359800	-0.91277500
H	-13.02232500	-2.01917600	-0.61259100
H	-8.87481100	0.60388100	-0.02014300
H	-6.72302900	-0.00986900	1.07121100
H	-6.66546900	1.62349400	0.36864300
H	-6.84149500	1.42232300	2.11551100
H	-8.89516600	-0.98196600	1.87546000
H	-9.06924600	0.35388300	3.02657200
H	-10.37785900	-0.00272700	1.89329900
H	-9.04686100	2.81940500	2.09773600
H	-6.66530200	8.88637300	2.29097400
H	-7.60889100	8.96019800	0.81603000
H	-5.37916000	6.90612600	2.84690100
H	-8.13702900	6.79172600	-0.43656000
H	-4.36793300	4.78091400	2.07845800
H	-7.13222700	4.65021800	-1.19908100
H	-4.72818100	2.86704300	0.57664900
H	-8.55279100	7.67256600	3.44767900
H	1.98775300	0.53126500	4.33300400
H	0.35852400	-0.13767700	4.31063600
H	3.95897500	-0.93817500	3.99455900
H	-0.02969400	-2.48938400	3.70047900
H	4.83799300	-2.97916000	2.91050800
H	0.83769500	-4.53989900	2.63652900
H	3.28516000	-4.80067700	2.23560100
H	2.46106100	-0.59587300	6.51316200
H	-0.41609700	4.11570500	6.43804200
H	-0.31510300	2.38601900	6.19422400
H	-0.98975400	2.10386000	3.96870800
H	2.05319700	5.01549700	4.84182800
H	-0.66617300	2.49132100	1.55890000
H	2.43649600	5.33372000	2.42404000
H	1.06238800	4.08643600	0.76413600
H	2.00828800	4.24233400	6.99519200
H	-4.45018500	8.29146300	-2.69023700
H	-5.31621200	7.40660600	-1.43906600
H	-2.26616800	7.65489500	-1.58935200
H	-3.15782400	6.69267400	-0.41096600
H	-3.36950000	6.25202400	-3.43729900
H	-3.98836100	5.19101200	-2.18930000
H	-1.10911600	5.68664900	-2.77088800
H	-2.26360200	2.34755300	-0.88788800
H	-3.49523600	3.58568400	-1.03039300
H	0.31119300	3.90206400	-2.54521600
H	-0.21650300	2.55597900	-1.57341700
H	-4.37384300	8.93720400	0.31621800
H	-6.83838200	-5.97650800	2.22434000
H	-8.61399100	-3.46665600	2.40208300
H	-8.63946400	-4.60876800	1.07561500
H	-8.28586700	-1.99674900	-0.95200800
H	-4.97676700	-3.98599900	2.18147400
H	-5.70239200	-1.44188900	-1.07005900
H	-2.05305100	-8.87889100	4.33817100
H	-0.50775400	-6.25006500	4.84077700
H	0.02658200	-7.53083600	3.77625100
H	-2.06715400	-8.02304900	1.78903600
H	-1.65511300	-4.17512200	3.41922100
H	-3.07365800	-6.35612600	0.13429200
H	-3.56558200	-4.16922000	-5.91501300
H	-3.80640300	-6.86948200	-4.44746200
H	-5.02257400	-5.60935000	-4.40857000
H	-1.23413600	-5.62360200	-3.73488800
H	-4.77053800	-4.12386600	-2.01332600
H	-0.58244100	-4.35418800	-1.64703900
H	-4.82125000	-2.17893300	3.79519000
H	-2.71535300	-0.26785700	5.01219700
H	-3.93164700	0.18009400	3.84064100
H	1.15957700	1.14292000	1.07080700
H	9.75799100	-0.76479300	0.08878700
H	8.79218400	-0.05453600	-0.77980200
H	4.23522500	2.68446800	5.05431700

H	3.14887900	2.88251900	3.98879000
H	-3.01125200	1.16805400	1.60733100
H	-4.27918100	0.53443400	1.00748400
O	-1.54796500	-0.05710600	2.68017900
H	-0.66989900	-0.49647600	1.77532100
H	-5.17893600	-1.81773100	-4.54176300
H	14.70894300	1.84685100	-3.79960500
H	13.40663100	1.70953600	-4.98903700
H	8.21671900	7.02809600	-0.11295500
H	7.23253700	8.20257800	-1.01270700
H	3.51211100	4.90458600	-4.00107100
H	2.23080800	5.88662800	-3.28694100
H	10.90788300	0.46765400	5.71423900
H	11.39371500	1.24173200	4.19991400
H	10.47455900	-3.19867300	-1.89808500
H	10.34614600	-4.53797600	-3.05118200
H	2.61475500	-7.96264000	-1.67849700
H	4.28385000	-7.57822100	-2.12053000
H	-1.10233600	-8.49020400	5.77462500
H	-2.58206600	-7.58047900	5.41611100
H	-6.83801800	-4.82542700	3.56815900
H	-8.27609700	-5.80018700	3.24642200
H	-12.54376000	-0.52899400	-1.45349900
H	-12.61117600	-2.04911800	-2.34148500
H	-8.78242700	3.08394700	0.37143300
H	-10.31855600	2.40373900	0.93785000
H	-9.50347000	7.73695700	1.95902600
H	-9.07957500	9.23807700	2.80117600
H	-8.73830900	1.47295200	-3.63461600
H	-7.78679500	1.15319800	-2.19401600
H	1.09127800	1.78087500	-7.53847800
H	-0.10255000	3.08086200	-7.58099000
H	-2.35305700	-5.45074200	-5.96291300
H	-3.94507400	-5.72664100	-6.67050900
H	-3.57032100	-2.68773400	4.93356900
H	-4.82711700	-1.55111000	5.45453500
H	1.02892700	0.43835600	6.64711300
H	0.84951000	-1.31937800	6.50712400
H	-3.48627100	9.83094700	-0.93063100
H	-5.25560500	9.84938300	-0.91401900
H	1.23508700	3.01058400	7.98954800
H	2.21149400	2.52896800	6.59011400
H	1.66380700	-3.38326700	-0.38992200

Int (9.2 kcal/mol)

C	-7.47878200	-5.24168600	2.82038200
C	-7.98308900	-4.13893900	1.88056800
C	-6.86725700	-3.39384200	1.21026700
N	-7.06178000	-2.57158900	0.11783100
C	-5.52002300	-3.39323100	1.51607800
C	-5.83900700	-2.11618700	-0.19772000
N	-4.85468100	-2.57628200	0.61397800
C	-1.74124000	-7.98037000	5.12949500
C	-0.96566700	-6.80651600	4.50290200
C	-1.63702100	-6.14362300	3.33861900
N	-2.08996600	-6.83429800	2.22145200
C	-1.88733000	-4.82349900	3.05071600
C	-2.57905400	-5.93686400	1.32426600
N	-2.46530800	-4.70925300	1.79781200
C	10.17342700	-4.23624700	-1.79422800
C	8.65879800	-4.35599800	-1.60739200
C	8.15396800	-3.91255900	-0.22305900
C	6.62183800	-3.88409700	-0.20049300
C	8.68937500	-4.79813800	0.90787900
C	13.64660300	1.92324900	-3.78661500
C	12.87794800	0.98768900	-2.84659900
C	11.36339600	1.07807900	-3.02940000
C	10.54363300	0.23798200	-2.04517700
O	11.11328000	-0.55508900	-1.26942700
O	9.26478500	0.43524700	-2.08147800
C	10.57592500	1.38453000	5.13558700
C	9.18388500	1.13838000	4.54079800
C	8.78232000	2.19327900	3.50211900
C	7.37124600	2.02173900	2.93617300
N	7.26968100	0.76875700	2.18391900
C	6.12412300	0.31262700	1.66117500
N	4.93606500	0.80458800	2.02453400
N	6.18033900	-0.69045100	0.77215100
C	3.30138800	-7.67177000	-2.29451100
C	2.88450400	-6.44695400	-3.13394100
C	2.81936200	-5.15864000	-2.32607200
O	1.74861800	-4.77277600	-1.81175800
N	3.96204100	-4.46867200	-2.19000700
C	-3.35318300	-5.36932700	-5.74270800

C	-3.99894300	-5.68675500	-4.38175900
C	-3.30301400	-5.04745400	-3.22027800
N	-1.92733200	-5.12038300	-3.04660400
C	-3.74827000	-4.32563900	-2.13609600
C	-1.58984700	-4.46229500	-1.90884000
N	-2.67488300	-3.96039000	-1.33434900
C	7.63515200	7.20511700	-1.04538900
C	6.58093400	6.09548200	-1.20750500
C	7.18485600	4.72375300	-1.18095900
N	7.57846100	4.12385400	0.00472100
C	7.52110600	3.91060300	-2.24560400
C	8.14484900	2.98149400	-0.35980200
N	8.13375600	2.80396400	-1.70351000
C	2.65426100	4.82434100	-3.33970000
C	3.11776900	4.30900400	-1.97036900
C	3.75572300	2.90808700	-1.98280900
C	4.35584400	2.58059200	-0.61434900
C	2.76876600	1.81039700	-2.39864900
C	0.16405800	1.96250800	-7.16446500
C	0.28496000	2.00777900	-5.62996500
C	0.49217700	0.66115600	-4.96241500
C	1.57234100	-0.15782300	-5.32456600
C	-0.35205400	0.22036500	-3.93446000
C	1.79980200	-1.37600600	-4.68613600
C	-0.12865000	-1.00226500	-3.29308500
C	0.94684500	-1.80783900	-3.66767000
C	-7.70239000	1.27230900	-3.33160000
C	-6.98294700	0.10444600	-3.99595500
C	-5.63871800	-0.12221300	-3.39445200
N	-4.83640800	-1.20984200	-3.72929700
C	-4.94933400	0.54424100	-2.41351900
C	-3.72406900	-1.21499500	-2.97972700
N	-3.77669800	-0.15392500	-2.18338200
C	-12.35282700	-1.68057800	-1.44342500
C	-10.88829700	-1.92575900	-1.04179200
C	-9.88034600	-1.40350500	-2.06130300
O	-10.21077700	-0.85788100	-3.09821700
O	-8.57773200	-1.55851800	-1.78419200
C	-9.27127100	2.38124500	0.99776200
C	-8.70696200	0.95578900	0.96543700
C	-7.18771000	0.97510200	1.15618300
C	-9.37422400	0.05548800	2.01064800

C	-8.79083900	8.24583800	2.33021800
C	-7.42250500	8.29793500	1.61969100
C	-6.87327400	6.96349300	1.14537700
C	-5.78153800	6.35577100	1.78007200
C	-7.40798300	6.31438700	0.02182100
C	-5.22080900	5.16579000	1.30625500
C	-6.86397600	5.12465200	-0.46359400
C	-5.76021900	4.55274100	0.17425400
O	-5.19826000	3.42015300	-0.38880200
C	-4.24375900	-1.78298800	4.64178900
C	-3.27019200	-0.62935800	4.39024200
C	-2.59254200	-0.66177500	3.02321100
O	-2.29700600	-1.81241100	2.54441400
C	1.35132400	-0.34944900	6.22642800
C	1.18300100	-0.21600400	4.70521400
C	1.81569400	-1.36929700	3.96243300
C	3.19787100	-1.39253300	3.73534900
C	1.04483000	-2.44861000	3.50948600
C	3.79266200	-2.45133800	3.04888400
C	1.63740400	-3.52245300	2.84123000
C	3.01332500	-3.52181300	2.60139200
C	1.39727900	3.39456500	6.95901900
C	0.14956300	3.49806900	6.06950400
C	0.42215000	3.54138700	4.57834600
C	1.45914300	4.32143700	4.03699400
C	-0.37805100	2.80784200	3.69324300
C	1.70058900	4.34690100	2.65858200
C	-0.15556400	2.84492700	2.31573700
C	0.89329300	3.60385700	1.79301600
C	-4.41876200	9.18609900	-0.90842700
C	-4.42809400	7.88389200	-1.71598000
C	-3.17057700	7.02997100	-1.50348500
C	-3.17703400	5.77796900	-2.38762100
N	-1.88533000	5.07985200	-2.40075700
C	-1.59376300	3.88067900	-1.87073700
N	-2.50749900	3.12373100	-1.27196000
N	-0.34013800	3.40831300	-1.96827800
Mn	-2.71391300	-2.74843000	0.67824400
O	-0.73381800	1.06497800	-0.44442200
C	-0.94538600	-0.11488500	-0.10676000
O	-1.95879400	-0.81376100	-0.40792800
C	0.12317800	-0.82572000	0.78099900

C	0.38273600	-2.20721900	0.27029400
O	-0.45678800	-3.10502000	0.29484100
N	1.60436000	-2.42483000	-0.29944300
C	2.69301800	-1.58435700	-0.16295900
O	3.77708700	-1.89186500	-0.64686500
N	2.54239600	-0.37714200	0.54614700
C	1.37269400	-0.03466700	0.95266800
O	8.85574700	-0.47529500	0.27241500
O	3.79419900	2.15002000	4.29524600
O	-4.02079500	1.25498100	0.58615800
H	8.16138100	-3.73920400	-2.36891800
H	8.33664800	-5.39368900	-1.78839000
H	8.51164000	-2.88887700	-0.05753000
H	6.23411500	-3.54319200	0.76523300
H	6.23436000	-3.20440000	-0.96782500
H	6.20858800	-4.88457100	-0.38772100
H	8.37617800	-5.84127600	0.77015200
H	9.78135500	-4.78119500	0.95443800
H	8.31250000	-4.46276300	1.88040000
H	10.71985600	-4.88873400	-1.10610700
H	13.12200100	1.22041900	-1.80414600
H	13.19473100	-0.04991200	-2.99710800
H	11.02503100	2.11825800	-2.92643600
H	11.07452500	0.77882100	-4.04640000
H	13.36753600	2.97014300	-3.61903100
H	8.43588400	1.12861000	5.34593200
H	9.14836200	0.14270100	4.08273800
H	9.50311900	2.19603700	2.67376700
H	8.83742600	3.19315900	3.94876100
H	7.12215500	2.87152300	2.28639700
H	6.65068100	1.99855900	3.76358200
H	8.10312500	0.42380100	1.68526700
H	7.10974400	-0.94324800	0.42259500
H	5.34599500	-1.00763900	0.29258300
H	4.80923200	1.44723600	2.79977600
H	4.10506800	0.43223400	1.57278400
H	10.62683000	2.36024100	5.63134800
H	3.57826200	-6.31754100	-3.97182800
H	1.88661500	-6.60492000	-3.55135800
H	3.98783900	-3.61015300	-1.63924100
H	4.83142400	-4.80860800	-2.57092100
H	3.29580200	-8.57161200	-2.91414300
H	5.84213200	6.17874400	-0.40016400
H	6.04021300	6.23736800	-2.15180000
H	7.39956600	4.03698100	-3.31082800
H	8.54823500	1.95675300	-2.14559900
H	8.58101700	2.24225400	0.29424300
H	8.36040800	7.16839900	-1.86406200
H	3.84842900	5.01709000	-1.56448700
H	2.27600800	4.31029300	-1.26016000
H	4.57862400	2.93005300	-2.71088600
H	4.82315800	1.59308100	-0.62672000
H	5.12010900	3.30302200	-0.31979400
H	3.57563400	2.56539500	0.15720100
H	3.26563600	0.83673800	-2.45082000
H	2.31460000	1.98889400	-3.37598400
H	1.96304900	1.71057300	-1.66007100
H	1.89879300	4.17696600	-3.80498800
H	1.12873800	2.66166600	-5.36932800
H	-0.60870600	2.47985600	-5.20452900
H	2.25425700	0.16928600	-6.10442300
H	-1.18114200	0.84853900	-3.61796100
H	2.65012400	-1.98559400	-4.97730300
H	-0.78347700	-1.31858500	-2.48810300
H	1.12194500	-2.75622000	-3.16931500
H	-0.68431300	1.34349500	-7.47229500
H	-6.88197000	0.27034000	-5.07662400
H	-7.59272900	-0.79307000	-3.84832900
H	-5.20193800	1.43417200	-1.86065000
H	-3.09558000	-0.06335000	-1.39470200
H	-2.94561200	-1.95672400	-2.98724500
H	-7.18064300	2.22122000	-3.49724600
H	-10.66137900	-1.45727300	-0.07613800
H	-10.69211500	-2.99670600	-0.89756500
H	-13.02736100	-2.07681200	-0.67965300
H	-8.90593100	0.52506800	-0.02502800
H	-6.78496000	-0.04271600	1.13859700
H	-6.71728900	1.56055500	0.36172200
H	-6.91881500	1.43702700	2.11439800
H	-8.98628600	-0.96667500	1.94521100
H	-9.17962600	0.42497700	3.02489000
H	-10.46095200	0.02132400	1.87594900
H	-9.09632600	2.84570700	1.97572100
H	-6.68927600	8.75860600	2.29105800

H	-7.50250000	8.96769400	0.75428000
H	-5.34466800	6.82984100	2.65497600
H	-8.25765800	6.75365700	-0.49417800
H	-4.36249000	4.72367500	1.80497100
H	-7.27998600	4.63282300	-1.33703600
H	-4.84844800	2.77045600	0.27274100
H	-8.74094500	7.62829600	3.23227200
H	1.64173400	0.72267700	4.38220900
H	0.11839200	-0.15329100	4.45234900
H	3.80103700	-0.55657500	4.07264100
H	-0.03200600	-2.42917900	3.65126900
H	4.86013500	-2.43413200	2.85124100
H	1.01448400	-4.33960900	2.49005200
H	3.47702500	-4.34285400	2.06243700
H	2.41129800	-0.34719200	6.49844300
H	-0.41747400	4.39427900	6.35603000
H	-0.51238500	2.64916100	6.27202200
H	-1.18730000	2.18846800	4.06759800
H	2.07461700	4.92989900	4.69433300
H	-0.79041300	2.25836200	1.66294900
H	2.51221700	4.95093900	2.26326200
H	1.07592600	3.62093700	0.72292300
H	2.00718100	4.30211200	6.90831000
H	-4.52092300	8.11965100	-2.78489100
H	-5.31092400	7.29440700	-1.44726400
H	-2.27826000	7.62684000	-1.73845400
H	-3.08777500	6.73167200	-0.45201200
H	-3.41054400	6.05230300	-3.42269500
H	-3.95606900	5.08365700	-2.06888200
H	-1.10533000	5.60150900	-2.77431000
H	-2.18102400	2.25602800	-0.84333100
H	-3.44981300	3.44597700	-1.05773200
H	0.37783600	3.89178900	-2.48280400
H	-0.13005700	2.49946100	-1.56531900
H	-4.37657100	8.97766300	0.16579100
H	-6.86343800	-5.96614500	2.27677000
H	-8.61721400	-3.44074700	2.44508000
H	-8.63381900	-4.57350200	1.11097500
H	-8.27223200	-2.02354800	-0.93180000
H	-4.98489700	-3.91585600	2.29308900
H	-5.66897600	-1.43094200	-1.01283500
H	-1.88618800	-8.79936300	4.41601100
H	-0.79005700	-6.03401700	5.25763500
H	0.02909500	-7.15596100	4.19396800
H	-2.08021200	-7.83671700	2.10726200
H	-1.69383700	-3.94463200	3.64322400
H	-2.99980500	-6.20578300	0.36769500
H	-3.30929000	-4.28989800	-5.92074200
H	-4.02780700	-6.77569800	-4.24315100
H	-5.04283000	-5.35737200	-4.37261800
H	-1.27139500	-5.57703300	-3.66262200
H	-4.75627100	-4.04214700	-1.86810600
H	-0.57803000	-4.39488900	-1.54310500
H	-5.05014400	-1.78634500	3.90231200
H	-2.46521300	-0.64740500	5.13927800
H	-3.76278300	0.34179500	4.49276700
H	1.28540200	0.90990600	1.48492400
H	9.76579900	-0.82730800	0.20430900
H	8.82203500	-0.09094100	-0.67175900
H	4.16296800	2.52108400	5.10580300
H	3.10125900	2.77529100	4.02484800
H	-3.35484800	1.04597300	1.29165100
H	-4.67706400	0.54825300	0.67053600
O	-2.31224700	0.42895400	2.46196000
H	-0.39871600	-0.91337400	1.74150000
H	-5.07952000	-1.92343000	-4.40137800
H	14.72808700	1.83934700	-3.63784600
H	13.43683300	1.69278800	-4.83790600
H	8.18243000	7.06816000	-0.10844200
H	7.17429800	8.19894800	-1.03676800
H	3.49310300	4.86433800	-4.04213000
H	2.23878900	5.83613500	-3.26977200
H	10.83274300	0.61887500	5.87365100
H	11.34497200	1.36857700	4.35589600
H	10.50611700	-3.20585000	-1.62374600
H	10.46557500	-4.51756600	-2.81119300
H	2.61040500	-7.82179100	-1.46018700
H	4.30766900	-7.54770200	-1.88383800
H	-1.19016800	-8.38563900	5.98175000
H	-2.72728700	-7.65943700	5.47716000
H	-6.86824400	-4.82154200	3.62640700
H	-8.31326800	-5.77832600	3.28041300
H	-12.54506200	-0.61165000	-1.56187000
H	-12.57848700	-2.16237000	-2.39744500

H	-8.79464700	3.01658300	0.24404600
H	-10.35079800	2.38791600	0.81351600
H	-9.55906500	7.81962000	1.67719700
H	-9.10956600	9.25144700	2.61862000
H	-8.71652700	1.34711300	-3.72499900
H	-7.78716400	1.09699000	-2.25617100
H	1.06459300	1.54499000	-7.62339700
H	0.01966800	2.96827600	-7.56950400
H	-2.33092200	-5.75653300	-5.80837800
H	-3.92444400	-5.82691600	-6.55416100
H	-3.73155500	-2.74540600	4.56755600
H	-4.69035600	-1.70264200	5.63704200
H	0.86261600	0.47631500	6.75192800
H	0.91782100	-1.28751000	6.58776900
H	-3.55686700	9.81090800	-1.16619100
H	-5.32457300	9.76892000	-1.09839800
H	1.11436900	3.25192700	8.00546000
H	2.02297900	2.54912300	6.65619100
H	1.74442500	-3.33883300	-0.77726400

TS2 (20.7 kcal/mol)

C	-7.21790800	-5.42031900	2.73320100
C	-7.67804100	-4.65909700	1.47728300
C	-6.62207000	-3.69875400	1.01697400
N	-6.86288000	-2.35617200	0.78893600
C	-5.26880100	-3.92851100	0.87408700
C	-5.65035600	-1.82200600	0.55532200
N	-4.64121200	-2.72826000	0.57731200
C	-1.42603400	-8.17896600	4.87692400
C	-0.98803700	-6.71400500	4.72623800
C	-1.37100600	-6.11217100	3.41398800
N	-1.01837400	-6.68132100	2.19605200
C	-2.05635100	-4.97246800	3.07898100
C	-1.48185800	-5.89045800	1.19569300
N	-2.11562900	-4.84646000	1.70155000
C	10.36722200	-4.06592900	-2.04515800
C	8.87109900	-4.30979500	-1.83487100
C	8.35308800	-3.89994600	-0.44552000
C	6.82484700	-4.00074600	-0.39639200
C	8.98095400	-4.72540100	0.68344200
C	13.75424800	2.19666300	-3.85617400
C	13.00261000	1.24330700	-2.91881800
C	11.48354100	1.36290300	-3.03961700
C	10.68542800	0.47288600	-2.07943100
O	11.27410800	-0.35979100	-1.36109600
O	9.40688300	0.66666800	-2.08071600
C	10.79521600	1.30857700	5.07578700
C	9.42220900	0.94864700	4.48505000
C	8.88424800	2.01166200	3.51588400
C	7.45792000	1.76514700	3.01188100
N	7.39707300	0.57518300	2.16075200
C	6.26133400	0.11901700	1.60916900
N	5.05967900	0.56558000	1.98861300
N	6.33726600	-0.84769900	0.68319600
C	3.52476500	-7.55343600	-2.58876700
C	2.89162900	-6.32781600	-3.27263000
C	2.80785900	-5.08063400	-2.39891200
O	1.70109500	-4.59319400	-2.08946700
N	3.96540000	-4.54119700	-1.99209500
C	-3.19310000	-5.19982500	-5.87576300
C	-3.62287800	-5.85530100	-4.55107800
C	-3.01619400	-5.20304900	-3.35022600
N	-1.64441300	-5.04954700	-3.19730600
C	-3.55215800	-4.62015000	-2.22626300
C	-1.40201100	-4.39805200	-2.03269700
N	-2.54256100	-4.11224200	-1.41941600
C	7.72279600	7.31417800	-0.85919400
C	6.62726800	6.26429200	-1.12551400
C	7.16130600	4.86253600	-1.12430300
N	7.29461000	4.13074700	0.04409300
C	7.68117600	4.14280200	-2.18386200
C	7.88938600	3.00356300	-0.32313400
N	8.14200100	2.96029600	-1.65424300
C	2.73925600	4.96437700	-3.17959200
C	2.97045800	4.77934400	-1.67429200
C	3.75228500	3.51194200	-1.28592100
C	3.94821400	3.45326200	0.23190500
C	3.08074600	2.22901900	-1.79116300
C	0.23289900	2.21451900	-7.07529200
C	-0.81030900	2.03594500	-5.95339100
C	-0.54419400	0.83182800	-5.07405700
C	0.34599400	0.91831500	-3.99402300
C	-1.13786700	-0.40960800	-5.33943700

C	0.64046400	-0.19271500	-3.20423200
C	-0.84638900	-1.53104900	-4.55213700
C	0.04470300	-1.42593700	-3.48057300
C	-7.58016700	1.30575500	-3.17927700
C	-7.09933400	0.29354400	-4.23016600
C	-5.85401000	-0.37941900	-3.76527800
N	-5.86494500	-1.19884500	-2.64399800
C	-4.54162100	-0.28492900	-4.14566500
C	-4.62231000	-1.58967500	-2.34568100
N	-3.80420700	-1.04220700	-3.24776800
C	-12.17786200	-1.76112400	-1.34441000
C	-10.75489200	-1.87879300	-0.75098400
C	-9.63288500	-1.53551600	-1.72307200
O	-9.80765500	-1.21571000	-2.87889600
O	-8.35542300	-1.58756200	-1.25205500
C	-9.10866000	2.24338100	1.20465500
C	-8.36637800	0.93353600	1.49281800
C	-6.98934200	1.20277300	2.11215900
C	-9.18705300	0.00903100	2.39938400
C	-8.67166800	8.06159400	2.74004900
C	-7.21331200	8.06035700	2.23989300
C	-6.80043800	6.83782800	1.43980500
C	-5.89823300	5.89755000	1.95359300
C	-7.28314100	6.62347200	0.13958500
C	-5.46851800	4.79891100	1.20343400
C	-6.87316500	5.52981900	-0.62184100
C	-5.95361900	4.61863400	-0.09389400
O	-5.52553200	3.59077100	-0.91018900
C	-3.99655200	-1.99483200	4.63962800
C	-3.76916300	-0.66142300	3.92991200
C	-2.91820700	-0.74778500	2.66949700
O	-2.32029000	-1.85051800	2.43272000
C	1.60212400	-0.55983800	6.21012400
C	1.06374900	-0.62187700	4.76960500
C	1.67254700	-1.73969300	3.95234200
C	3.02218800	-1.68615700	3.57903000
C	0.90597100	-2.83465700	3.53487500
C	3.59388000	-2.69591000	2.80565900
C	1.47457000	-3.85081200	2.76439000
C	2.81957400	-3.78402900	2.39610700
C	1.61909600	3.15577000	7.07508400
C	0.25200000	3.42603100	6.42710900
C	0.26735600	3.40634500	4.91246100
C	1.07533900	4.29732500	4.18623500
C	-0.53576900	2.51190800	4.19469400
C	1.08501900	4.28191600	2.78889100
C	-0.53988100	2.49296200	2.79740800
C	0.27814200	3.37592500	2.09172100
C	-4.34801300	9.16234700	-0.51302500
C	-4.32528700	8.14791100	-1.65745900
C	-3.20265000	7.11664600	-1.50361900
C	-3.14399500	6.16138500	-2.69929400
N	-1.92840800	5.33857400	-2.74089500
C	-1.72025700	4.21325200	-2.03700500
N	-2.71750300	3.59542800	-1.41673500
N	-0.48421900	3.68629500	-2.00470800
Mn	-2.48370700	-2.93396700	0.65307600
O	-0.86071500	1.34397900	-0.44279700
C	-1.17052100	0.18852200	-0.45079000
O	-1.89677200	-0.69176600	-0.80685100
C	0.32250100	-0.78212700	0.87010100
C	0.46068300	-2.04854400	0.22672100
O	-0.42863200	-2.92759900	0.13340300
N	1.66160000	-2.28603900	-0.39762900
C	2.80185300	-1.52021500	-0.20692000
O	3.88824200	-1.92120400	-0.65289700
N	2.68183100	-0.32840700	0.47472800
C	1.50805100	-0.01889400	0.98924100
O	9.01385800	-0.48636000	0.17951400
O	3.31851900	1.94115900	3.77640800
O	-4.04680000	1.49393100	0.01133600
H	8.31116500	-3.74334300	-2.59181400
H	8.63449400	-5.37168800	-2.00615300
H	8.63023500	-2.84956500	-0.29244400
H	6.42394600	-3.67576900	0.56901200
H	6.37010800	-3.37143500	-1.16959700
H	6.49882900	-5.03859400	-0.55519800
H	8.75809600	-5.79319000	0.55953200
H	10.06786700	-4.61249400	0.71180100
H	8.59123700	-4.41428200	1.65887600
H	10.97587200	-4.67842700	-1.37263800
H	13.29038900	1.43540400	-1.87919300
H	13.29438500	0.20626200	-3.11629200
H	11.16294600	2.39914700	-2.86386900

H	11.15466100	1.12714300	-4.06116800
H	13.49888200	3.24216100	-3.64685800
H	8.69813100	0.81447900	5.30047300
H	9.48379800	-0.01856100	3.97179400
H	9.55599600	2.10986400	2.65309400
H	8.88531900	2.99048700	4.01033600
H	7.10797000	2.64568300	2.45538500
H	6.79001500	1.62168700	3.87202900
H	8.24328000	0.27700300	1.65176600
H	7.26981700	-1.05699100	0.31786700
H	5.50329100	-1.14672700	0.18248800
H	4.91543800	1.27040000	2.69940400
H	4.22923500	0.26163800	1.47211700
H	10.74988900	2.25194400	5.63101600
H	3.45925800	-6.07668200	-4.17702200
H	1.86859700	-6.55638100	-3.58118900
H	3.97914800	-3.65759000	-1.47245100
H	4.85273600	-4.93571200	-2.26277800
H	3.51463700	-8.41041200	-3.26663900
H	5.85098700	6.34799300	-0.35529600
H	6.14643100	6.47614500	-2.08935100
H	7.77595000	4.37763400	-3.23335600
H	8.63806700	2.15651400	-2.09602000
H	8.16591900	2.18504200	0.32247700
H	8.49305300	7.27075400	-1.63525900
H	3.51502700	5.65126600	-1.29448200
H	2.00805200	4.77787600	-1.13424000
H	4.74672400	3.58412000	-1.74578700
H	4.55100200	2.58816300	0.51169700
H	4.47273000	4.33863500	0.60065100
H	2.98026800	3.37253800	0.74447000
H	3.63490000	1.34594300	-1.46319000
H	3.01563200	2.19669600	-2.88339500
H	2.06552300	2.13010700	-1.37976800
H	2.12193100	4.16669900	-3.61339900
H	-0.82829600	2.93780200	-5.32859600
H	-1.80741500	1.94777300	-6.40143400
H	0.82407400	1.86790300	-3.77666600
H	-1.81399200	-0.50693400	-6.18613300
H	1.34259000	-0.09994400	-2.38226900
H	-1.29758900	-2.49109700	-4.79078400
H	0.29343100	-2.29602600	-2.88519600

H	0.24985400	1.33988600	-7.73217200
H	-6.89922900	0.78280200	-5.18759200
H	-7.88785100	-0.45030600	-4.38544500
H	-4.07208900	0.25671100	-4.94904700
H	-2.79297400	-1.15879900	-3.25836400
H	-4.33941200	-2.20403000	-1.50512200
H	-6.81506000	2.05457800	-2.95155400
H	-10.63980400	-1.22725100	0.12403800
H	-10.56759500	-2.89524600	-0.37949100
H	-12.91700200	-2.02694500	-0.58516200
H	-8.21022100	0.41205200	0.53837900
H	-6.47477700	0.26207800	2.33090800
H	-6.35293400	1.78857800	1.44156700
H	-7.08898400	1.76160700	3.05040200
H	-8.69643800	-0.96351300	2.50412100
H	-9.30268600	0.44697600	3.39814300
H	-10.19489300	-0.15406800	1.99995600
H	-9.30045800	2.79284700	2.13381000
H	-6.54058600	8.16120600	3.09889900
H	-7.05266600	8.95341600	1.62255400
H	-5.50762900	6.03046300	2.95891400
H	-7.98895200	7.32943600	-0.29035900
H	-4.75715300	4.09186300	1.62084300
H	-7.25515700	5.36918500	-1.62495100
H	-5.16002000	2.83921700	-0.38800200
H	-8.85906700	7.21013700	3.40152600
H	1.26521100	0.33528600	4.27656900
H	-0.02543000	-0.74321500	4.78935400
H	3.61962400	-0.82488200	3.85867700
H	-0.15281000	-2.86990300	3.77258300
H	4.63258000	-2.61715900	2.49925200
H	0.85574600	-4.67638200	2.42961800
H	3.25989500	-4.56072200	1.77720400
H	2.67688000	-0.34929200	6.22026200
H	-0.11345200	4.40241200	6.77027700
H	-0.47250900	2.68606500	6.78396600
H	-1.17285900	1.81817900	4.73660200
H	1.69420900	5.01643000	4.71636900
H	-1.18481700	1.79236700	2.27693400
H	1.71698200	4.97858100	2.24614100
H	0.28388400	3.36259400	1.00677600
H	2.35363200	3.91609600	6.79082100

H	-4.20038200	8.67377900	-2.61369400
H	-5.28851800	7.62801300	-1.70424400
H	-2.23750800	7.63289900	-1.41830300
H	-3.34246700	6.54324100	-0.57941800
H	-3.15901400	6.72600200	-3.63594000
H	-4.02084900	5.50710100	-2.72623500
H	-1.11213900	5.75855400	-3.16091100
H	-2.56542600	2.75993200	-0.85727300
H	-3.69025400	3.89257900	-1.47695800
H	0.32530900	4.21920500	-2.28401400
H	-0.33626300	2.81104500	-1.51579600
H	-4.51388500	8.66069800	0.44594300
H	-6.32193800	-6.01373100	2.52597800
H	-8.59116500	-4.09424200	1.69463900
H	-7.93505100	-5.37929100	0.68848300
H	-8.12615700	-1.87252800	-0.30343000
H	-4.70069200	-4.83935100	0.99442400
H	-5.49031600	-0.76748200	0.37381200
H	-0.95156600	-8.82401200	4.12895300
H	-1.43963900	-6.10474000	5.51457200
H	0.09869900	-6.63365300	4.86231900
H	-0.50586600	-7.54235800	2.07792600
H	-2.48964000	-4.22169400	3.71912900
H	-1.33361900	-6.08953000	0.14644500
H	-3.46132700	-4.13856400	-5.88854300
H	-3.36258500	-6.92182300	-4.57095000
H	-4.71092200	-5.80875000	-4.44663500
H	-0.92410600	-5.37612000	-3.82462000
H	-4.58932600	-4.55181600	-1.93266000
H	-0.41261800	-4.18602300	-1.66867000
H	-4.47210800	-2.71366000	3.96552100
H	-3.28262000	0.06241200	4.59710600
H	-4.71930900	-0.19840100	3.64275700
H	1.47419900	0.90742600	1.55888000
H	9.93904800	-0.78899500	0.08128400
H	8.96496100	-0.01252700	-0.71910600
H	3.21257000	1.92614200	4.73524300
H	2.73468900	2.65793000	3.48562300
H	-3.69261500	1.11985100	0.86664600
H	-4.01622800	0.73942900	-0.59010400
O	-2.81535800	0.27122100	1.93680600
H	-0.46350700	-0.67648000	1.60786800
H	-6.72313200	-1.43431500	-2.11498300
H	14.83860900	2.09087500	-3.74790000
H	13.50412500	2.00236000	-4.90593800
H	8.20681200	7.11828400	0.10206100
H	7.31035600	8.32906900	-0.83907700
H	3.69117200	4.94963300	-3.71947700
H	2.25591500	5.92404300	-3.39864400
H	11.14564900	0.53083100	5.76063700
H	11.54570800	1.42306500	4.28638500
H	10.62326700	-3.01485500	-1.86820200
H	10.66429600	-4.31427600	-3.06926600
H	2.97333900	-7.82594600	-1.68399300
H	4.56444500	-7.36761600	-2.30274000
H	-1.14572800	-8.56475600	5.86053300
H	-2.50954500	-8.27702400	4.76505500
H	-6.97492700	-4.71976600	3.53760400
H	-7.99494500	-6.10276900	3.09225900
H	-12.37130600	-0.74068700	-1.68286000
H	-12.29668900	-2.42621100	-2.20240200
H	-8.52405400	2.89900500	0.54940600
H	-10.07461000	2.05724200	0.72276200
H	-9.37619500	7.99499400	1.90488000
H	-8.88695000	8.98064100	3.29261900
H	-8.47589900	1.81900500	-3.53730800
H	-7.84642000	0.79758100	-2.24991400
H	1.23685400	2.33575200	-6.65724700
H	0.00397800	3.09541000	-7.68191500
H	-2.11160200	-5.27476600	-6.03256700
H	-3.68066200	-5.68636100	-6.72439900
H	-3.04853200	-2.42074900	4.97927100
H	-4.64319400	-1.86423200	5.51240800
H	1.09297600	0.21716900	6.78717300
H	1.45382600	-1.51399400	6.72517200
H	-3.40329800	9.71297300	-0.44945100
H	-5.15144200	9.89196600	-0.65017800
H	1.54192400	3.16412400	8.16601400
H	2.00893700	2.17779600	6.77766500
H	1.74804800	-3.17392000	-0.91903000

E:P (2.8 kcal/mol)

N	-6.64519700	-2.34736500	0.60591500
N	-4.42210100	-2.72786100	0.71046900

N	-0.86321300	-6.41695500	2.79960100
N	-1.68703300	-4.50813500	2.09345300
N	7.60366800	0.77784400	1.94010800
N	5.26301400	0.68665700	2.05525100
N	6.43225300	-0.82206400	0.75352600
N	3.77003200	-4.89009600	-1.03310300
N	-1.38285000	-5.33889000	-2.74603300
N	-2.31906900	-4.38525200	-0.99900400
N	6.47961300	3.90474600	-0.59060400
N	8.17477100	2.93127600	-1.65729100
N	-6.00120700	-1.72974900	-3.07275800
N	-3.99668400	-1.72305100	-3.86485200
N	-2.54530000	4.30246800	-0.92535500
N	-3.60171100	2.45007200	-1.86308100
N	-1.86173700	2.13099800	-0.38085400
N	1.61263700	-1.97601000	-0.31677300
N	2.85175100	-0.29063700	0.77760100
C	-6.95519200	-5.05313300	3.29235700
C	-7.51688300	-4.09202500	2.23725700
C	-6.42635800	-3.39505200	1.48323900
C	-5.06644900	-3.62742200	1.54160600
C	-5.41494400	-1.98545100	0.18422000
C	-1.12800500	-7.57676600	5.62222100
C	-0.69592600	-6.13171400	5.31002200
C	-1.06840500	-5.65342500	3.94059400
C	-1.58351700	-4.46803400	3.47538300
C	-1.24796800	-5.69961900	1.71579300
C	10.55811700	-4.18601300	-1.85270500
C	9.08775600	-4.57570300	-1.68730800
C	8.33159300	-3.74944700	-0.63406400
C	6.85306300	-4.15080000	-0.60365500
C	8.94488100	-3.86241100	0.76583000
C	13.90644400	1.86062000	-4.34045100
C	13.14660300	0.94662500	-3.36753500
C	11.66329800	1.29819800	-3.24512600
C	10.86433900	0.43873400	-2.25430500
C	11.07711900	1.88582300	4.67652700
C	9.69981800	1.44929600	4.15148200
C	9.15563100	2.35988400	3.04053500
C	7.71079600	2.06631600	2.62208700
C	6.42898300	0.23357300	1.58255400
C	3.71329000	-7.71292900	-1.93801400

C	2.63273700	-6.68793100	-2.30987400
C	2.68841800	-5.27791400	-1.71710600
C	-3.05454500	-5.70960800	-5.35159000
C	-3.31427700	-6.39255200	-3.99553600
C	-2.73579300	-5.63646600	-2.84247400
C	-3.29578200	-5.04382600	-1.73273300
C	-1.17694000	-4.59099600	-1.63436600
C	7.90910200	7.25558100	-1.79337500
C	6.97338000	6.34467800	-0.99197600
C	7.28814900	4.88507700	-1.14100700
C	8.34282400	4.28922000	-1.80102200
C	7.04710500	2.75144200	-0.92855200
C	2.89715500	4.67898200	-3.79074600
C	3.12410600	4.60993500	-2.28009100
C	2.86653800	3.23023500	-1.65444400
C	3.24424100	3.24685400	-0.16932800
C	1.42059900	2.75009500	-1.84802600
C	0.34026700	1.54521500	-7.34905700
C	-1.06512300	1.24180500	-6.78795200
C	-1.03734000	0.30524700	-5.59803900
C	-1.03985400	0.79903200	-4.28321400
C	-0.95267200	-1.08245500	-5.78217500
C	-0.93196000	-0.05962700	-3.18838200
C	-0.83801500	-1.94862300	-4.68912900
C	-0.82066400	-1.44050000	-3.38514900
C	-7.41407400	1.03515000	-3.26768300
C	-7.20718500	0.01341000	-4.39567500
C	-6.00071400	-0.81550600	-4.11707000
C	-4.72452800	-0.81363900	-4.61578300
C	-4.78507500	-2.26249100	-2.93031600
C	-11.97993400	-1.82937000	-1.06425800
C	-10.52015100	-2.10974200	-0.65055700
C	-9.48979300	-1.75710000	-1.71446000
C	-8.88118700	2.41435900	1.01935900
C	-8.83208000	0.98133500	1.56600200
C	-7.85498100	0.86292800	2.74490700
C	-10.22860300	0.50558300	1.98389000
C	-8.43406600	8.35878000	1.94722700
C	-6.97354300	8.28109100	1.47053900
C	-6.66606400	6.96256300	0.79910900
C	-6.10538800	5.89607500	1.51240900
C	-7.00578500	6.74526700	-0.54342500

C	-5.89526200	4.65098200	0.91864700
C	-6.81189300	5.50744200	-1.15155000
C	-6.26376900	4.45517400	-0.41510000
C	-3.71379200	-1.44944800	4.79276600
C	-3.84345600	-0.34895400	3.72778000
C	-2.98298000	-0.52949300	2.47463400
C	1.90558700	0.14046600	6.12975200
C	1.53981500	0.00461900	4.64386500
C	2.07089800	-1.25446200	3.99209800
C	3.45114200	-1.46782100	3.87674900
C	1.20154500	-2.20145100	3.43768800
C	3.95284300	-2.58536800	3.21190200
C	1.69977400	-3.32573800	2.77439100
C	3.07629300	-3.52036600	2.65731500
C	1.92776600	3.92420600	6.61073500
C	0.46095300	3.94701100	6.16446400
C	0.29442900	3.95999800	4.66202300
C	0.94945000	4.92035300	3.87282400
C	-0.51855000	3.01825200	4.02122100
C	0.80780000	4.92380700	2.48589900
C	-0.68316900	3.02510600	2.63557800
C	-0.00720600	3.97396900	1.86565200
C	-4.15846800	9.12400800	-1.46331400
C	-3.89509600	7.73570800	-2.08277400
C	-3.31979200	6.68242700	-1.11223600
C	-3.14151600	5.32627000	-1.81316900
C	-2.67795700	2.97434000	-1.03898100
C	0.43145300	-0.27591600	0.81347700
C	0.39892300	-1.46738600	0.07005500
C	2.84927300	-1.45304500	0.06969600
C	1.67923100	0.24170900	1.13017200
O	11.41738300	-0.47600800	-1.61854700
O	9.61181700	0.75034500	-2.14909600
O	1.71474600	-4.51879800	-1.89930500
O	-9.77485700	-1.43966200	-2.85098300
O	-8.18311200	-1.82938900	-1.36125500
O	-6.10282500	3.24481100	-1.04390700
O	-2.20776700	-1.52904400	2.42615000
O	-0.65488000	-2.10967600	-0.28648400
O	3.88931000	-2.06692700	-0.24803000
O	9.08656200	-0.51233800	0.00695300
O	3.37076100	2.64595100	3.04377300

O	-5.33515500	1.17844500	0.42214400
O	-3.09397200	0.32976000	1.53982700
H	8.57204500	-4.45530200	-2.64988400
H	9.01052600	-5.64360100	-1.43143600
H	8.39903200	-2.69690700	-0.93562600
H	6.28613000	-3.56368000	0.12605000
H	6.38721200	-4.00991400	-1.58528100
H	6.75797000	-5.21161900	-0.32535200
H	8.93209500	-4.90203200	1.11832100
H	9.98195400	-3.51754700	0.78064000
H	8.38500600	-3.25412100	1.48405500
H	11.14907400	-4.46526100	-0.97473600
H	13.60378800	0.99287600	-2.37301900
H	13.23854000	-0.09867700	-3.68267800
H	11.54204000	2.34635000	-2.93598200
H	11.16285300	1.22659100	-4.22011600
H	13.85556800	2.90877300	-4.02254800
H	8.98176900	1.44005700	4.98323500
H	9.75228000	0.41738500	3.78438000
H	9.80259600	2.30687300	2.15512400
H	9.18746900	3.40318600	3.37795400
H	7.33943600	2.86685100	1.96715900
H	7.08126200	2.04643700	3.52196000
H	8.40215800	0.44361800	1.37576600
H	7.32726700	-1.04815600	0.31131900
H	5.55482700	-1.21010500	0.40465200
H	5.19855200	1.50090100	2.64567200
H	4.38629200	0.31310400	1.66201900
H	11.03728800	2.89959400	5.09027900
H	2.61441600	-6.54343200	-3.39752700
H	1.63966300	-7.07060700	-2.04640000
H	3.85230600	-3.90691900	-0.73704800
H	4.58723800	-5.47569100	-0.96248200
H	3.50692800	-8.66870800	-2.42573800
H	7.01152400	6.62002200	0.07120100
H	5.93378700	6.51807400	-1.30199400
H	9.17657000	4.70220300	-2.34661500
H	8.80976700	2.16746400	-1.98750800
H	6.66980000	1.76893700	-0.68309700
H	7.86477500	7.01979500	-2.86160600
H	4.16044600	4.88605700	-2.05627600
H	2.48760000	5.35276800	-1.77441600

H	3.53062100	2.51050600	-2.15312400
H	3.18537800	2.23568400	0.24120500
H	4.26864300	3.61677700	-0.04546000
H	2.55240700	3.89594700	0.38632400
H	1.24541900	1.80517300	-1.32248800
H	1.17010600	2.59243400	-2.90152700
H	0.71556900	3.49837800	-1.45340200
H	1.85232100	4.48720200	-4.06128800
H	-1.54915900	2.18179900	-6.49902000
H	-1.67958900	0.80334300	-7.58350400
H	-1.11329100	1.87108600	-4.12002500
H	-0.95147600	-1.48511600	-6.79183300
H	-0.90963500	0.34190500	-2.18039800
H	-0.74499100	-3.01857700	-4.85499300
H	-0.71362900	-2.09080500	-2.52465000
H	0.83752100	0.62626600	-7.67340700
H	-7.08372000	0.51216700	-5.36088400
H	-8.08924800	-0.63208000	-4.45163600
H	-4.26986400	-0.25137900	-5.41409400
H	-2.99308500	-1.89277500	-3.95940100
H	-4.47684700	-2.97401700	-2.18302900
H	-6.57569500	1.73235600	-3.19294400
H	-10.26014200	-1.57429900	0.26841600
H	-10.38100300	-3.17427900	-0.41656700
H	-12.65923800	-2.14087700	-0.26718400
H	-8.47605800	0.31963700	0.76002500
H	-7.73029000	-0.18112100	3.05259100
H	-6.86853700	1.26627600	2.49404300
H	-8.22364800	1.42761100	3.60893500
H	-10.20635400	-0.52047500	2.36810200
H	-10.62826600	1.14508600	2.77946700
H	-10.93374800	0.54239400	1.14681600
H	-9.19473900	3.11841600	1.79866300
H	-6.30094600	8.42570100	2.32341900
H	-6.77777100	9.10608800	0.77525800
H	-5.83324600	6.03787700	2.55498800
H	-7.44216600	7.55691100	-1.11948700
H	-5.46861300	3.82868000	1.48488800
H	-7.09941400	5.33496100	-2.18372400
H	-5.87150100	2.53418900	-0.38873400
H	-8.65000500	7.56349500	2.66751700
H	1.93391500	0.87380700	4.10454000

H	0.44997600	0.03110100	4.52780500
H	4.14169900	-0.72845800	4.27008600
H	0.12930200	-2.03382100	3.48123900
H	5.02540800	-2.70871500	3.09785800
H	1.00718600	-4.03219200	2.32759900
H	3.46448200	-4.37618100	2.11295800
H	2.98951600	0.20062700	6.26645200
H	-0.03220000	4.83044900	6.59069400
H	-0.06400900	3.07677000	6.57237200
H	-1.03056600	2.26757700	4.61628900
H	1.57301400	5.67202200	4.34741200
H	-1.34344400	2.29008400	2.18647200
H	1.33316700	5.66259600	1.88858600
H	-0.06663800	3.98218400	0.78057100
H	2.45447300	4.83386400	6.30774000
H	-3.21127700	7.84578700	-2.93458600
H	-4.83674100	7.34695200	-2.48824600
H	-2.35118700	7.02763000	-0.72454900
H	-3.99199500	6.55634400	-0.25777500
H	-2.50337100	5.43202400	-2.69885200
H	-4.11937200	4.97490300	-2.14370700
H	-1.95827700	4.63102200	-0.16943900
H	-3.63319000	1.44037500	-1.88393600
H	-4.52284900	2.90084300	-1.88623500
H	-0.99201600	2.46916100	0.00062100
H	-2.23691600	1.28272500	0.06054800
H	-4.86816700	9.05035300	-0.63409700
H	-6.35221700	-5.83968100	2.82730100
H	-8.15827000	-3.34320800	2.72013000
H	-8.16943800	-4.64178800	1.54427600
H	-7.81721700	-2.05642700	-0.39980000
H	-4.51004600	-4.35013100	2.11946700
H	-5.23550100	-1.14701600	-0.47207900
H	-0.63618300	-8.30149300	4.96348000
H	-1.14457300	-5.44417400	6.03307000
H	0.39088800	-6.03931800	5.43809700
H	-0.49796700	-7.35736900	2.78319600
H	-1.86375600	-3.58121500	4.01962300
H	-1.20939300	-6.06277300	0.70176400
H	-3.46289100	-4.69404500	-5.35962500
H	-2.91093000	-7.41319200	-4.01771600
H	-4.39081600	-6.49287200	-3.82792900

H	-0.64271500	-5.61593700	-3.37515700
H	-4.32358400	-5.06898000	-1.39918700
H	-0.20385900	-4.23682500	-1.34779800
H	-3.99951800	-2.41799500	4.37352000
H	-3.60564200	0.63699500	4.14693800
H	-4.88239900	-0.27368100	3.38266800
H	1.75004500	1.14676400	1.72988200
H	9.96473500	-0.92899800	-0.03103600
H	9.14882900	0.00997200	-0.86951500
H	2.73744500	3.21547800	3.50120700
H	3.44313800	3.03008600	2.15924500
H	-4.53257600	0.93500600	0.96272200
H	-6.03117700	0.57518400	0.71287100
H	-0.48901800	0.12892700	1.20496600
H	-6.82956700	-1.89041000	-2.45833400
H	14.96362400	1.58302900	-4.40444600
H	13.48487300	1.80409300	-5.35103000
H	8.94790000	7.13521800	-1.46885300
H	7.63930500	8.30817100	-1.66661200
H	3.51518300	3.93867900	-4.31112700
H	3.16210700	5.66489100	-4.18536500
H	11.42973400	1.21393700	5.46467300
H	11.82387500	1.88294600	3.87524200
H	10.67542900	-3.10533800	-1.99384800
H	11.00330900	-4.69071900	-2.71629100
H	3.74813600	-7.88779400	-0.85802300
H	4.70726800	-7.38937900	-2.26418400
H	-0.85465500	-7.83982700	6.64691900
H	-2.20949100	-7.69889800	5.51279400
H	-6.32032500	-4.51872400	4.00588500
H	-7.76095800	-5.53726000	3.85063200
H	-12.12995800	-0.76364000	-1.25477600
H	-12.23328500	-2.36954200	-1.97869900
H	-7.90611200	2.73532800	0.65533500
H	-9.59003300	2.49771500	0.18887300
H	-9.12408300	8.24061900	1.10595700
H	-8.64130900	9.32053300	2.42619300
H	-8.32221100	1.61615600	-3.44545900
H	-7.52991200	0.53282000	-2.30450600
H	0.97023500	2.01419000	-6.58716200
H	0.27493900	2.22123200	-8.20611400
H	-1.98323300	-5.64093800	-5.56831600
H	-3.51967900	-6.27320600	-6.16429000
H	-2.68488600	-1.52319800	5.15574600
H	-4.36585200	-1.23484800	5.64396000
H	1.46252500	1.04186400	6.56320700
H	1.54818300	-0.72118500	6.70296500
H	-3.23571300	9.57105400	-1.07906300
H	-4.57592900	9.80218400	-2.21213200
H	2.00085500	3.85084800	7.69950600
H	2.45898500	3.07142000	6.17835600
H	1.63105300	-2.83870100	-0.87325700
Mn	-2.27553000	-2.84244200	0.75403500

The intermediates and transition states along the reaction pathway proposed for IDCase with Zn instead of Mn.

E:S-Zn (0.0 kcal/mol)

C	-7.51988100	-5.10085000	2.89778400
C	-7.95829000	-3.94704900	1.98741600
C	-6.81100400	-3.30007100	1.26907200
N	-6.97432400	-2.61519100	0.08243900
C	-5.47067600	-3.27328800	1.60589000
C	-5.74163100	-2.20645400	-0.25445200
N	-4.77885900	-2.57078200	0.63161200
C	-1.80071300	-7.88154500	5.20251600
C	-1.17483700	-6.55401900	4.72598000
C	-1.80952300	-5.90816200	3.52511200
N	-2.31179500	-6.61993000	2.44587100
C	-1.93850800	-4.58887900	3.15239000
C	-2.71144500	-5.74273500	1.48834800
N	-2.49197200	-4.50042600	1.88598400
C	10.10749000	-4.39440800	-1.86509000
C	8.61260900	-4.51723200	-1.56049100
C	8.20801200	-4.02366300	-0.16078900
C	6.68237200	-4.01865300	-0.02063300
C	8.84743300	-4.85015300	0.96080800
C	13.64515700	1.68965500	-3.97255800
C	12.86984200	0.79685400	-2.99589200
C	11.35431900	0.91176300	-3.15809600
C	10.53183800	0.10344200	-2.14754200
O	11.10111100	-0.67078100	-1.35233000

O	9.25486000	0.30509100	-2.18775500
C	10.63740400	1.31938700	4.97962500
C	9.22656600	1.07476600	4.42332700
C	8.80725100	2.10508600	3.36548500
C	7.37807000	1.93981700	2.83814900
N	7.24120300	0.67291500	2.11697200
C	6.08275600	0.22199700	1.60436900
N	4.89771500	0.70481200	1.99487300
N	6.12888100	-0.74663000	0.68490800
C	3.18744500	-7.74601300	-2.26339900
C	2.83800300	-6.43912400	-2.99989700
C	2.72518600	-5.24819500	-2.05729100
O	1.62145200	-4.90139200	-1.58983000
N	3.86836800	-4.62105400	-1.74293500
C	-3.46322700	-5.40558200	-5.69377600
C	-4.04397500	-5.83364400	-4.33540800
C	-3.36207800	-5.18141000	-3.17604500
N	-1.98920100	-5.25786200	-2.98165100
C	-3.81588200	-4.41067300	-2.13226400
C	-1.65780700	-4.55692400	-1.87009200
N	-2.74909600	-4.02352300	-1.33370200
C	7.72483500	7.08907300	-1.26222500
C	6.64206700	6.00655100	-1.41835300
C	7.20833100	4.61985100	-1.35935000
N	7.55704800	4.02653100	-0.15673100
C	7.55456200	3.78660500	-2.40517700
C	8.10714400	2.86715300	-0.49287300
N	8.12747700	2.67359000	-1.83407000
C	2.69588200	4.74179900	-3.48389800
C	3.20900900	4.22626000	-2.13271400
C	3.81909200	2.81291800	-2.14856700
C	4.37242000	2.45325500	-0.76701400
C	2.82893600	1.74337400	-2.62257900
C	0.13820800	1.85793000	-7.24850300
C	0.07729600	1.93833100	-5.71257500
C	0.28139700	0.62686400	-4.97500000
C	1.33955200	-0.23352300	-5.30110900
C	-0.54872800	0.26797600	-3.90335600
C	1.56567000	-1.40584100	-4.57931800
C	-0.32715600	-0.90718900	-3.17827200
C	0.73391600	-1.75042400	-3.51237800
C	-7.70659700	1.32665100	-3.34587900

C	-7.04984500	0.15041500	-4.06190700
C	-5.70224800	-0.15182400	-3.49936500
N	-4.91504600	-1.21495100	-3.93730900
C	-4.99011000	0.42000400	-2.47785900
C	-3.78920000	-1.29314300	-3.21020900
N	-3.81889000	-0.30202000	-2.32498900
C	-12.38009400	-1.53730600	-1.37977200
C	-10.92116300	-1.88724700	-1.02683400
C	-9.90202800	-1.35784800	-2.03258800
O	-10.22261600	-0.75501700	-3.03966900
O	-8.59804700	-1.57652100	-1.79174100
C	-9.22690500	2.51814100	0.97904300
C	-8.83469600	1.06156500	0.72483600
C	-7.31787600	0.94493700	0.58187500
C	-9.34585500	0.13111300	1.83031800
C	-8.65935100	8.39439600	2.22296400
C	-7.13891600	8.24888600	2.01928900
C	-6.69442400	6.94589800	1.38056200
C	-6.04122800	5.95334600	2.12260900
C	-6.89638800	6.70188300	0.01381700
C	-5.57418200	4.77671600	1.53074900
C	-6.44489300	5.53106000	-0.59409900
C	-5.76552500	4.57360100	0.16341400
O	-5.26202100	3.46979700	-0.49613900
C	-4.22608800	-1.65915200	4.64422900
C	-3.14444400	-0.58047800	4.62769300
C	-2.48027800	-0.44173400	3.28051200
O	-2.34893800	-1.36348700	2.48265300
C	1.40050900	-0.27706300	6.16618000
C	1.35666400	-0.12182000	4.63522200
C	1.87247900	-1.35738700	3.93447100
C	3.25050500	-1.56443700	3.78724700
C	0.99711800	-2.34257900	3.46302000
C	3.73999200	-2.72325400	3.18575900
C	1.48117400	-3.51368700	2.87682300
C	2.85608800	-3.70710000	2.73712600
C	1.50000400	3.47595200	6.84376000
C	0.39317400	3.68335100	5.80517000
C	0.89240100	3.90476000	4.39277000
C	2.02923500	4.68247500	4.11754900
C	0.20789700	3.33329400	3.31441400
C	2.47768500	4.86257200	2.80503200

C	0.63926200	3.52739500	2.00179400
C	1.78344400	4.28227800	1.74119200
C	-4.30077600	9.23046000	-1.06209900
C	-4.08759100	8.01952800	-1.98040600
C	-3.01613700	7.04124800	-1.47444100
C	-2.83355900	5.85931500	-2.43892900
N	-1.60582400	5.08311400	-2.21075100
C	-1.50183900	3.88249300	-1.62326100
N	-2.55485900	3.21057000	-1.16792200
N	-0.29292700	3.30365400	-1.50912400
Zn	-2.70209400	-2.78552400	0.51467600
O	-1.40453600	1.15878500	0.40751900
C	-1.23161300	-0.08982500	0.12926100
O	-2.21604000	-0.78526700	-0.32664200
C	0.06924500	-0.69118400	0.30247700
C	0.24989700	-2.10014500	0.13368000
O	-0.64007000	-2.98485300	0.17539200
N	1.53751500	-2.49983600	-0.08629300
C	2.65458500	-1.69560100	0.06239800
O	3.77450300	-2.17296500	-0.18450900
N	2.47537800	-0.39970500	0.48735800
C	1.23703700	0.05602000	0.55185400
O	8.84126000	-0.56781500	0.19633200
O	4.00336400	2.16381700	4.34374600
O	-4.26036400	1.23619900	0.65747900
H	8.05261300	-3.93414300	-2.30496200
H	8.28882200	-5.56347500	-1.67816600
H	8.55609700	-2.98769400	-0.06559900
H	6.36031700	-3.62911600	0.95046500
H	6.22324900	-3.38875500	-0.79033000
H	6.27536800	-5.03432300	-0.11889800
H	8.55505900	-5.90568400	0.88557700
H	9.93915100	-4.80281200	0.92904200
H	8.53020600	-4.48624300	1.94430600
H	10.70868200	-5.03187800	-1.20922400
H	13.13426100	1.05219000	-1.96374800
H	13.16348800	-0.25077400	-3.12241700
H	11.03604900	1.95970100	-3.06869000
H	11.04499100	0.60023600	-4.16529200
H	13.39020100	2.74659200	-3.83138000
H	8.49913400	1.09426300	5.24710300
H	9.16899300	0.06782900	3.99328800
H	9.50586700	2.07417900	2.51887700
H	8.88792300	3.11639900	3.78206800
H	7.12584500	2.78147200	2.17871500
H	6.67671200	1.94863800	3.68177200
H	8.06379600	0.31832000	1.60844000
H	7.05153200	-1.03565100	0.35609600
H	5.27885700	-1.20547900	0.35913100
H	4.79286800	1.31670100	2.79804300
H	4.05468100	0.35591200	1.53621100
H	10.71111200	2.30652500	5.44932200
H	3.59099700	-6.23243300	-3.76854500
H	1.87134300	-6.53853800	-3.50098400
H	3.87402600	-3.80495900	-1.12081000
H	4.75176600	-4.92610100	-2.12065400
H	3.22407400	-8.58182200	-2.96662700
H	5.89618100	6.12459500	-0.62169500
H	6.11735400	6.14734400	-2.37191000
H	7.46578000	3.90307400	-3.47481200
H	8.54363500	1.81599700	-2.25788900
H	8.50594000	2.12464100	0.18103500
H	8.45794200	7.01815900	-2.07159700
H	3.96436100	4.92257500	-1.75296300
H	2.39519200	4.25029600	-1.38822900
H	4.66421500	2.83572900	-2.85043300
H	4.83980300	1.46576000	-0.78213400
H	5.12543000	3.17071600	-0.43250700
H	3.56879400	2.41938600	-0.02167400
H	3.29737400	0.75541300	-2.63311400
H	2.44440200	1.93161900	-3.62797700
H	1.97068000	1.66648000	-1.94170300
H	1.85852900	4.14568700	-3.86960700
H	0.84261500	2.65078900	-5.37272100
H	-0.88591500	2.36510500	-5.40783000
H	2.00706300	0.02250900	-6.11821800
H	-1.37116000	0.92504700	-3.62670400
H	2.40187700	-2.04602200	-4.84432600
H	-0.96686300	-1.15950300	-2.33993800
H	0.91660900	-2.65713200	-2.94345200
H	-0.61528000	1.16530500	-7.63604500
H	-6.96208000	0.34593900	-5.13874900
H	-7.69555100	-0.72469900	-3.93067400
H	-5.22572400	1.25631800	-1.84262600

H	-3.15689800	-0.26869700	-1.50339000
H	-3.00984400	-2.02884400	-3.30908500
H	-7.12373700	2.24773200	-3.45229000
H	-10.65324700	-1.49751800	-0.03773800
H	-10.78383700	-2.97495100	-0.95914500
H	-13.05926500	-1.95184600	-0.63020200
H	-9.28780500	0.74675000	-0.22489700
H	-7.02485100	-0.07752200	0.33497200
H	-6.95585500	1.61608700	-0.20114300
H	-6.82327500	1.23246800	1.51811000
H	-9.03908100	-0.90361500	1.64358000
H	-8.93532500	0.42614500	2.80378500
H	-10.43794500	0.16072600	1.90975800
H	-8.77679900	2.88488100	1.90974100
H	-6.63593000	8.35614400	2.98677600
H	-6.78646000	9.08283600	1.39900900
H	-5.87525700	6.10826300	3.18530700
H	-7.40906200	7.44586100	-0.59021700
H	-5.05317800	4.02710700	2.12008900
H	-6.61032300	5.34792800	-1.65121400
H	-5.03881000	2.73434700	0.12232400
H	-9.04391200	7.60559200	2.87679800
H	1.96200100	0.74399900	4.35070500
H	0.32922600	0.08518600	4.31381600
H	3.93939300	-0.79828000	4.12760700
H	-0.07401400	-2.17639300	3.53093300
H	4.80998200	-2.85184800	3.05454400
H	0.77994800	-4.25343600	2.50291700
H	3.23830200	-4.60325300	2.25776000
H	2.42932300	-0.42435000	6.50806100
H	-0.22980500	4.53664300	6.10664100
H	-0.27209600	2.81285500	5.80046800
H	-0.67317700	2.73066000	3.51035400
H	2.56594600	5.16017400	4.93205400
H	0.07459900	3.08053500	1.19165900
H	3.36494000	5.45868600	2.61348800
H	2.13710600	4.42087200	0.72568900
H	2.08105200	4.38923400	7.00487100
H	-3.80412800	8.36684500	-2.98297900
H	-5.03524700	7.48102900	-2.09260600
H	-2.05874400	7.56899600	-1.37174600
H	-3.28170000	6.66636900	-0.47903900
H	-2.78589600	6.22484000	-3.47037500
H	-3.68900500	5.18108400	-2.39281500
H	-0.73692900	5.53586800	-2.45714500
H	-2.34701400	2.36089900	-0.63810400
H	-3.48862800	3.60786300	-1.05871600
H	0.53438900	3.67213600	-1.95221100
H	-0.22604400	2.41754700	-1.02270100
H	-4.63365600	8.90986600	-0.06943800
H	-6.99570100	-5.87198800	2.32417900
H	-8.50281700	-3.20103000	2.58374500
H	-8.67465800	-4.31044600	1.24070400
H	-8.28712900	-2.05495600	-0.96054900
H	-4.95731300	-3.71123800	2.44629400
H	-5.54259800	-1.63805800	-1.14765000
H	-1.71646100	-8.66745100	4.44315500
H	-1.19851700	-5.82314100	5.54021300
H	-0.11108000	-6.72112100	4.50930800
H	-2.38582400	-7.62464700	2.39142500
H	-1.65382500	-3.70442400	3.69759800
H	-3.15017400	-6.03339900	0.54668300
H	-3.52592000	-4.32012600	-5.82554500
H	-3.98086300	-6.92570300	-4.24176300
H	-5.10903300	-5.58742200	-4.28432900
H	-1.32914200	-5.75210200	-3.56371400
H	-4.82631400	-4.11200900	-1.89267200
H	-0.64973300	-4.47574700	-1.49377900
H	-5.00427200	-1.45399700	3.90466100
H	-2.34711000	-0.81519000	5.34676600
H	-3.53709000	0.39772600	4.91697200
H	1.13182800	1.10237300	0.83105000
H	9.75659200	-0.90614600	0.12798900
H	8.79475900	-0.20401300	-0.75053600
H	4.52582400	2.50167200	5.08110700
H	3.45582400	2.91317200	4.05705300
H	-3.29695100	1.08310100	0.66956100
H	-4.67462800	0.36414500	0.72232800
O	-2.03003100	0.79332300	3.06298100
H	-1.63798500	0.89178000	2.15950600
H	-5.16752100	-1.85419800	-4.67776200
H	14.72661800	1.58699200	-3.83539800
H	13.41624000	1.43398200	-5.01400600
H	8.25765300	6.95220000	-0.31693700

H	7.29263200	8.09581300	-1.27530300
H	3.48672900	4.69988600	-4.23970800
H	2.36440600	5.78421500	-3.41500000
H	10.90190900	0.56873800	5.73027300
H	11.38710000	1.27459200	4.18246700
H	10.44698800	-3.36012100	-1.73648600
H	10.32314000	-4.69261500	-2.89634200
H	2.43773000	-7.97019400	-1.49905100
H	4.16127200	-7.67376200	-1.77048000
H	-1.27835100	-8.23883400	6.09271600
H	-2.85892100	-7.75884100	5.45121000
H	-6.84097100	-4.74844100	3.68144500
H	-8.38022800	-5.56364400	3.38967800
H	-12.51683000	-0.45401100	-1.41765300
H	-12.64735700	-1.93795100	-2.35995800
H	-8.87979400	3.17014200	0.17010200
H	-10.31243900	2.63571100	1.06500100
H	-9.19345600	8.32541400	1.26989500
H	-8.89567800	9.36182500	2.67522400
H	-8.70685600	1.48762600	-3.74866800
H	-7.81989900	1.09956800	-2.28429000
H	1.11666900	1.51507200	-7.59579900
H	-0.04177500	2.84214300	-7.69023100
H	-2.40973800	-5.68672600	-5.79273100
H	-4.00649500	-5.88074700	-6.51458600
H	-3.80439100	-2.63918600	4.40993900
H	-4.69203100	-1.71127600	5.63141200
H	0.99916000	0.60907400	6.66715400
H	0.81794900	-1.14683600	6.48838700
H	-3.37688600	9.80564200	-0.94000600
H	-5.06153900	9.90022800	-1.47271200
H	1.07674700	3.18827700	7.81023700
H	2.19002000	2.68949100	6.52303200
H	1.66039600	-3.47376000	-0.39957900

TS1-Zn (15.2 kcal/mol)

C	-7.48508400	-5.14455000	2.82523100
C	-7.94028300	-4.05979000	1.84515700
C	-6.79249900	-3.39290700	1.14966800
N	-6.96035300	-2.61705100	0.02233500
C	-5.44556100	-3.42429100	1.46054400
C	-5.72365700	-2.21153900	-0.30720700
N	-4.75665100	-2.66357200	0.52933400
C	-1.75318700	-7.94993600	5.06780900
C	-0.98807200	-6.78582700	4.40802300
C	-1.64963200	-6.14732300	3.22274600
N	-2.05500200	-6.84408800	2.09190200
C	-1.89965400	-4.83069000	2.91942200
C	-2.51352000	-5.95348000	1.17242600
N	-2.42764100	-4.72134000	1.64363500
C	10.13212500	-4.29083600	-1.95128600
C	8.64895300	-4.43539000	-1.60251100
C	8.28030500	-3.94448700	-0.19216600
C	6.76047400	-3.96941000	-0.00039200
C	8.97304300	-4.75249500	0.91107800
C	13.64671200	1.84350200	-3.94863600
C	12.87452900	0.93251200	-2.98623300
C	11.35854800	1.04450700	-3.14650700
C	10.53857200	0.21868400	-2.14795600
O	11.11132800	-0.56314900	-1.36213900
O	9.26105100	0.41444300	-2.18694200
C	10.65494400	1.29388900	4.99969500
C	9.24665100	1.06659800	4.43316600
C	8.82689100	2.13533900	3.41500100
C	7.39921700	1.98100600	2.88220500
N	7.26949100	0.73659400	2.12398200
C	6.10964000	0.26860200	1.63554100
N	4.92403100	0.72071300	2.06020600
N	6.15173900	-0.69597400	0.71074100
C	3.22221700	-7.65673100	-2.40206400
C	2.84407700	-6.30091800	-3.02715200
C	2.74077400	-5.17885600	-2.00168000
O	1.63341800	-4.80906100	-1.56218000
N	3.89450200	-4.63498500	-1.58698900
C	-3.44156000	-5.27332300	-5.77690300
C	-3.94085100	-5.81746800	-4.42892900
C	-3.27099700	-5.15799800	-3.26894700
N	-1.89443000	-5.19470400	-3.08749700
C	-3.73605000	-4.38330800	-2.23426200
C	-1.57110700	-4.46732200	-1.99028900
N	-2.67216500	-3.95503100	-1.45458200
C	7.71354200	7.17135400	-1.12707100
C	6.62964300	6.09180100	-1.29798700
C	7.19865400	4.70542400	-1.27001300

N	7.55690400	4.09008500	-0.08130600
C	7.54413400	3.89534800	-2.33406900
C	8.11275000	2.94152000	-0.44340200
N	8.12730400	2.77482400	-1.78827800
C	2.68793300	4.84978900	-3.38542200
C	3.14314700	4.36239400	-2.00383800
C	3.76874300	2.95649400	-1.98058300
C	4.29404000	2.62542500	-0.58100800
C	2.79644100	1.87117600	-2.45766300
C	0.13437000	2.03036700	-7.19854000
C	0.07938700	2.09296500	-5.65993800
C	0.30230900	0.77584700	-4.93671600
C	1.39091200	-0.04931300	-5.25477400
C	-0.53815800	0.37709200	-3.88724000
C	1.63731900	-1.22563300	-4.54661700
C	-0.29696000	-0.80457800	-3.17916000
C	0.79397600	-1.61205700	-3.50299400
C	-7.70274400	1.39948500	-3.29508900
C	-7.02215300	0.23346500	-4.00507400
C	-5.67070700	-0.05994400	-3.44442700
N	-4.88875400	-1.12154000	-3.89533200
C	-4.94936900	0.50262000	-2.42313400
C	-3.75845300	-1.20866600	-3.17717200
N	-3.77697800	-0.22424200	-2.28494100
C	-12.36360900	-1.51641500	-1.37612500
C	-10.90536100	-1.90111600	-1.06176100
C	-9.89190100	-1.30283000	-2.03330300
O	-10.21975700	-0.65303300	-3.00836300
O	-8.58591300	-1.51754800	-1.80159200
C	-9.21961200	2.50351700	1.05385700
C	-8.85788200	1.04519100	0.76885700
C	-7.33971500	0.89334600	0.68241200
C	-9.43388900	0.09605400	1.82529500
C	-8.66897700	8.35719800	2.40817400
C	-7.14736900	8.15487500	2.25397300
C	-6.70538500	6.84395200	1.62412700
C	-6.05414700	5.85577000	2.37425100
C	-6.88165400	6.59564800	0.25411300
C	-5.56328700	4.68401600	1.79016500
C	-6.40454900	5.43079400	-0.34673000
C	-5.72432900	4.47875800	0.41931900
O	-5.19835300	3.38561500	-0.23899700
C	-4.19942700	-1.72606100	4.63136400
C	-3.41536300	-0.51021200	4.13647400
C	-2.37638400	-0.87977600	3.11599300
O	-2.38736900	-1.92948000	2.48449300
C	1.42500600	-0.35471100	6.17034300
C	1.62465100	-0.16150800	4.65731100
C	2.03185500	-1.44272000	3.96426600
C	3.38405200	-1.72158300	3.72544400
C	1.08107600	-2.39973800	3.58242400
C	3.77679600	-2.91595300	3.12159800
C	1.46814000	-3.60025900	2.98404300
C	2.81920700	-3.86145900	2.75175900
C	1.51384100	3.38509200	6.91866800
C	0.26784300	3.47662700	6.02993200
C	0.54540100	3.64936100	4.55012100
C	1.57599000	4.47498700	4.07123600
C	-0.25183700	2.98415600	3.61187600
C	1.81103200	4.61074300	2.69843600
C	-0.03574700	3.12766700	2.24100700
C	1.00495500	3.93472700	1.77867000
C	-4.31855800	9.26953600	-0.86732900
C	-4.22907500	8.10931200	-1.86356200
C	-3.12486000	7.10151900	-1.51697100
C	-3.01657100	5.98775400	-2.56659900
N	-1.77223200	5.21630600	-2.48194100
C	-1.59900000	4.02896600	-1.87646500
N	-2.60369700	3.35134700	-1.32705600
N	-0.37334200	3.48039100	-1.84926900
Zn	-2.68391600	-2.85648800	0.45760400
O	-1.14073000	1.31793000	-0.28066400
C	-1.14893000	0.07678700	-0.05871800
O	-2.25109800	-0.61120600	-0.17256800
C	0.09862900	-0.59511200	0.30587900
C	0.25471900	-2.01342100	0.16165500
O	-0.64518000	-2.88558300	0.11571900
N	1.54388900	-2.44554100	0.01244100
C	2.67578500	-1.66554600	0.15938900
O	3.78907100	-2.17586100	-0.04024900
N	2.51564300	-0.34585600	0.51818300
C	1.29134200	0.14225600	0.50503000
O	8.85725200	-0.49113500	0.18878200
O	3.99071900	2.39139900	4.23460000

O	-4.25941000	1.03443200	0.92823600
H	8.05767300	-3.86219500	-2.33028600
H	8.33779200	-5.48669200	-1.70909300
H	8.60982300	-2.90127200	-0.11236300
H	6.46560300	-3.57454700	0.97711500
H	6.26163800	-3.35812100	-0.76071900
H	6.37256100	-4.99493900	-0.07078400
H	8.70235500	-5.81459900	0.84822000
H	10.06176100	-4.68061600	0.84427200
H	8.67906400	-4.39221700	1.90309600
H	10.76080400	-4.92688300	-1.32035600
H	13.13811200	1.17243400	-1.95019800
H	13.17202000	-0.11191300	-3.12935200
H	11.03676100	2.08989000	-3.04115000
H	11.05001200	0.74736400	-4.15826800
H	13.38795600	2.89712600	-3.79066800
H	8.51764700	1.05272800	5.25549400
H	9.19265500	0.07674200	3.96478500
H	9.52629700	2.13975100	2.56850400
H	8.90258700	3.13029300	3.87009100
H	7.14236600	2.84105400	2.24871600
H	6.70040700	1.95544500	3.72768300
H	8.09005500	0.39390100	1.60420400
H	7.07298000	-0.98159600	0.37554600
H	5.30336800	-1.17723500	0.41598200
H	4.82520700	1.41317600	2.79604200
H	4.08224000	0.39886700	1.58075600
H	10.72573300	2.26301800	5.50599600
H	3.57904300	-6.02769100	-3.79280800
H	1.86830800	-6.37113500	-3.51503200
H	3.89935400	-3.83788500	-0.94173600
H	4.78039900	-4.93597300	-1.96221200
H	3.24574600	-8.43575400	-3.16826500
H	5.88896300	6.19335200	-0.49447200
H	6.09852800	6.25056200	-2.24509900
H	7.44911900	4.03278000	-3.40066800
H	8.54393900	1.92742700	-2.23041600
H	8.52085200	2.18889500	0.21316400
H	8.44183200	7.11631100	-1.94203300
H	3.87592500	5.07337500	-1.60715500
H	2.29552800	4.38528800	-1.30062400
H	4.63134600	2.97157200	-2.66126100

H	4.74843700	1.63243500	-0.56661300
H	5.05289300	3.34054600	-0.25406800
H	3.47790200	2.62317200	0.15311600
H	3.26605300	0.88416700	-2.42821900
H	2.44466300	2.03150600	-3.48012400
H	1.91747100	1.81442600	-1.80280000
H	1.90622600	4.21343700	-3.82239900
H	0.83978400	2.80924700	-5.31694600
H	-0.88629600	2.50688400	-5.34554900
H	2.06719900	0.23895800	-6.05389500
H	-1.37947200	1.00801500	-3.60976800
H	2.49737600	-1.83638000	-4.80444300
H	-0.94724500	-1.09563800	-2.36167600
H	0.99081600	-2.52211900	-2.94379000
H	-0.61795900	1.33898900	-7.59042700
H	-6.93449100	0.42953600	-5.08186500
H	-7.65452600	-0.65143600	-3.87538300
H	-5.17772500	1.33857800	-1.78269500
H	-3.10967000	-0.19495300	-1.46300800
H	-2.98525300	-1.94961100	-3.28458300
H	-7.14700800	2.33491300	-3.42050700
H	-10.62771300	-1.59317300	-0.04720500
H	-10.77486500	-2.99164200	-1.08227100
H	-13.04135100	-1.98753400	-0.65940000
H	-9.27982000	0.77114100	-0.20713300
H	-7.06684800	-0.13168900	0.41989400
H	-6.92520400	1.57714700	-0.06244700
H	-6.88696700	1.14524600	1.65093300
H	-9.13443700	-0.93872300	1.62590300
H	-9.06469300	0.35925500	2.82409500
H	-10.52770300	0.14131200	1.85449600
H	-8.79743200	2.82799100	2.01282000
H	-6.67562300	8.24334600	3.23895200
H	-6.74612000	8.98050900	1.65210600
H	-5.90462100	6.01129800	3.43930100
H	-7.39204300	7.33312800	-0.35986500
H	-5.04063600	3.94395800	2.39005200
H	-6.55034800	5.24899800	-1.40723800
H	-4.90015600	2.67361700	0.37326400
H	-9.10506500	7.58291300	3.04678500
H	2.39092300	0.60164800	4.49375900
H	0.69551500	0.22104400	4.22354800

H	4.13126900	-0.98398600	3.99859700
H	0.02687500	-2.20627900	3.75857100
H	4.82842100	-3.09987500	2.92603800
H	0.71191600	-4.31732800	2.68115000
H	3.12361900	-4.78469000	2.26776700
H	2.35132900	-0.69411300	6.64403200
H	-0.36076200	4.30798000	6.37775800
H	-0.33689200	2.57185100	6.15764700
H	-1.04940900	2.33450000	3.95671300
H	2.19337000	5.02823500	4.77360200
H	-0.66839300	2.59870100	1.53621500
H	2.61733800	5.24905000	2.34875800
H	1.18297800	4.04275500	0.71333500
H	2.04385900	4.34167100	6.97017900
H	-4.04519800	8.50521800	-2.87132500
H	-5.19212000	7.58779400	-1.90392800
H	-2.16151200	7.62547300	-1.45849800
H	-3.30635300	6.66187700	-0.52874900
H	-3.05203300	6.41892700	-3.57269300
H	-3.86073800	5.29635500	-2.49942600
H	-0.92756800	5.70296900	-2.74683400
H	-2.33929100	2.47977000	-0.85022600
H	-3.51250100	3.74784000	-1.10442900
H	0.38053100	3.83538500	-2.41590600
H	-0.26365500	2.58315200	-1.37640800
H	-4.55095100	8.90070500	0.13749700
H	-6.90127500	-5.91514100	2.31150900
H	-8.54252500	-3.31172900	2.38017600
H	-8.60843000	-4.49324600	1.09026100
H	-8.26739200	-2.02788400	-0.99090600
H	-4.92322200	-3.94321400	2.24844000
H	-5.52861900	-1.57564200	-1.15444400
H	-1.89582300	-8.78718400	4.37549400
H	-0.81723900	-5.99331900	5.14292300
H	0.01023100	-7.13431100	4.11029800
H	-2.02358100	-7.84594200	1.97626300
H	-1.72335100	-3.95731200	3.52374700
H	-2.89436500	-6.23069300	0.20164600
H	-3.60846500	-4.19338700	-5.84947000
H	-3.78311500	-6.90303900	-4.39067400
H	-5.01976200	-5.66274200	-4.33334400
H	-1.22845100	-5.68191200	-3.66889500

H	-4.75128700	-4.10791900	-1.98756400
H	-0.56310600	-4.36358100	-1.61816800
H	-4.73097400	-2.20397200	3.80652600
H	-2.92312000	0.03171600	4.95015500
H	-4.07134100	0.21544000	3.63600500
H	1.20250800	1.20959800	0.69192200
H	9.77697100	-0.81645500	0.11012600
H	8.80064800	-0.11773300	-0.75323500
H	4.48982700	2.96185800	4.83151900
H	3.26596500	2.94697200	3.90453700
H	-3.48333100	0.53523200	0.59927500
H	-4.97403800	0.38181300	0.94524200
O	-1.42536700	0.03633000	2.95532800
H	-0.78447900	-0.28027600	2.27824100
H	-5.14999800	-1.75594400	-4.63698500
H	14.72851900	1.74250300	-3.81299700
H	13.41869700	1.60358500	-4.99402200
H	8.25189400	7.01591300	-0.18781500
H	7.28132200	8.17814400	-1.11775300
H	3.52152900	4.84039200	-4.09522400
H	2.30465600	5.87584500	-3.34436100
H	10.92073300	0.51682900	5.72255900
H	11.40615000	1.28058400	4.20275200
H	10.46302900	-3.25387500	-1.82171200
H	10.32007500	-4.57632600	-2.99151300
H	2.49526800	-7.94708000	-1.63781600
H	4.20836400	-7.61604600	-1.93061800
H	-1.19278500	-8.32785600	5.92630800
H	-2.73970500	-7.62834800	5.41339000
H	-6.85683400	-4.72202600	3.61643400
H	-8.34098100	-5.62760800	3.30487100
H	-12.49514800	-0.43295200	-1.32381900
H	-12.63795700	-1.83338000	-2.38448500
H	-8.82295800	3.17047200	0.28084500
H	-10.30411100	2.64869100	1.10121800
H	-9.17492500	8.31210500	1.43838000
H	-8.88023900	9.33231000	2.85595100
H	-8.71203000	1.52511300	-3.68716500
H	-7.79747600	1.18355800	-2.22891100
H	1.11294100	1.69485100	-7.55268200
H	-0.05153400	3.01890900	-7.62786500
H	-2.36943000	-5.44805200	-5.91264100

H	-3.96136900	-5.75485500	-6.60930200
H	-3.53360600	-2.46627100	5.08390400
H	-4.92997800	-1.42089200	5.38457600
H	1.12028400	0.57913200	6.65056300
H	0.65652000	-1.10883400	6.37227900
H	-3.37424100	9.82154800	-0.81398700
H	-5.10325100	9.97442800	-1.15636500
H	1.23985600	3.11282700	7.94168200
H	2.21389200	2.63576700	6.53694900
H	1.65221100	-3.42001600	-0.30118400

Int-Zn (11.2 kcal/mol)

C	-7.46717800	-5.18695500	2.84876200
C	-7.94285000	-4.07050200	1.91092300
C	-6.80790300	-3.37774100	1.21737800
N	-6.97825600	-2.61880900	0.07747600
C	-5.46401200	-3.37989200	1.54007500
C	-5.74402400	-2.19944300	-0.24816000
N	-4.77833100	-2.62732000	0.60144100
C	-1.73147800	-7.95063600	5.13270500
C	-0.98220100	-6.75400900	4.51564700
C	-1.64267400	-6.11094500	3.33271600
N	-2.05994700	-6.81325200	2.20906800
C	-1.89916200	-4.79530200	3.02917500
C	-2.53446300	-5.92692700	1.29313200
N	-2.44479600	-4.69509900	1.76067200
C	10.17282300	-4.23355300	-1.82336900
C	8.65826100	-4.34659600	-1.63159300
C	8.16013200	-3.90266500	-0.24498700
C	6.62822500	-3.86767600	-0.21707700
C	8.69561800	-4.79192500	0.88301000
C	13.66079600	1.91799600	-3.81443300
C	12.89120900	0.98635300	-2.87059000
C	11.37589400	1.08819900	-3.04187200
C	10.55573600	0.24905000	-2.05669000
O	11.12347300	-0.54661200	-1.28256700
O	9.27713200	0.44987600	-2.09141600
C	10.61840000	1.37104900	5.11694200
C	9.22463900	1.11253400	4.52876700
C	8.80334200	2.16912600	3.49909100
C	7.38471500	1.99734700	2.95054200
N	7.27387000	0.75213500	2.18619600
C	6.12481100	0.31014900	1.65758600
N	4.93879500	0.80078400	2.03167000
N	6.17557300	-0.67423200	0.74855400
C	3.28711100	-7.64396200	-2.30765600
C	2.90428800	-6.41121800	-3.15149400
C	2.82714400	-5.12935700	-2.33476800
O	1.75340600	-4.75948600	-1.81542500
N	3.96281900	-4.42888900	-2.19465400
C	-3.37096600	-5.31081500	-5.72847800
C	-3.98038200	-5.65368000	-4.35743100
C	-3.27258300	-5.01273000	-3.20441000
N	-1.89454700	-5.07454200	-3.04782700
C	-3.71085400	-4.29859500	-2.11281100
C	-1.54929200	-4.42018400	-1.91041200
N	-2.63160500	-3.92944400	-1.32143200
C	7.67726500	7.21509400	-1.04179000
C	6.61202600	6.11428600	-1.19259700
C	7.20490600	4.73769900	-1.16833900
N	7.58740200	4.12903800	0.01656400
C	7.54353600	3.92857500	-2.23528200
C	8.14992200	2.98558700	-0.35074100
N	8.14660400	2.81566900	-1.69552800
C	2.68062000	4.85687500	-3.32415400
C	3.13885100	4.33978800	-1.95357100
C	3.77283700	2.93707100	-1.96491700
C	4.36280700	2.60392600	-0.59337500
C	2.78626900	1.84291600	-2.39053900
C	0.16705500	2.01145100	-7.14715000
C	0.29041300	2.04891600	-5.61259900
C	0.50037900	0.69873900	-4.95336500
C	1.59073000	-0.10914100	-5.31027600
C	-0.35232300	0.24284100	-3.93923700
C	1.82080700	-1.33045500	-4.67882700
C	-0.12720600	-0.98378300	-3.30596800
C	0.95937300	-1.77735000	-3.67399000
C	-7.68884700	1.34084300	-3.28882700
C	-7.00131100	0.17687000	-3.99446700
C	-5.64598200	-0.09297800	-3.43344000
N	-4.86156500	-1.16609000	-3.84999000
C	-4.92587100	0.50827700	-2.43315800
C	-3.73119800	-1.22369500	-3.12887800
N	-3.75415000	-0.21133600	-2.26945800

C	-12.34299600	-1.59970400	-1.39098600	N	-1.83834200	5.12176400	-2.32805200
C	-10.88020100	-1.91463000	-1.02515500	C	-1.57842600	3.89735700	-1.84064800
C	-9.86679200	-1.36512700	-2.02578300	N	-2.51759900	3.13335200	-1.29292500
O	-10.19581700	-0.76544600	-3.03294900	N	-0.32934200	3.40946500	-1.92822800
O	-8.56294400	-1.55912400	-1.77603300	Zn	-2.69352400	-2.79891500	0.65614100
C	-9.23919500	2.44603100	1.04832900	O	-0.79345700	1.03480700	-0.47929800
C	-8.74402300	1.01759100	0.80785800	C	-0.99191000	-0.14288900	-0.12745600
C	-7.21555200	0.98624900	0.75136300	O	-1.98984800	-0.86307400	-0.43250500
C	-9.26536300	0.04561500	1.87193000	C	0.08513000	-0.82668400	0.77434000
C	-8.73353000	8.30610600	2.39153600	C	0.35935600	-2.21078500	0.28385800
C	-7.34972400	8.35243000	1.71196400	O	-0.47222800	-3.11508700	0.31918500
C	-6.81708300	7.02132400	1.21073900	N	1.58480500	-2.42372200	-0.28134100
C	-5.73260300	6.38833500	1.83285300	C	2.66563500	-1.57234700	-0.15059800
C	-7.36083000	6.40030200	0.07575300	O	3.75347800	-1.87485300	-0.62974800
C	-5.18672000	5.20081600	1.33615600	N	2.50288800	-0.35867900	0.54503200
C	-6.83195600	5.21325400	-0.43209300	C	1.32674200	-0.02067600	0.93761000
C	-5.73411000	4.61593100	0.19302700	O	8.85599100	-0.46999900	0.25535500
O	-5.18599100	3.48952100	-0.39327200	O	3.83131900	2.09476900	4.35872200
C	-4.21416500	-1.74339900	4.66628500	O	-4.06271500	1.28128300	0.54161700
C	-3.22362500	-0.62044200	4.35390300	H	8.16117200	-3.72646800	-2.39065200
C	-2.61045900	-0.69002100	2.95720800	H	8.33077000	-5.38254000	-1.81291000
O	-2.34361400	-1.85218700	2.49584100	H	8.52212600	-2.88056100	-0.07943700
C	1.39205200	-0.33290000	6.23576000	H	6.24514000	-3.52599000	0.75022100
C	1.15248400	-0.23792100	4.72136000	H	6.24132100	-3.18549900	-0.98264900
C	1.79814200	-1.37882600	3.97054600	H	6.20989700	-4.86604900	-0.40398000
C	3.18029000	-1.38305700	3.74055900	H	8.37792500	-5.83363500	0.74480700
C	1.04025200	-2.46471900	3.51297100	H	9.78780100	-4.77930500	0.92612800
C	3.78703700	-2.43219400	3.04999000	H	8.32315100	-4.45648000	1.85719900
C	1.64538300	-3.52943000	2.84117400	H	10.71850800	-4.89021500	-1.13865000
C	3.02080400	-3.51163800	2.60088200	H	13.14472400	1.21482400	-1.82945100
C	1.45296300	3.40916000	6.97569800	H	13.19912900	-0.05327000	-3.02574300
C	0.19363500	3.43916700	6.09632200	H	11.04556300	2.13021500	-2.93116600
C	0.44198600	3.48799400	4.60021800	H	11.07760800	0.79649800	-4.05832700
C	1.48490700	4.24878200	4.04405600	H	13.39105600	2.96670100	-3.64300900
C	-0.39109100	2.77881300	3.72416500	H	8.48253100	1.08928200	5.33905500
C	1.69919800	4.28105800	2.66104400	H	9.19761600	0.11906200	4.06526200
C	-0.19630800	2.82334000	2.34287400	H	9.51297000	2.17921900	2.66127000
C	0.85797500	3.56551000	1.80562300	H	8.86002700	3.16731400	3.94943200
C	-4.36914300	9.23795900	-0.85986400	H	7.12537600	2.85306600	2.31260100
C	-4.37194600	7.94162500	-1.67722000	H	6.67559800	1.96451400	3.78756000
C	-3.12416600	7.07749700	-1.44838900	H	8.10424800	0.41005900	1.68019300
C	-3.12430700	5.83063700	-2.33979100	H	7.10294600	-0.92875600	0.39520100

H	5.33643200	-0.99445000	0.27929100	H	-9.11887000	0.68500700	-0.16921600
H	4.81608900	1.41901700	2.82756300	H	-6.86219500	-0.02437300	0.52752000
H	4.10424000	0.43968300	1.57748900	H	-6.84584300	1.67326700	-0.01443700
H	10.66091500	2.34416200	5.61850000	H	-6.78765000	1.29984000	1.71218800
H	3.62119900	-6.28281700	-3.96968400	H	-8.90885700	-0.97140700	1.67778300
H	1.91675000	-6.55867400	-3.59681100	H	-8.91288600	0.33698900	2.86889000
H	3.98022200	-3.57605300	-1.63464500	H	-10.36040000	0.03110600	1.90063400
H	4.83553700	-4.75800300	-2.57736100	H	-8.88271300	2.82347900	2.01455400
H	3.29051200	-8.54077100	-2.93168700	H	-6.62235400	8.77870500	2.41187600
H	5.88074300	6.20492400	-0.37935400	H	-7.39806100	9.04860300	0.86511400
H	6.06486500	6.25935200	-2.13270400	H	-5.28976600	6.84036800	2.71639600
H	7.42997300	4.06164000	-3.30057800	H	-8.20564800	6.85933100	-0.43101800
H	8.56198300	1.97077900	-2.14129800	H	-4.33364200	4.73859800	1.82556100
H	8.57768000	2.24024400	0.30191800	H	-7.25484700	4.74275400	-1.31393500
H	8.39560300	7.16994400	-1.86612800	H	-4.86036100	2.81266900	0.25367400
H	3.87074500	5.04521800	-1.54523200	H	-8.71473200	7.66153100	3.27568200
H	2.29533800	4.34346700	-1.24512000	H	1.55429400	0.71346800	4.36165600
H	4.60090900	2.95896800	-2.68709400	H	0.07637100	-0.22348500	4.51423300
H	4.82967600	1.61621800	-0.60701600	H	3.77332700	-0.54159900	4.08222400
H	5.12584900	3.32428600	-0.29039600	H	-0.03630900	-2.45966300	3.65681800
H	3.57704700	2.58555400	0.17261500	H	4.85404600	-2.40156700	2.85130700
H	3.28053100	0.86787300	-2.44078800	H	1.03224100	-4.35328500	2.48867400
H	2.33963700	2.02494500	-3.37071800	H	3.49427300	-4.32566400	2.05982000
H	1.97518300	1.74367800	-1.65784300	H	2.46184500	-0.27981000	6.45887600
H	1.91732100	4.21703400	-3.78701000	H	-0.41904200	4.30486200	6.38309900
H	1.13375700	2.70234500	-5.34978400	H	-0.42025900	2.55774600	6.31127700
H	-0.60320900	2.51753600	-5.18336400	H	-1.20417000	2.17206900	4.11128900
H	2.27885400	0.22990800	-6.07957800	H	2.12613500	4.83897400	4.69313500
H	-1.18944300	0.86209600	-3.62651600	H	-0.85519400	2.25272500	1.69903400
H	2.67954000	-1.93072700	-4.96459000	H	2.51657000	4.86993800	2.25484600
H	-0.78977300	-1.31335200	-2.51271100	H	1.01882100	3.58919400	0.73213000
H	1.13694700	-2.72776000	-3.18025400	H	2.01141400	4.34853900	6.91620200
H	-0.68192500	1.39410400	-7.45672500	H	-4.44431100	8.18559200	-2.74585500
H	-6.92138300	0.36570500	-5.07317200	H	-5.26301900	7.35590900	-1.42806200
H	-7.62430800	-0.71308500	-3.85432300	H	-2.22386900	7.66970200	-1.66436400
H	-5.15244400	1.36965300	-1.82813200	H	-3.06192200	6.77234700	-0.39753700
H	-3.06245900	-0.17298900	-1.47680200	H	-3.33512100	6.11309500	-3.37776800
H	-2.95637200	-1.96542300	-3.20874700	H	-3.91526800	5.14195500	-2.03907100
H	-7.14802000	2.28262000	-3.43195000	H	-1.04134100	5.64661900	-2.65881000
H	-10.62816100	-1.51399200	-0.03609300	H	-2.21625700	2.24412300	-0.89018100
H	-10.71778600	-2.99830900	-0.95007800	H	-3.45106600	3.47363400	-1.06544100
H	-13.01930300	-2.02312300	-0.64364800	H	0.40025300	3.88896200	-2.43000900

H	-0.14076800	2.48637300	-1.54737800
H	-4.34784500	9.02099200	0.21327100
H	-6.88681100	-5.93589200	2.30035700
H	-8.54006700	-3.34503800	2.48145900
H	-8.62115900	-4.48223300	1.15299200
H	-8.24391800	-2.04718400	-0.94625200
H	-4.94371400	-3.86395900	2.35104800
H	-5.55035600	-1.57276900	-1.10383700
H	-1.83612200	-8.77677100	4.42033900
H	-0.84656100	-5.97416800	5.27113700
H	0.02971100	-7.07295300	4.23041600
H	-2.03643200	-7.81603600	2.10112400
H	-1.73239100	-3.91035700	3.61991300
H	-2.92600400	-6.20700000	0.32753800
H	-3.35421900	-4.22901800	-5.89751900
H	-3.98565400	-6.74430900	-4.22958200
H	-5.02970600	-5.34419100	-4.32207100
H	-1.24157300	-5.52660600	-3.67030300
H	-4.71857200	-4.02585100	-1.83464100
H	-0.53433300	-4.35411600	-1.55515200
H	-5.04785200	-1.74198300	3.95764700
H	-2.38732900	-0.64925500	5.06751500
H	-3.68662100	0.36447900	4.46245900
H	1.22828300	0.93072200	1.45506600
H	9.76470900	-0.82570400	0.19200700
H	8.83031500	-0.08056100	-0.68775700
H	4.24011700	2.51969800	5.12245800
H	3.12367700	2.70106700	4.08314000
H	-3.39726900	1.04470500	1.23953200
H	-4.72270000	0.57612000	0.60196300
O	-2.34848200	0.39113600	2.36716600
H	-0.43406600	-0.90592700	1.73601500
H	-5.12445100	-1.83078000	-4.56360400
H	14.74253500	1.82525500	-3.67308100
H	13.44200600	1.69103900	-4.86463100
H	8.23088900	7.07593100	-0.10887500
H	7.22526300	8.21298500	-1.03209200
H	3.51987600	4.88645200	-4.02661600
H	2.27563800	5.87298100	-3.25625800
H	10.88776100	0.60355700	5.84852800
H	11.38282100	1.36857400	4.33259700
H	10.51076200	-3.20511700	-1.65132500
H	10.46021200	-4.51367900	-2.84200100
H	2.57214000	-7.79109200	-1.49332000
H	4.28285200	-7.53039300	-1.86923300
H	-1.18236800	-8.33371800	5.99631100
H	-2.73371600	-7.66150600	5.46126100
H	-6.82989200	-4.78706900	3.64424700
H	-8.31428300	-5.68973900	3.32411900
H	-12.50301500	-0.52000300	-1.43872300
H	-12.59435500	-2.01327300	-2.37010500
H	-8.87332700	3.13013700	0.27555100
H	-10.33327900	2.49608400	1.05462700
H	-9.49548100	7.91414500	1.71030700
H	-9.03994200	9.30871900	2.70289600
H	-8.70494200	1.44806800	-3.66913800
H	-7.76550600	1.13532500	-2.21883100
H	1.06682100	1.59578700	-7.60933700
H	0.02217100	3.01922900	-7.54697800
H	-2.34177800	-5.67383300	-5.81934000
H	-3.94914300	-5.77338300	-6.53220400
H	-3.72919800	-2.71990000	4.59424700
H	-4.62117900	-1.63240100	5.67557400
H	0.89323400	0.48345400	6.76631400
H	1.01453300	-1.28005000	6.63390300
H	-3.49883700	9.85914600	-1.09732800
H	-5.26771700	9.82788600	-1.06197100
H	1.18533000	3.25647400	8.02459800
H	2.12087700	2.59678800	6.67184300
H	1.73419300	-3.33695700	-0.75654200

TS2-Zn (21.4 kcal/mol)

C	-7.25879300	-5.35917600	2.70683200
C	-7.66978900	-4.73305100	1.36119400
C	-6.61338700	-3.77919700	0.89225800
N	-6.82930200	-2.42284300	0.73600700
C	-5.26584500	-4.02926700	0.72850000
C	-5.60667300	-1.90313300	0.52511800
N	-4.61648700	-2.83017600	0.49285000
C	-1.48164700	-8.16326200	4.83151200
C	-1.05590700	-6.69605700	4.67000400
C	-1.42434300	-6.11207100	3.34541500
N	-1.03924100	-6.68437900	2.13865300
C	-2.12112300	-4.98602600	2.98903100

C	-1.49512000	-5.90715400	1.12421000
N	-2.15563900	-4.87113600	1.61047800
C	10.32978800	-4.08777100	-2.08198300
C	8.83002800	-4.28212100	-1.84650500
C	8.35275400	-3.88088500	-0.44006700
C	6.82324200	-3.93445800	-0.36261400
C	8.97545100	-4.74681800	0.66102000
C	13.75281000	2.16308500	-3.86568000
C	13.00530800	1.20814200	-2.92566500
C	11.48571600	1.32505100	-3.04353200
C	10.68668100	0.44154700	-2.07741100
O	11.27080400	-0.38844100	-1.35336000
O	9.40822400	0.64056600	-2.08074800
C	10.79691900	1.24927900	5.06474300
C	9.40493800	0.92549800	4.49411800
C	8.88414000	1.99507000	3.52183300
C	7.44913600	1.78331900	3.02301600
N	7.35558100	0.58977800	2.17790400
C	6.21897500	0.18269700	1.58910000
N	5.02285100	0.67330900	1.93657500
N	6.29073300	-0.76169700	0.64233200
C	3.46582500	-7.53126200	-2.63590700
C	2.94948000	-6.25836400	-3.33495500
C	2.84921600	-5.04338100	-2.41929500
O	1.74213500	-4.63092700	-2.01835800
N	4.00010300	-4.45388300	-2.06418700
C	-3.24084500	-5.12095900	-5.90494400
C	-3.67412000	-5.74728400	-4.56833300
C	-3.03445000	-5.10401000	-3.37993200
N	-1.65786900	-4.98195500	-3.24506900
C	-3.54268600	-4.51987700	-2.24481800
C	-1.38389700	-4.34950400	-2.07670300
N	-2.51029900	-4.04664900	-1.44672700
C	7.75526500	7.30233200	-0.83796900
C	6.66246100	6.23012800	-1.00565600
C	7.21899900	4.83721600	-1.03383100
N	7.39993700	4.09502200	0.12178200
C	7.71144300	4.13432600	-2.11782300
C	7.99581300	2.97901700	-0.27715500
N	8.20386700	2.95272200	-1.61611600
C	2.75576000	4.99423300	-3.16486900
C	2.97712200	4.85667100	-1.65281500
C	3.81864500	3.64087700	-1.22520000
C	3.97224100	3.61063900	0.29864000
C	3.24065400	2.31260000	-1.72904600
C	0.22865900	2.27810700	-7.07206300
C	-0.64500900	2.18940600	-5.80359400
C	-0.37676800	0.95038600	-4.97542700
C	0.59782200	0.96338200	-3.96895300
C	-1.05389000	-0.25311600	-5.21928200
C	0.89243900	-0.17990900	-3.22550400
C	-0.76082400	-1.40637500	-4.48166300
C	0.21412500	-1.37328600	-3.48016000
C	-7.58613500	1.39774500	-3.17263700
C	-7.09367700	0.38305500	-4.21529100
C	-5.83417100	-0.26288900	-3.75002500
N	-5.82466800	-1.09353400	-2.63579900
C	-4.52279000	-0.12281100	-4.12107400
C	-4.56959900	-1.44808300	-2.33589300
N	-3.76686400	-0.86549900	-3.22777400
C	-12.20032100	-1.65019800	-1.34806900
C	-10.77512200	-1.78850100	-0.75984200
C	-9.64921700	-1.44595000	-1.72924100
O	-9.82312200	-1.09067600	-2.87447500
O	-8.36811900	-1.53521700	-1.26895300
C	-9.10480700	2.32315200	1.21748800
C	-8.35202500	1.00386900	1.42484400
C	-6.93038400	1.26135600	1.93545200
C	-9.10382200	0.06554300	2.37621200
C	-8.63100900	8.13107800	2.78078600
C	-7.16616600	8.16808000	2.30444300
C	-6.73173300	6.96491500	1.48884900
C	-5.88633600	5.98383700	2.02207800
C	-7.15085900	6.80302800	0.15959300
C	-5.45127400	4.89369300	1.26314600
C	-6.73453100	5.71900500	-0.61120700
C	-5.87263200	4.76460300	-0.06254800
O	-5.44005800	3.74370200	-0.88131700
C	-4.01525700	-1.96246500	4.62656800
C	-3.74865900	-0.66869700	3.85400500
C	-3.00915300	-0.85859300	2.53344000
O	-2.40616300	-1.96742500	2.35557800
C	1.59436600	-0.56914600	6.19921700
C	1.10844600	-0.58023500	4.73945400

C	1.70363700	-1.71033200	3.93001000
C	3.05149900	-1.66744800	3.54737700
C	0.93381500	-2.81612200	3.54848700
C	3.61775800	-2.70037100	2.80063600
C	1.49813100	-3.85668700	2.80762700
C	2.84116800	-3.80157900	2.43080600
C	1.63386200	3.14207300	7.08212200
C	0.22913700	3.20188100	6.46648100
C	0.18961900	3.18447900	4.95139200
C	1.04434200	3.98922200	4.18063100
C	-0.73844700	2.38151400	4.27688300
C	0.97468900	3.98012300	2.78408900
C	-0.82372300	2.37110500	2.88297300
C	0.04125600	3.16936000	2.13130300
C	-4.30387000	9.22161200	-0.47104800
C	-4.18137300	8.22752800	-1.62844400
C	-3.09072300	7.17492800	-1.39618200
C	-2.94238500	6.24359600	-2.60466700
N	-1.73258700	5.41318100	-2.57645000
C	-1.61730500	4.19243400	-2.02609600
N	-2.67365500	3.53900100	-1.55912700
N	-0.40952400	3.60638900	-1.99109900
Zn	-2.50432000	-3.01332900	0.58156500
O	-0.95550800	1.22550800	-0.57541500
C	-1.18455100	0.04527800	-0.54690100
O	-1.86371700	-0.86490200	-0.93658700
C	0.21739100	-0.79736900	0.82216900
C	0.41099700	-2.10012800	0.25683600
O	-0.44647300	-3.00616300	0.18655200
N	1.63390500	-2.32855700	-0.32758200
C	2.74299900	-1.51535200	-0.16789100
O	3.84174200	-1.88841600	-0.60543900
N	2.58156700	-0.29944100	0.46985100
C	1.38874300	-0.00038200	0.93750600
O	8.99268400	-0.47516500	0.19389200
O	3.59599600	1.92661200	4.12364800
O	-4.11489500	1.52397200	-0.14999800
H	8.27695600	-3.68123400	-2.58179900
H	8.55221000	-5.33153600	-2.03253100
H	8.66300900	-2.84178500	-0.27427600
H	6.45195800	-3.61745400	0.61731900
H	6.37329100	-3.27449700	-1.11306500
H	6.45903000	-4.95634200	-0.53723100
H	8.71816300	-5.80477000	0.52046500
H	10.06563600	-4.66705000	0.67191700
H	8.61255800	-4.44409900	1.64937800
H	10.92901100	-4.73573700	-1.43469700
H	13.29498200	1.40170400	-1.88682500
H	13.29919500	0.17167500	-3.12336100
H	11.16557600	2.36235600	-2.87312400
H	11.15511600	1.08398200	-4.06327800
H	13.49562500	3.20801900	-3.65587000
H	8.68827400	0.81882000	5.32015400
H	9.43207000	-0.04771600	3.98916000
H	9.55402300	2.07381100	2.65563200
H	8.91119200	2.97628300	4.01086100
H	7.12353400	2.66876000	2.46008100
H	6.77900200	1.66303700	3.88511900
H	8.20033600	0.26864700	1.68250800
H	7.22334700	-1.00068700	0.29676800
H	5.45263100	-1.08066900	0.16218600
H	4.87490700	1.27951400	2.73566900
H	4.19088300	0.35658900	1.43311700
H	10.78788000	2.19965500	5.60964500
H	3.60238900	-6.01247800	-4.18060800
H	1.94617300	-6.43088500	-3.73241700
H	3.99786800	-3.60210600	-1.49516600
H	4.88890700	-4.78920200	-2.40143500
H	3.48540000	-8.36664600	-3.34002500
H	5.94887800	6.30551200	-0.17673000
H	6.10234200	6.42489500	-1.92968400
H	7.76690000	4.38021800	-3.16761500
H	8.67696300	2.14814900	-2.08115000
H	8.30442000	2.15662200	0.34845800
H	8.46408300	7.26307800	-1.67078900
H	3.47341400	5.76324500	-1.28797500
H	2.00959200	4.82046600	-1.12389200
H	4.81986700	3.76468300	-1.65747000
H	4.69417500	2.85177700	0.60283800
H	4.33232700	4.56964500	0.68275000
H	3.01138600	3.38070400	0.77844900
H	3.82351400	1.46723000	-1.35426600
H	3.23335800	2.25233600	-2.82213600
H	2.21319200	2.16809300	-1.36526500

H	2.16014400	4.16966000	-3.57959700
H	-0.47063200	3.07427900	-5.17890800
H	-1.70267100	2.21580600	-6.09371200
H	1.14384100	1.88018600	-3.77229200
H	-1.79678400	-0.29537400	-6.01325100
H	1.65837500	-0.13849400	-2.45805500
H	-1.27878700	-2.33631600	-4.70271800
H	0.45921300	-2.26868500	-2.92088200
H	0.05428300	1.41787000	-7.72509000
H	-6.90521100	0.86685700	-5.17783900
H	-7.87125500	-0.37404600	-4.36050000
H	-4.06897600	0.44622500	-4.91455900
H	-2.75139300	-0.93741800	-3.20941300
H	-4.27065900	-2.06983300	-1.50692800
H	-6.82710900	2.15381100	-2.94725400
H	-10.65270200	-1.14492200	0.12023200
H	-10.59849600	-2.80980000	-0.39671500
H	-12.93879300	-1.91195600	-0.58685500
H	-8.27264400	0.49789700	0.45208300
H	-6.41412600	0.32140500	2.14977500
H	-6.33827100	1.81310100	1.20006600
H	-6.95060200	1.85155200	2.85956900
H	-8.60754700	-0.90864000	2.42919000
H	-9.14340600	0.48789100	3.38756600
H	-10.13902300	-0.09171900	2.05135500
H	-9.22520100	2.85321900	2.16966200
H	-6.50744200	8.26243900	3.17499000
H	-7.01247800	9.07468400	1.70578800
H	-5.54601100	6.07694900	3.04989900
H	-7.81078400	7.54373000	-0.28460300
H	-4.78535100	4.15222800	1.69588600
H	-7.06735200	5.59835300	-1.63723000
H	-5.08041900	2.98519500	-0.36213000
H	-8.81162200	7.26247000	3.42171800
H	1.37270600	0.37715200	4.27920400
H	0.01520600	-0.65311600	4.71320400
H	3.64881200	-0.80103300	3.81282600
H	-0.12277500	-2.84597400	3.79810200
H	4.65543000	-2.63404300	2.48755700
H	0.87758900	-4.69357500	2.50486500
H	3.27867500	-4.59924100	1.83721100
H	2.67781100	-0.42192000	6.24330100

H	-0.27670700	4.10805700	6.82628200
H	-0.36471500	2.35971700	6.83810300
H	-1.41009100	1.75373300	4.85638100
H	1.75367600	4.64963500	4.67219800
H	-1.56556900	1.74227700	2.39895700
H	1.64259400	4.61397900	2.20882100
H	-0.01737900	3.16276500	1.04783900
H	2.22144000	4.03333500	6.83971800
H	-3.96135000	8.77050100	-2.55766200
H	-5.14191200	7.72188700	-1.77694000
H	-2.12979700	7.67448400	-1.21562200
H	-3.31864900	6.58610000	-0.49954800
H	-2.88788800	6.83171000	-3.52606100
H	-3.81573100	5.59383500	-2.71123900
H	-0.86977900	5.86828900	-2.83688100
H	-2.57656600	2.63206300	-1.10751200
H	-3.60785900	3.94025300	-1.51279100
H	0.42457000	4.09374500	-2.27740800
H	-0.31974300	2.68112400	-1.58463600
H	-4.56183500	8.70387700	0.45814800
H	-6.32912700	-5.92667600	2.60328500
H	-8.61401600	-4.18901100	1.47257200
H	-7.84630900	-5.52873700	0.62565900
H	-8.14694500	-1.85827000	-0.33757500
H	-4.71551600	-4.95543000	0.80614800
H	-5.42365900	-0.84453800	0.41145400
H	-0.99129100	-8.81170800	4.09678300
H	-1.52536800	-6.08215800	5.44420000
H	0.02769500	-6.60335300	4.82211600
H	-0.50923700	-7.53664800	2.03622400
H	-2.57533500	-4.23622800	3.61487800
H	-1.31556700	-6.10643000	0.07988700
H	-3.47786500	-4.05262500	-5.92926300
H	-3.44744300	-6.82168600	-4.57900600
H	-4.75889900	-5.66647000	-4.45140800
H	-0.95654800	-5.32238400	-3.88612600
H	-4.57326800	-4.43186500	-1.93496600
H	-0.38434100	-4.16780800	-1.72339300
H	-4.57512400	-2.67081800	4.00838800
H	-3.15145200	0.02773900	4.45827900
H	-4.67961800	-0.13818600	3.63025400
H	1.30752600	0.95377900	1.45447800

H	9.91240400	-0.79587600	0.10958800
H	8.96017100	-0.01111400	-0.71178800
H	3.84020600	2.25790200	4.99614600
H	2.86409700	2.49812500	3.84301900
H	-3.76972700	1.05572600	0.66079400
H	-4.45246300	0.81630200	-0.71107600
O	-2.99350600	0.08642900	1.70019100
H	-0.56570900	-0.69095000	1.56348900
H	-6.67599700	-1.36468600	-2.11655700
H	14.83766800	2.05957700	-3.76008800
H	13.50033900	1.96782700	-4.91469000
H	8.31638300	7.12861300	0.08490600
H	7.32662800	8.30989600	-0.79774200
H	3.71155000	4.98685300	-3.69780800
H	2.24971800	5.93425500	-3.41579000
H	11.13068000	0.46817900	5.75399200
H	11.54046700	1.33132600	4.26489900
H	10.62540200	-3.05004100	-1.88847700
H	10.59762500	-4.32435500	-3.11691700
H	2.81964700	-7.80468500	-1.79652500
H	4.47929800	-7.39262800	-2.24818300
H	-1.21074800	-8.53545100	5.82297300
H	-2.56248400	-8.27331800	4.70622500
H	-7.09017100	-4.57851500	3.45461600
H	-8.02943600	-6.03897200	3.08545200
H	-12.38238700	-0.62562500	-1.67985100
H	-12.33032700	-2.30939000	-2.20915800
H	-8.56341400	2.98968700	0.53618000
H	-10.10372000	2.15268000	0.80208700
H	-9.31952600	8.06611400	1.93218200
H	-8.87574200	9.03369000	3.34822600
H	-8.48455100	1.90211700	-3.53613900
H	-7.85267300	0.89181500	-2.24194400
H	1.29115500	2.28770500	-6.81115100
H	0.00438700	3.18819900	-7.63586800
H	-2.16391800	-5.22929800	-6.07325200
H	-3.75187500	-5.60402800	-6.74172600
H	-3.07610200	-2.43707600	4.92240200
H	-4.59701100	-1.76195700	5.53111700
H	1.11624500	0.23517700	6.76628000
H	1.36708000	-1.51737800	6.69634400
H	-3.36512100	9.76256400	-0.31044500
H	-5.08590500	9.96017700	-0.66983800
H	1.57545300	3.07826800	8.17194900
H	2.17787200	2.26401400	6.72079500
H	1.75371800	-3.22805200	-0.81935300

E:P-Zn (2.5 kcal/mol)

N	-6.58105000	-2.37745700	0.53655600
N	-4.36255900	-2.75901000	0.70665000
N	-0.86508300	-6.39465500	2.77828700
N	-1.69000700	-4.49078900	2.06233000
N	7.60833800	0.76132900	1.93268000
N	5.26575700	0.69035000	2.04454100
N	6.42307700	-0.81958500	0.73421900
N	3.76505800	-4.87610700	-1.03167600
N	-1.42454600	-5.28735400	-2.76184500
N	-2.31115300	-4.33652300	-0.98902400
N	6.49266700	3.91327600	-0.60229700
N	8.19186100	2.93278500	-1.65645700
N	-6.02158000	-1.70325400	-3.16049000
N	-4.03180700	-1.68255700	-3.99078200
N	-2.51790300	4.33582800	-0.91032300
N	-3.57813500	2.49058200	-1.85914000
N	-1.84948400	2.16077600	-0.36494200
N	1.58489300	-1.97727100	-0.28853900
N	2.81813800	-0.28096400	0.79582800
C	-6.94915700	-5.00801100	3.29694500
C	-7.48962000	-4.05411300	2.22280900
C	-6.38421000	-3.38510600	1.46410200
C	-5.02655800	-3.61760200	1.56218900
C	-5.33958600	-2.03943600	0.12346800
C	-1.12665800	-7.55641600	5.61158200
C	-0.72395000	-6.10343100	5.29230900
C	-1.08982700	-5.63353700	3.91702000
C	-1.60591800	-4.45058100	3.44545800
C	-1.23918500	-5.67913900	1.68940800
C	10.55758500	-4.19376300	-1.87896500
C	9.08595100	-4.57842700	-1.71232900
C	8.33407400	-3.75103200	-0.65692000
C	6.85413000	-4.14705700	-0.62469300
C	8.94888500	-3.86805800	0.74199100
C	13.92271200	1.84539700	-4.36215400
C	13.16196500	0.93570900	-3.38518100

C	11.68037000	1.29333800	-3.25649000
C	10.88038800	0.43487000	-2.26492100
C	11.11014700	1.86455200	4.66006300
C	9.73011900	1.42538700	4.14078400
C	9.17871600	2.33239500	3.02963500
C	7.72855200	2.04518300	2.62186200
C	6.42860300	0.22991700	1.57087800
C	3.70016600	-7.69627500	-1.95786000
C	2.63231300	-6.65990400	-2.33414100
C	2.68460200	-5.25874100	-1.72053800
C	-3.06681800	-5.66290500	-5.35531400
C	-3.39197000	-6.29617900	-3.98894500
C	-2.78412800	-5.55600400	-2.83971700
C	-3.31374500	-4.96536100	-1.71314900
C	-1.18472700	-4.55643500	-1.64517800
C	7.94924700	7.25700300	-1.79439900
C	6.99820100	6.35130200	-1.00495100
C	7.30810200	4.89013100	-1.14886800
C	8.36503600	4.28988800	-1.80117400
C	7.05876200	2.75768000	-0.93477400
C	2.92452900	4.70173800	-3.78704500
C	3.14050500	4.61749900	-2.27519600
C	2.87462800	3.23298600	-1.66304400
C	3.25086500	3.23273500	-0.17716100
C	1.42637400	2.76232200	-1.86182700
C	0.34996600	1.58337600	-7.34616000
C	-1.06252900	1.23306800	-6.83115300
C	-1.04471300	0.31909700	-5.62332800
C	-1.06525700	0.83828500	-4.31851100
C	-0.95182900	-1.07180800	-5.77793700
C	-0.96740400	0.00184500	-3.20551700
C	-0.84842600	-1.91583900	-4.66578900
C	-0.84924700	-1.38212900	-3.37185400
C	-7.39856800	1.09353400	-3.25133600
C	-7.23727500	0.09218900	-4.40423000
C	-6.03389200	-0.75532000	-4.17454500
C	-4.76734500	-0.74463800	-4.69678300
C	-4.80632300	-2.24809000	-3.05978600
C	-11.97040200	-1.75875400	-1.04457100
C	-10.50509200	-2.03705800	-0.63986200
C	-9.48033800	-1.71191700	-1.71944500
C	-8.85288100	2.47029400	1.04079100
C	-8.80820700	1.02399200	1.55291700
C	-7.77753200	0.85937300	2.67948700
C	-10.19228100	0.56471600	2.02697400
C	-8.38297700	8.41142000	1.97843200
C	-6.91321500	8.30055500	1.53678400
C	-6.61764200	6.98236100	0.85831300
C	-6.05719900	5.90899200	1.56134500
C	-6.96352000	6.77511500	-0.48451700
C	-5.85319800	4.66696200	0.95796700
C	-6.77615300	5.54097100	-1.10161000
C	-6.22772400	4.48153900	-0.37516300
C	-3.69224200	-1.41855100	4.79779700
C	-3.77500000	-0.31766000	3.72825800
C	-2.95710900	-0.55976500	2.45594500
C	1.93519600	0.14918600	6.12709100
C	1.55316000	-0.00502600	4.64718000
C	2.08719900	-1.26597900	4.00154800
C	3.46792500	-1.46961100	3.87406500
C	1.22013300	-2.22497200	3.46495000
C	3.97176900	-2.58880900	3.21382100
C	1.72056000	-3.35177400	2.80782600
C	3.09712900	-3.53616600	2.67740400
C	1.97166300	3.93181500	6.61486200
C	0.50241400	3.95762300	6.17643300
C	0.32879700	3.97006000	4.67481300
C	0.97712600	4.93296000	3.88325400
C	-0.48274200	3.02490200	4.03708300
C	0.83040500	4.93575600	2.49688900
C	-0.65262700	3.03101400	2.65205800
C	0.01674100	3.98271800	1.87980000
C	-4.11099200	9.16754700	-1.43866900
C	-3.86042300	7.77603000	-2.05708100
C	-3.28307000	6.72009200	-1.09024800
C	-3.11312000	5.36387000	-1.79375200
C	-2.65685200	3.00835800	-1.02713300
C	0.39599600	-0.28670800	0.84431800
C	0.36632200	-1.47916600	0.10074700
C	2.81974600	-1.44504400	0.09069000
C	1.64172700	0.24231400	1.15131500
O	11.42998800	-0.48526000	-1.63438800
O	9.62975800	0.75288300	-2.15452400
O	1.70918900	-4.49996100	-1.89260300

O	-9.77666600	-1.38306600	-2.85001400
O	-8.16832500	-1.81706200	-1.39162700
O	-6.07485800	3.27641800	-1.01588100
O	-2.22431600	-1.58891100	2.41377400
O	-0.68038200	-2.12690800	-0.26511800
O	3.86260800	-2.05249100	-0.23214700
O	9.08583200	-0.52027700	-0.01001700
O	3.37173500	2.63217000	3.04447700
O	-5.27505200	1.14419700	0.34926100
O	-3.06065900	0.28707400	1.50818200
Zn	-2.26770400	-2.87534100	0.75878100
H	8.56949800	-4.45470800	-2.67408200
H	9.00524300	-5.64644000	-1.45802000
H	8.40452000	-2.69825800	-0.95683600
H	6.29077400	-3.55880000	0.10696800
H	6.38690600	-4.00339900	-1.60527900
H	6.75559700	-5.20781800	-0.34740600
H	8.93240100	-4.90797300	1.09343400
H	9.98745900	-3.52760200	0.75569800
H	8.39254200	-3.25826800	1.46170200
H	11.14877400	-4.47715300	-1.00248700
H	13.62363500	0.98072700	-2.39264900
H	13.24800200	-0.11025500	-3.69978000
H	11.56447100	2.34135500	-2.94496900
H	11.17633500	1.22570900	-4.22993700
H	13.87777800	2.89413800	-4.04532500
H	9.01602500	1.41744700	4.97593600
H	9.78231700	0.39246300	3.77637800
H	9.81849100	2.27425800	2.13935800
H	9.21670100	3.37687700	3.36280600
H	7.35608300	2.85129700	1.97436400
H	7.10593100	2.02383300	3.52658200
H	8.40338000	0.42448200	1.36461300
H	7.31542500	-1.05005400	0.28945100
H	5.54154300	-1.20388400	0.39080900
H	5.20787300	1.49656200	2.64659800
H	4.38382000	0.32049000	1.65988800
H	11.07066600	2.87902800	5.07202800
H	2.63412400	-6.50013700	-3.41986900
H	1.63259600	-7.04019500	-2.09407200
H	3.84258600	-3.89553000	-0.72575600
H	4.58512900	-5.45870000	-0.97021000
H	3.49643000	-8.64424400	-2.46169600
H	7.02341800	6.62708200	0.05847000
H	5.96375800	6.52988600	-1.32906100
H	9.20355800	4.69932500	-2.34209700
H	8.82621800	2.16789900	-1.98510800
H	6.67654100	1.77678800	-0.69026700
H	7.91772400	7.02098600	-2.86303400
H	4.17630200	4.88797800	-2.04205200
H	2.50320700	5.35798900	-1.76701400
H	3.53534600	2.51460300	-2.16813200
H	3.18543800	2.21771300	0.22355000
H	4.27655700	3.59737500	-0.04851600
H	2.56194000	3.88063200	0.38369700
H	1.24764100	1.81033200	-1.35016500
H	1.17326400	2.62189100	-2.91722100
H	0.72542600	3.50769600	-1.45464600
H	1.88124300	4.51548700	-4.06743700
H	-1.59317800	2.15837300	-6.57853400
H	-1.62902200	0.75668100	-7.64024100
H	-1.14566600	1.91292800	-4.17663000
H	-0.93518500	-1.49460400	-6.77923600
H	-0.95687800	0.42477100	-2.20607200
H	-0.74589600	-2.98816300	-4.80928500
H	-0.75096400	-2.01589300	-2.49759600
H	0.89466400	0.67963900	-7.63507800
H	-7.13400900	0.60859100	-5.36250400
H	-8.12821900	-0.54182300	-4.44789100
H	-4.32499300	-0.15789500	-5.48428700
H	-3.03082200	-1.85462400	-4.10694600
H	-4.48768900	-2.99241600	-2.34906700
H	-6.54956100	1.77883900	-3.18763100
H	-10.23444400	-1.48007600	0.26349000
H	-10.36859900	-3.09579700	-0.38002400
H	-12.64030800	-2.04332600	-0.22972400
H	-8.50387800	0.37578000	0.71541300
H	-7.65293800	-0.19454900	2.95204600
H	-6.79923300	1.25596100	2.39049400
H	-8.09675000	1.40400200	3.57546700
H	-10.17024700	-0.46750700	2.39452600
H	-10.54452300	1.19793900	2.84956300
H	-10.93380900	0.62552100	1.22334000
H	-9.08334500	3.16608200	1.85565400

H	-6.25851600	8.42044400	2.40716800
H	-6.68029800	9.12781600	0.85554300
H	-5.78009000	6.04250300	2.60367000
H	-7.39948100	7.59187100	-1.05365200
H	-5.42591900	3.84003900	1.51728100
H	-7.06862500	5.37641400	-2.13369700
H	-5.83505200	2.55522700	-0.37630200
H	-8.63655100	7.61637100	2.68658300
H	1.93380500	0.86201700	4.09500000
H	0.46175400	0.01113300	4.54408500
H	4.15639800	-0.72131700	4.25383900
H	0.14695300	-2.06639200	3.51910000
H	5.04405000	-2.70455300	3.08954900
H	1.02975100	-4.07040700	2.37811800
H	3.48644400	-4.39407100	2.13720600
H	3.02002200	0.22148300	6.25022200
H	0.01358100	4.84259100	6.60442000
H	-0.02245500	3.08891800	6.58768900
H	-0.98896900	2.27181200	4.63403300
H	1.59985300	5.68675600	4.35558400
H	-1.31053900	2.29238700	2.20510800
H	1.35118900	5.67605800	1.89744200
H	-0.04577400	3.99097700	0.79491800
H	2.49870200	4.83983700	6.30754200
H	-3.18298500	7.88083400	-2.91463100
H	-4.80752500	7.39197600	-2.45438600
H	-2.31121000	7.06172500	-0.70768100
H	-3.95104600	6.59526900	-0.23216500
H	-2.47889300	5.46839200	-2.68235000
H	-4.09428700	5.01764900	-2.12028800
H	-1.93020000	4.66021400	-0.15321200
H	-3.63172300	1.48122500	-1.85828300
H	-4.49471200	2.94928600	-1.88262400
H	-0.97966200	2.49121400	0.02251400
H	-2.22154500	1.30062700	0.05422100
H	-4.81533700	9.09996300	-0.60421200
H	-6.35087100	-5.80717900	2.84801000
H	-8.12452000	-3.28983600	2.69020500
H	-8.14474600	-4.60510500	1.53334400
H	-7.77724500	-2.06045500	-0.44363500
H	-4.48612100	-4.31686600	2.18185100
H	-5.14137700	-1.22975200	-0.56292100
H	-0.61380500	-8.27454800	4.96167600
H	-1.19345100	-5.42187600	6.00778700
H	0.35927200	-5.98689600	5.43046200
H	-0.49138300	-7.33174500	2.76569800
H	-1.90042400	-3.56536800	3.98415700
H	-1.18254200	-6.04014600	0.67562800
H	-3.40148200	-4.62122200	-5.39127100
H	-3.05578900	-7.34124300	-3.98366200
H	-4.47532100	-6.32416600	-3.83800500
H	-0.70257700	-5.57164700	-3.40824000
H	-4.33497700	-4.97483400	-1.35910900
H	-0.20044200	-4.22240500	-1.37356800
H	-4.03933600	-2.37202800	4.39043200
H	-3.46281300	0.65226600	4.13653300
H	-4.81450700	-0.17412700	3.40747100
H	1.70809000	1.15146500	1.74536000
H	9.96046500	-0.94424500	-0.04167300
H	9.15975800	0.00701500	-0.88335000
H	2.75284100	3.22013600	3.49825400
H	3.43808000	2.99689600	2.15126600
H	-4.48731600	0.90094800	0.91197900
H	-5.96391000	0.51404400	0.59791100
H	-0.52647600	0.11790900	1.23083500
H	-6.83824400	-1.87539100	-2.53376800
H	14.97831700	1.56266800	-4.42934000
H	13.49730400	1.78951500	-5.37115000
H	8.98300300	7.13126700	-1.45613300
H	7.68322700	8.31103800	-1.67171200
H	3.54458000	3.96494500	-4.30993200
H	3.19502100	5.69079200	-4.16987100
H	11.46564000	1.19414000	5.44810900
H	11.85385600	1.86068000	3.85599400
H	10.67866600	-3.11320500	-2.01785100
H	10.99965400	-4.69818800	-2.74430900
H	3.71603700	-7.88520000	-0.87982800
H	4.70159500	-7.37496800	-2.26292100
H	-0.85428100	-7.80472600	6.64011200
H	-2.20424100	-7.70394200	5.49584100
H	-6.31537700	-4.47181900	4.01002100
H	-7.76649000	-5.47444100	3.85338800
H	-12.11561500	-0.69805200	-1.26379400
H	-12.23881700	-2.32371900	-1.93955000

H	-7.89890800	2.76732000	0.60765800
H	-9.61998600	2.59346700	0.26904000
H	-9.05541600	8.31787500	1.12000300
H	-8.57689400	9.37446900	2.46025700
H	-8.30411800	1.68901000	-3.39005100
H	-7.49176800	0.57308300	-2.29523400
H	0.93243900	2.09012900	-6.57070300
H	0.28953800	2.24168200	-8.21723500
H	-1.99114500	-5.67576700	-5.56042900
H	-3.56096300	-6.21012300	-6.16194800
H	-2.66373000	-1.54894200	5.14557700
H	-4.31680900	-1.16247300	5.65822100
H	1.48840600	1.05076700	6.55636100
H	1.59315800	-0.70975400	6.71354900
H	-3.18291300	9.60983300	-1.06189600
H	-4.52980900	9.84639400	-2.18607900
H	2.05039000	3.85984000	7.70332800
H	2.49835500	3.07715500	6.18073400
H	1.60938200	-2.83793600	-0.84759900

The binding mode Mode-B considered for the natural 5caU substrate in IDCase

Mode-B (+14.2 kcal/mol)

C	-7.61282000	-5.04866700	2.79678800
C	-8.08177800	-4.03203000	1.75282200
C	-6.93740900	-3.35875900	1.05983200
N	-7.10086900	-2.57056900	-0.06013000
C	-5.59154200	-3.39262600	1.37256400
C	-5.86497500	-2.16578900	-0.38495700
N	-4.89851400	-2.62553700	0.44905900
C	-1.98801100	-7.71499700	5.44345800
C	-1.20504500	-6.54872600	4.79734500
C	-1.76414300	-5.96679900	3.52776800
N	-2.16209200	-6.73385200	2.43922700
C	-1.89484600	-4.66731200	3.09366600
C	-2.50581400	-5.90530200	1.41953900
N	-2.35214100	-4.64175300	1.78376200
C	10.16691500	-4.66642900	-1.40313800
C	8.66877200	-4.98560800	-1.33435000
C	7.86755900	-4.07943700	-0.38213700
C	6.37625900	-4.44477000	-0.42091600
C	8.39142900	-4.13022400	1.05802600
C	13.80412100	1.27922600	-3.72603700
C	12.90213700	0.53550800	-2.73429400
C	11.46069400	0.40382900	-3.22825900
C	10.50633900	-0.27109300	-2.23925200
O	10.96350900	-0.96828300	-1.30779600
O	9.24716800	-0.05862200	-2.44220500
C	10.49753400	1.41876200	5.12674200
C	9.15698400	1.17318900	4.41079600
C	8.95131500	2.06874300	3.18010100
C	7.56329000	1.98258000	2.52998700
N	7.34312600	0.69435800	1.86083900
C	6.20939800	0.01215200	1.79936100
N	5.06509300	0.40927600	2.41636000
N	6.16169900	-1.13306700	1.09237900
C	3.24705300	-8.01236200	-1.84680800
C	3.48983400	-6.49624700	-1.82047800
C	2.23407000	-5.65810300	-1.56905500
O	1.12991900	-5.98147000	-2.02163100
N	2.42595600	-4.52511400	-0.84610100
C	-3.27343400	-5.84535600	-5.62339900
C	-3.54244800	-6.39583200	-4.20592200
C	-3.00120400	-5.55608200	-3.08746300
N	-1.65047900	-5.47853900	-2.76793300
C	-3.61878100	-4.71983200	-2.18183600
C	-1.49571100	-4.63052800	-1.72888200
N	-2.66889300	-4.14199900	-1.34636500
C	7.82434100	6.84071900	-1.52072600
C	6.81627200	5.73619700	-1.86103500
C	7.41955900	4.36838600	-1.76843900
N	7.85018800	3.83864900	-0.56287900
C	7.70868000	3.48767400	-2.79165700
C	8.38886200	2.66893200	-0.87533400
N	8.32722400	2.40847700	-2.20448200
C	2.86046400	4.38939800	-3.77602900
C	3.25372000	4.39973000	-2.28961600
C	4.03209200	3.16017700	-1.80531900
C	4.43123000	3.30614500	-0.33202800
C	3.25202000	1.86027000	-2.01960200
C	0.41500100	1.30836100	-7.45805600
C	0.98470700	1.91899600	-6.17038700

C	1.05010500	0.89526100	-5.06398500
C	2.16466500	0.05877500	-4.92675800
C	-0.03425900	0.70514300	-4.19724900
C	2.20705500	-0.92709500	-3.94025400
C	0.00162000	-0.28220900	-3.20987200
C	1.12872400	-1.10002700	-3.07339300
C	-7.55825800	1.02109000	-3.79762000
C	-6.78074800	-0.19923500	-4.29332700
C	-5.47826300	-0.39149600	-3.58578400
N	-4.67890500	-1.51973100	-3.77784100
C	-4.84022300	0.33423100	-2.61395800
C	-3.62124500	-1.49552400	-2.95392500
N	-3.70278100	-0.37221000	-2.24903200
C	-12.30928300	-1.71348800	-1.83330600
C	-10.86141400	-1.95294300	-1.37712100
C	-9.82150500	-1.48289300	-2.38875400
O	-10.11535000	-0.99374400	-3.46349300
O	-8.52925000	-1.62145000	-2.05669100
C	-9.21590000	2.45695600	0.40074800
C	-8.79834900	0.99377100	0.59254200
C	-7.30830900	0.89812500	0.93127900
C	-9.64827600	0.29828200	1.66102700
C	-8.66001200	8.39156200	1.33408900
C	-7.17020900	8.02107600	1.46042900
C	-6.73445000	6.64789400	0.96610600
C	-5.85161500	5.87270700	1.73230400
C	-7.09333800	6.14643700	-0.29447100
C	-5.30409200	4.67854900	1.25484800
C	-6.55838300	4.95390600	-0.78687900
C	-5.64373500	4.22653300	-0.02157900
O	-5.05020100	3.11224400	-0.59349000
C	-4.36164000	-1.52566200	4.45866000
C	-2.94183700	-0.94059900	4.40122100
C	-2.62602100	-0.69349600	2.93961500
O	-2.28748500	-1.70859300	2.25274000
C	1.21812200	-0.07903300	6.08946800
C	1.29036700	0.04707400	4.56297500
C	1.84643300	-1.19705600	3.90549200
C	3.19232600	-1.55234500	4.09767200
C	1.04721100	-2.02884600	3.11437200
C	3.73242500	-2.68495900	3.48270400
C	1.58058700	-3.16812000	2.50466600
C	2.92522800	-3.49248300	2.67575000
C	1.31420400	3.70560500	6.56012200
C	-0.15033600	3.52019400	6.14016800
C	-0.49104600	3.86716000	4.70319800
C	0.16156600	4.89179400	4.00436200
C	-1.52209900	3.17835200	4.04652300
C	-0.22074400	5.23922100	2.70800800
C	-1.91824200	3.52639700	2.75246000
C	-1.27012500	4.56711200	2.07935500
C	-4.19018900	9.02904900	-1.84416200
C	-4.13535200	7.53259400	-1.52525000
C	-2.76426400	6.89088800	-1.78034900
C	-2.81483600	5.38999700	-1.50176000
N	-1.51152200	4.74992400	-1.74778000
C	-1.27601300	3.44172500	-1.58396200
N	-2.26285700	2.62499800	-1.20870100
N	-0.04513000	2.94458700	-1.78036300
O	-1.65205300	-1.15604900	-0.52560800
C	-0.43583200	-1.45330700	-0.18280200
O	-0.00069700	-2.62084100	-0.15426300
C	0.41217900	-0.32184100	0.30336500
C	1.87612900	-0.36504700	0.40137200
O	2.63868800	-1.21840500	-0.03339300
N	2.41969200	0.75779500	1.06814400
C	1.75538800	1.78136900	1.69072400
O	2.30069000	2.69245400	2.31380800
N	0.38682500	1.72105500	1.55276000
C	-0.24486800	0.70578700	0.90862600
O	8.59795400	-0.75129800	-0.07115500
O	4.17432100	2.31127800	4.25292500
O	-4.07251400	0.82095400	0.30933100
H	8.23405000	-4.89457200	-2.33889600
H	8.52584700	-6.03446300	-1.03437900
H	7.98338700	-3.04888800	-0.73957200
H	5.78836000	-3.79963200	0.24513000
H	5.96944000	-4.35749700	-1.43493300
H	6.22687100	-5.47941400	-0.08626600
H	8.35441500	-5.15336900	1.45315600
H	9.42613700	-3.78402400	1.12008200
H	7.78999700	-3.49384900	1.71660300
H	10.67620700	-4.92301100	-0.46896500
H	12.90013500	1.05905000	-1.77078900

H	13.29858200	-0.46342500	-2.52661800
H	11.04529800	1.38907500	-3.47484300
H	11.42821900	-0.17186500	-4.16404000
H	13.43311200	2.29308900	-3.91646500
H	8.33279000	1.34699500	5.11707200
H	9.08413500	0.12045700	4.11055000
H	9.71763400	1.85162700	2.42434000
H	9.10075900	3.11840000	3.46167100
H	7.45194200	2.78142100	1.78563100
H	6.79231500	2.12509100	3.29320200
H	8.05980400	0.34156600	1.19611300
H	6.99470900	-1.33914300	0.52273700
H	5.25192500	-1.45316100	0.78705300
H	5.06978400	1.17644600	3.09438000
H	4.46254400	-0.35692100	2.70701700
H	10.57280500	2.45464800	5.47453000
H	4.25914800	-6.24353200	-1.08531600
H	3.88278100	-6.17312900	-2.79397800
H	1.67636800	-3.86085000	-0.66946800
H	3.34641400	-4.25770800	-0.53354000
H	4.15739200	-8.53747200	-2.14564100
H	5.96390200	5.81594700	-1.17431800
H	6.41916400	5.88578200	-2.87263900
H	7.54974600	3.55067800	-3.85752300
H	8.67654600	1.51272900	-2.60335000
H	8.85027900	1.97173000	-0.19256700
H	8.66189200	6.83156900	-2.22508800
H	3.86036100	5.29001700	-2.09208100
H	2.35910000	4.52619300	-1.65149900
H	4.95564000	3.10776800	-2.39609600
H	4.98310000	2.42265800	0.00526500
H	5.09054800	4.16003300	-0.17093900
H	3.55247000	3.42361500	0.31114400
H	3.83372600	0.99088100	-1.69908300
H	2.97868100	1.69726600	-3.06260900
H	2.33411000	1.85415700	-1.41990900
H	2.16526800	3.57816000	-4.01870200
H	1.98501800	2.32115600	-6.36483900
H	0.36085800	2.76526200	-5.85789000
H	3.01250200	0.19217100	-5.59355800
H	-0.91790200	1.33135700	-4.30749800
H	3.08823600	-1.55245600	-3.83765300
H	-0.84186400	-0.42195300	-2.54403500
H	1.17048100	-1.85269200	-2.29604300
H	-0.59553700	0.92189700	-7.29041300
H	-6.60218500	-0.13282400	-5.37457800
H	-7.40093300	-1.08309700	-4.10991800
H	-5.11296100	1.26318600	-2.14005800
H	-3.10567900	-0.20660200	-1.43128300
H	-2.87254300	-2.25933200	-2.82392600
H	-7.03793200	1.95751200	-4.02626700
H	-10.65871800	-1.44624000	-0.42531800
H	-10.67914000	-3.01860000	-1.18347500
H	-13.01114300	-2.07446300	-1.07669900
H	-8.94871000	0.46303400	-0.35697100
H	-7.00703300	-0.14298800	1.07923000
H	-6.71536400	1.32205000	0.11684000
H	-7.07391100	1.46121300	1.84344200
H	-9.36252000	-0.75453900	1.76344400
H	-9.51530200	0.77697200	2.63882600
H	-10.71493800	0.34048300	1.41506700
H	-9.06699100	3.02759500	1.32533700
H	-6.86900900	8.11578500	2.50956200
H	-6.58125800	8.77417000	0.91731200
H	-5.56178700	6.21933600	2.72050000
H	-7.78764500	6.70284200	-0.91727600
H	-4.60809600	4.11591200	1.86916700
H	-6.83523900	4.58228800	-1.76829900
H	-4.85128000	2.38080800	0.04489700
H	-9.28932400	7.69526100	1.89642200
H	1.92557500	0.90452800	4.31766300
H	0.29389700	0.26077400	4.15978800
H	3.81495500	-0.93597200	4.74257000
H	0.00261200	-1.78373800	2.95155000
H	4.77856300	-2.93634400	3.63343700
H	0.94415800	-3.77590100	1.87308000
H	3.34076400	-4.37061400	2.19060000
H	2.20764100	-0.27543500	6.51233300
H	-0.78538400	4.12993300	6.79783900
H	-0.45643600	2.48438800	6.32020600
H	-2.02702200	2.35526900	4.54266100
H	0.97975100	5.42764400	4.47364600
H	-2.70307700	2.94923900	2.27643700
H	0.30567600	6.03478000	2.18958700

H	-1.56505500	4.83926600	1.07125400
H	1.60812600	4.75963500	6.54230200
H	-4.88875200	7.00543100	-2.12096800
H	-4.41549300	7.36507500	-0.47971600
H	-2.45967400	7.06084400	-2.82204400
H	-2.00480400	7.35838100	-1.14024200
H	-3.57481500	4.91993000	-2.13620300
H	-3.10806700	5.21439900	-0.46212900
H	-0.78947300	5.30373500	-2.18632300
H	-2.04529900	1.69709200	-0.87393500
H	-3.22069700	2.95388200	-1.05947800
H	0.73125500	3.54568000	-2.01739300
H	0.08709900	1.95673700	-1.96879300
H	-3.47407500	9.59398100	-1.23770400
H	-6.99127600	-5.82341100	2.33575200
H	-8.72262000	-3.27977000	2.23386400
H	-8.71478100	-4.52707200	1.00518300
H	-8.26542100	-2.05054100	-1.17366600
H	-5.07545300	-3.91369300	2.16449700
H	-5.67310300	-1.52371300	-1.22805800
H	-2.03947000	-8.58696800	4.78190300
H	-1.12035200	-5.72583800	5.51379000
H	-0.17605200	-6.88190400	4.60397400
H	-2.19585700	-7.74208000	2.41181200
H	-1.67907300	-3.74712700	3.61127600
H	-2.86114100	-6.24410100	0.45862600
H	-3.72296100	-4.85443300	-5.74891900
H	-3.12918400	-7.40936400	-4.13021700
H	-4.62169600	-6.49684000	-4.05299100
H	-0.86544300	-5.99535900	-3.14821500
H	-4.67077000	-4.50343200	-2.05958000
H	-0.54426600	-4.39918000	-1.28884700
H	-5.08109000	-0.83654400	4.00615100
H	-2.22117500	-1.64646000	4.82622900
H	-2.88816500	-0.00148300	4.95753900
H	-1.32391900	0.70492700	0.99279200
H	9.51963300	-1.10005300	-0.09111300
H	8.60130300	-0.44834300	-1.04243900
H	4.49934700	3.11338800	4.67787700
H	3.53473800	2.60412200	3.56934900
H	-3.53500900	0.66911600	1.15336200
H	-4.75501100	0.13198500	0.34937600
O	-2.78826700	0.47752400	2.49114600
H	-0.15563400	2.41735600	2.05853900
H	-4.89471000	-2.28473100	-4.40202900
H	14.82783300	1.36474600	-3.34757600
H	13.84837200	0.75949200	-4.69037600
H	8.23093100	6.67836100	-0.51876500
H	7.35983200	7.83230000	-1.55199500
H	3.74695700	4.24877600	-4.40273000
H	2.39397700	5.33488400	-4.07298200
H	10.60498300	0.76214800	5.99485500
H	11.34258500	1.23021500	4.45624300
H	10.34237800	-3.60090500	-1.58994200
H	10.64919600	-5.23628000	-2.20355200
H	2.45013300	-8.25575100	-2.55343400
H	2.94687800	-8.38047200	-0.86095200
H	-1.48826400	-8.03184200	6.36177400
H	-3.01103800	-7.41758700	5.69096500
H	-7.01883600	-4.56335900	3.57831200
H	-8.46234200	-5.53824600	3.28104300
H	-12.48916300	-0.64865900	-1.99918400
H	-12.50898900	-2.23040500	-2.77457200
H	-8.62219100	2.94157600	-0.38110000
H	-10.27211800	2.53820700	0.12322600
H	-8.99443900	8.37692600	0.29193700
H	-8.82957000	9.39906600	1.72303300
H	-8.54405200	1.03131700	-4.26254500
H	-7.71371700	0.95215500	-2.71796700
H	1.03628900	0.47419900	-7.79777200
H	0.36875400	2.04840500	-8.26292300
H	-2.20070800	-5.75184600	-5.81869000
H	-3.69935100	-6.51044400	-6.37896900
H	-4.40730000	-2.46659400	3.90588200
H	-4.67150300	-1.71224900	5.49093600
H	0.83937800	0.83807700	6.54585100
H	0.56190900	-0.90489700	6.38287400
H	-3.96149300	9.22237600	-2.89765800
H	-5.18760200	9.42881400	-1.64030400
H	1.46209000	3.34519900	7.58181700
H	1.99439000	3.15371100	5.90219000
H	3.43419100	0.76822900	1.18505000
Mn	-2.76771100	-2.81068200	0.50270300

The binding mode Mode-A considered for the non-natural γ-resorcylate substrate in IDCase

Mode-A (0.0 kcal/mol)

C	-7.72598200	-4.48973700	3.35851400
C	-8.12944400	-3.53127700	2.23045600
C	-6.95272500	-2.94875300	1.50358400
N	-7.03851700	-2.49885000	0.20080700
C	-5.65368100	-2.76907100	1.93887400
C	-5.80036000	-2.07775100	-0.10091300
N	-4.90411200	-2.20844300	0.91152600
C	-2.15677200	-7.02795700	6.23853300
C	-1.27621600	-6.25695100	5.23703500
C	-2.01824700	-5.53493800	4.15185500
N	-3.02597300	-6.12346700	3.39775600
C	-1.86993900	-4.28111300	3.60790800
C	-3.43949700	-5.23731400	2.45525600
N	-2.75538400	-4.11052300	2.55711300
C	10.06985200	-4.76118600	-0.78199300
C	8.75165000	-4.85250100	-1.55885800
C	7.64927600	-3.88941800	-1.07983100
C	6.40556700	-4.01748900	-1.97094300
C	7.27951400	-4.12582100	0.38980200
C	13.81802400	0.91049300	-3.56721400
C	12.98113600	0.14747400	-2.52824300
C	11.48018200	0.19631900	-2.81905100
C	10.57927100	-0.47636900	-1.77408700
O	11.07074400	-1.15049000	-0.84868300
O	9.30890100	-0.26979900	-1.93363100
C	10.48897600	1.83103200	5.23023900
C	9.11701800	1.58812000	4.57817800
C	8.90779500	2.38285700	3.28047200
C	7.51191700	2.25600300	2.65749900
N	7.27846900	0.89528000	2.15867200
C	6.08193200	0.36696300	1.89079300
N	4.93605200	0.95533500	2.26680500
N	6.03306300	-0.78654300	1.20260200
C	3.09317600	-8.01143700	-0.98026300
C	2.67275200	-7.49105000	-2.37036600
C	1.99028400	-6.13040500	-2.31204600

O	0.75944200	-6.00663100	-2.29926300
N	2.81167600	-5.05580800	-2.25242100
C	-3.37715100	-6.04776100	-4.94956600
C	-3.95095200	-6.28074000	-3.54053100
C	-3.32939100	-5.42370000	-2.48297600
N	-1.97572200	-5.45543300	-2.18380300
C	-3.85871700	-4.49218500	-1.61605200
C	-1.73357500	-4.57837000	-1.18580700
N	-2.85289600	-3.96560800	-0.81602800
C	7.93107500	6.73672100	-1.84967000
C	6.92388600	5.58053800	-1.94901400
C	7.55064300	4.23841300	-1.72163900
N	7.94942800	3.82252400	-0.46146300
C	7.85559000	3.26264000	-2.64921600
C	8.47887300	2.62152000	-0.64777200
N	8.44486900	2.23647000	-1.94692300
C	2.93102100	4.19581800	-3.91832600
C	3.45431900	3.81458500	-2.52647000
C	4.29290800	2.52502300	-2.47631400
C	4.77811600	2.26875900	-1.04466000
C	3.53286100	1.31568600	-3.03552500
C	0.44230700	0.86677600	-7.34691200
C	0.96266400	0.93836300	-5.90096800
C	0.72786600	-0.31403500	-5.07891900
C	1.23288700	-1.55324200	-5.49962700
C	0.02935300	-0.26568600	-3.86506700
C	1.04019800	-2.70527200	-4.73781000
C	-0.15398500	-1.41481000	-3.09083800
C	0.34505000	-2.64293500	-3.52733600
C	-7.54531500	1.01843800	-3.70961300
C	-6.81082200	-0.25830200	-4.11319500
C	-5.48059900	-0.41770700	-3.45072400
N	-4.67303000	-1.53033700	-3.69002100
C	-4.80265100	0.31615000	-2.51164100
C	-3.56917900	-1.48625000	-2.93180300
N	-3.62993700	-0.36594900	-2.21871900
C	-12.34947800	-1.46341900	-1.54882400
C	-10.92019600	-1.84469700	-1.12001000
C	-9.85233000	-1.41682300	-2.12215900
O	-10.12294900	-0.86298800	-3.17125900
O	-8.56698800	-1.67781700	-1.83328900
C	-9.18918900	2.82138200	0.35016000

C	-8.88393000	1.32413900	0.28561800
C	-7.37397800	1.09286200	0.24820800
C	-9.51066200	0.56594900	1.46130000
C	-8.53146400	8.80196600	0.79780200
C	-8.65261100	7.32386300	1.23324900
C	-7.57505300	6.34621500	0.77691800
C	-6.87532400	5.56586500	1.70873900
C	-7.31895500	6.09357700	-0.58027200
C	-5.98332000	4.56100900	1.32090900
C	-6.43689900	5.08983100	-0.98646200
C	-5.77629900	4.30599700	-0.03523100
O	-4.92934100	3.30631800	-0.48339100
C	-4.41781800	-0.89957600	4.74089200
C	-3.36907100	0.20537300	4.66842700
C	-2.53382100	0.13204600	3.41541900
O	-2.37915700	-0.89234100	2.75589600
C	1.18186600	0.57847700	6.27200700
C	1.38518000	0.66682300	4.74908000
C	1.96629000	-0.61723200	4.20466100
C	3.33539900	-0.88369700	4.34402400
C	1.15072600	-1.59867900	3.62595100
C	3.87748400	-2.09777100	3.91608900
C	1.68351300	-2.82233700	3.21940400
C	3.04909800	-3.07432700	3.36009100
C	1.34335700	4.38658600	6.43220000
C	0.41058000	4.31641500	5.21962700
C	1.08270700	4.47482300	3.86958300
C	2.31209400	5.13294500	3.71211400
C	0.45510400	3.97065400	2.72154800
C	2.91285400	5.25318400	2.45231800
C	1.03926400	4.11307200	1.46420900
C	2.28084500	4.73520000	1.32220100
C	-4.04182800	9.09952100	-2.40260000
C	-4.15167000	7.58198100	-2.26098200
C	-2.82614500	6.86163500	-2.53810300
C	-2.94999100	5.36361200	-2.27432000
N	-1.67437700	4.66940700	-2.48135500
C	-1.29792800	3.56474100	-1.81877000
N	-2.12686300	2.95660600	-0.98158700
N	-0.06227000	3.05937100	-2.03572400
O	-0.86623500	1.22586200	0.66919500
C	-0.90872300	-0.02105500	0.30797400

O	-2.03980700	-0.52856800	-0.03861500
C	0.32955300	-0.77300200	0.25581900
C	0.33859400	-2.20584900	0.37124500
O	-0.68789400	-2.87392300	0.77925600
C	1.55717300	-0.08191600	0.03842100
O	8.65691900	-0.84905700	0.47913900
O	4.24964100	2.74034000	4.37972200
O	-4.26519900	0.98959200	0.78859200
H	8.94840700	-4.64633500	-2.61880900
H	8.36379200	-5.88185800	-1.51424400
H	8.03236000	-2.86562500	-1.17588200
H	5.63112700	-3.29645400	-1.67931000
H	6.64509500	-3.84146000	-3.02438100
H	5.98376900	-5.03123200	-1.88859100
H	7.01424000	-5.17679500	0.56354900
H	8.10428500	-3.86980900	1.05871300
H	6.41537100	-3.51952300	0.68730600
H	9.94744400	-5.06817900	0.26197000
H	13.16092500	0.56019800	-1.52904100
H	13.30147500	-0.89861000	-2.47584600
H	11.14405100	1.23790900	-2.91259100
H	11.25822300	-0.26681900	-3.79045100
H	13.53291000	1.96828300	-3.60564100
H	8.32354400	1.85768900	5.28991900
H	8.98875300	0.51823000	4.37324300
H	9.66178900	2.08987300	2.53776100
H	9.07781700	3.44968700	3.46934300
H	7.40244300	2.97568900	1.83581700
H	6.75584100	2.48889600	3.41502800
H	8.05065500	0.40360000	1.68103600
H	6.92252900	-1.17963700	0.86890200
H	5.17338900	-1.31063200	1.17462400
H	4.90650800	1.67326100	2.98871000
H	4.07625600	0.44299600	2.13077500
H	10.62409600	2.88883600	5.48066700
H	3.55054500	-7.43307800	-3.02413200
H	1.95987400	-8.18022400	-2.82873500
H	2.40947600	-4.13284800	-2.15277400
H	3.81677800	-5.14148300	-2.25620800
H	3.54076200	-9.00425200	-1.06874700
H	6.12690600	5.73322000	-1.20895500
H	6.44458900	5.59120600	-2.93575100

H	7.71699900	3.22207900	-3.71890100
H	8.77289400	1.29370500	-2.24590000
H	8.90493100	1.98269000	0.11109400
H	8.70952100	6.63618400	-2.61227300
H	4.06305100	4.63956700	-2.13810500
H	2.61913400	3.70315000	-1.81681000
H	5.18053900	2.68898600	-3.10093300
H	5.40295700	1.37043500	-1.00096700
H	5.39135900	3.09530800	-0.67789100
H	3.93229000	2.13357900	-0.36388100
H	4.13147400	0.40430100	-2.94249700
H	3.29220800	1.43707700	-4.09530000
H	2.59623800	1.14304800	-2.49419600
H	2.22917200	3.45280300	-4.31467900
H	2.03945400	1.14874600	-5.92599500
H	0.50069700	1.78991600	-5.38704000
H	1.79055500	-1.61491200	-6.43003700
H	-0.35810100	0.68861200	-3.51570200
H	1.44375800	-3.65387500	-5.07986100
H	-0.65906100	-1.34957000	-2.13388600
H	0.20725500	-3.54134000	-2.93497400
H	-0.63804000	0.69099900	-7.36742400
H	-6.67326400	-0.29591300	-5.20197800
H	-7.43919700	-1.10868800	-3.82684300
H	-5.05925600	1.23761100	-2.01476600
H	-2.96759800	-0.17315800	-1.44621300
H	-2.78306300	-2.22038600	-2.89115200
H	-7.00395300	1.91734200	-4.02379800
H	-10.67021800	-1.40167000	-0.14893100
H	-10.82742600	-2.93005400	-0.98032000
H	-13.07190600	-1.80356400	-0.80209500
H	-9.31035900	0.92811700	-0.64629100
H	-7.14414900	0.03613700	0.10355300
H	-6.91193200	1.66518200	-0.56002500
H	-6.91811300	1.42567500	1.18930400
H	-9.24826800	-0.49717600	1.42889000
H	-9.14471700	0.96500500	2.41543700
H	-10.60240000	0.65370700	1.46260100
H	-8.75747600	3.26905400	1.25214200
H	-9.61905900	6.94143500	0.87898900
H	-8.70341700	7.28453100	2.32685200
H	-7.04438900	5.73055700	2.76948500

H	-7.84380300	6.66647600	-1.34005400
H	-5.46648000	3.96473900	2.06756500
H	-6.28466500	4.87989700	-2.04039300
H	-4.83838600	2.55687700	0.15538300
H	-8.52326200	8.90032100	-0.29268700
H	2.05668000	1.50209500	4.52657900
H	0.42900200	0.88135600	4.25943500
H	3.97717800	-0.12894100	4.78902600
H	0.09277300	-1.39951700	3.47539000
H	4.94273700	-2.28174700	4.02390200
H	1.03025800	-3.55982300	2.76624700
H	3.46667100	-4.02106100	3.03037800
H	2.13439500	0.40076500	6.78008800
H	-0.36665300	5.08669000	5.31856200
H	-0.12272500	3.35900400	5.22091400
H	-0.50122300	3.46532600	2.82243100
H	2.80111100	5.57188100	4.57665900
H	0.52372200	3.73390200	0.58910100
H	3.86982400	5.75769100	2.35610400
H	2.74892100	4.81577100	0.34690600
H	1.77302300	5.38497300	6.55924800
H	-4.92057200	7.19907500	-2.94384300
H	-4.49574300	7.32787000	-1.25166800
H	-2.51614200	7.03946100	-3.57719500
H	-2.03748600	7.27068900	-1.89461700
H	-3.72864100	4.91833300	-2.90578700
H	-3.25433300	5.21411200	-1.23656800
H	-1.07018000	5.00802000	-3.21659500
H	-1.75040000	2.26109900	-0.31228300
H	-3.09821500	3.25662400	-0.88460900
H	0.65012300	3.63486600	-2.46096400
H	0.29363200	2.29704800	-1.46624500
H	-3.30750600	9.50949400	-1.70088200
H	-7.11432400	-5.31082700	2.96969700
H	-8.75566200	-2.72836100	2.64483300
H	-8.76086500	-4.05607100	1.50351900
H	-8.28730700	-2.07728300	-0.94841800
H	-5.20283300	-3.00973800	2.88726100
H	-5.54784600	-1.65875900	-1.06133200
H	-2.72610300	-7.82625300	5.74919600
H	-0.67272600	-5.51473100	5.76840200
H	-0.56110800	-6.95467000	4.77978600

H	-3.40316400	-7.04820100	3.54093000
H	-1.17818500	-3.50328000	3.89052800
H	-4.22855700	-5.43017500	1.74458300
H	-3.51628300	-5.00773300	-5.26406300
H	-3.82997700	-7.33893400	-3.27432100
H	-5.02883900	-6.08918200	-3.53810800
H	-1.22863200	-6.01398200	-2.58749700
H	-4.88587000	-4.17716000	-1.49952900
H	-0.76455500	-4.41815100	-0.74865300
H	-5.12939200	-0.82126000	3.91554400
H	-2.66984500	0.14245600	5.51374400
H	-3.81578400	1.20245000	4.71955100
H	9.54635800	-1.25293000	0.52691400
H	8.71572100	-0.57573100	-0.50096500
H	4.77268400	3.17174800	5.06603700
H	3.70315100	3.44328800	3.98811000
H	-3.41193300	0.54472300	0.60750000
H	-4.88240600	0.25271400	0.91965100
O	-1.94974600	1.28707000	3.11698500
H	-1.46963400	1.22271500	2.24783300
H	-4.90432200	-2.28942900	-4.31552200
H	14.88583400	0.86072100	-3.33140600
H	13.67865900	0.49580600	-4.57247400
H	8.42012400	6.72597500	-0.87141500
H	7.44182900	7.70770500	-1.98317400
H	3.75532600	4.26920200	-4.63515200
H	2.42711900	5.17015600	-3.90600200
H	10.59579000	1.24960900	6.15058000
H	11.30166500	1.54322800	4.55498700
H	10.45982000	-3.73685900	-0.79177200
H	10.82678900	-5.41446100	-1.22716900
H	2.22691900	-8.08283100	-0.31610000
H	3.82718000	-7.34803500	-0.51250800
H	-1.53505900	-7.49774200	7.00461700
H	-2.86869200	-6.36132100	6.73373700
H	-7.14097500	-3.97288900	4.12655600
H	-8.60611400	-4.91881600	3.84583400
H	-12.44126900	-0.38031800	-1.65922800
H	-12.59671700	-1.91473900	-2.51204600
H	-8.76684900	3.35154600	-0.50840700
H	-10.26831600	3.00984100	0.36626700
H	-7.61098400	9.25299100	1.18135400
H	-9.38049100	9.37462200	1.17993500
H	-8.53930200	1.01781100	-4.15657400
H	-7.68295100	1.04811200	-2.62678400
H	0.92058800	0.05512400	-7.90268300
H	0.64815100	1.80039300	-7.87875500
H	-2.30402900	-6.26033700	-4.98522100
H	-3.87045600	-6.69396500	-5.68043400
H	-3.94491600	-1.88283800	4.67678400
H	-4.97161700	-0.83888600	5.68129100
H	0.75149800	1.50217200	6.67154200
H	0.51236200	-0.24987800	6.52689300
H	-3.73190300	9.38585100	-3.41356500
H	-5.00177700	9.58246200	-2.19931800
H	0.79811000	4.14982000	7.34980700
H	2.16704100	3.67170900	6.33316500
C	1.55606100	-2.87944300	0.07419400
H	1.56430200	-3.96116000	0.15034700
C	2.73881600	-0.76789000	-0.21142500
H	3.64190300	-0.21300000	-0.42573700
C	2.70906000	-2.17053800	-0.21104300
H	3.63071000	-2.71415700	-0.40970600
O	1.58636100	1.28305900	0.01797500
H	0.76400600	1.56067500	0.48997300
Mn	-2.72571100	-2.51316000	0.96748900

The binding mode Mode-B considered for the non-natural γ-resorcylate substrate in IDCase

Mode-B (-18.5 kcal/mol)

C	-7.47714400	-4.78989700	3.47214400
C	-7.97353000	-3.92736800	2.31132700
C	-6.83712900	-3.28529600	1.58097600
N	-7.00367000	-2.51770600	0.44623700
C	-5.49089300	-3.33281700	1.87986700
C	-5.76870400	-2.13253800	0.09938300
N	-4.79984800	-2.59394100	0.92892100
C	-1.80233700	-7.06099600	6.36901200
C	-1.02831600	-5.91966200	5.68095000
C	-1.55970000	-5.49513800	4.34290200
N	-1.97033700	-6.39776600	3.37013100

C	-1.69192700	-4.26325100	3.74713100
C	-2.32516300	-5.71264500	2.25555000
N	-2.16207600	-4.41232400	2.45065500
C	10.28289900	-4.58109400	-0.82188400
C	8.82492500	-4.86003700	-1.21156000
C	7.78494700	-3.99505400	-0.47485200
C	6.37172100	-4.25956600	-1.01894400
C	7.81692000	-4.21272000	1.04269200
C	13.81563900	1.13372700	-3.79423800
C	12.94713700	0.40823700	-2.75666700
C	11.45190100	0.69769800	-2.90648000
C	10.55289800	0.00518800	-1.87533000
O	11.05957200	-0.72741200	-1.00054000
O	9.28449800	0.23770900	-1.98719900
C	10.52998400	2.18384400	5.00499100
C	9.22152300	2.04272400	4.22121400
C	9.28903600	2.68763700	2.83380100
C	7.97548300	2.61066700	2.04963200
N	7.62113600	1.22806000	1.73777500
C	6.53365400	0.57220000	2.13000300
N	5.50064300	1.20004800	2.73204700
N	6.49575300	-0.76128100	1.93498400
C	3.41820700	-8.06633700	-0.86809000
C	3.33601700	-7.15559900	-2.10732600
C	2.31835700	-6.03963800	-1.91992300
O	1.09996700	-6.25200800	-2.00627600
N	2.81597400	-4.81990200	-1.62235000
C	-3.14692800	-6.42943600	-4.82993900
C	-3.58197800	-6.72420400	-3.38219300
C	-2.98079800	-5.82879500	-2.34127600
N	-1.62667800	-5.79983900	-2.04378500
C	-3.55123600	-4.93436200	-1.45884600
C	-1.42494400	-4.92693400	-1.03184200
N	-2.57132000	-4.38052000	-0.64793200
C	7.75069400	6.80525600	-2.18178000
C	6.74906700	5.93836500	-1.40316700
C	7.02751000	4.46394700	-1.47525900
N	6.20925400	3.52543900	-0.85761000
C	8.04919300	3.80126500	-2.12083500
C	6.73903400	2.33935000	-1.14724000
N	7.84893300	2.45493500	-1.90458500
C	2.82190100	4.04312900	-4.13505900
C	3.64812300	2.91926800	-3.50829500
C	2.94753100	1.55004900	-3.51794000
C	3.92934800	0.44947100	-3.09974900
C	1.69053400	1.52685500	-2.63683000
C	0.41835200	0.54028200	-7.44882800
C	-0.21352800	0.50556600	-6.04333600
C	-0.08254900	-0.82620000	-5.33052300
C	1.17826900	-1.30599600	-4.94332700
C	-1.20356100	-1.60935600	-5.02752300
C	1.31316200	-2.51448300	-4.26357600
C	-1.07101000	-2.83079800	-4.35722300
C	0.18753100	-3.28598000	-3.96477700
C	-7.53963500	0.52514100	-3.74426200
C	-6.65680500	-0.69951400	-3.97022200
C	-5.37749700	-0.66442100	-3.19871500
N	-4.52199200	-1.76482700	-3.16142700
C	-4.79152200	0.26853800	-2.37967600
C	-3.48100300	-1.52557900	-2.35157600
N	-3.62807900	-0.29501700	-1.87281600
C	-12.23992500	-2.05528600	-1.47311900
C	-10.79394900	-2.16903200	-0.96475200
C	-9.75437500	-1.82837100	-2.02670600
O	-10.04945500	-1.51312500	-3.16337000
O	-8.46141000	-1.87929100	-1.66879700
C	-9.20996300	2.38308200	0.27986700
C	-8.45334300	1.07039200	0.51759300
C	-6.96252500	1.34026000	0.75024300
C	-9.04686200	0.27759200	1.68744200
C	-8.74965800	8.39226000	0.55894200
C	-7.30435100	8.39196000	0.02160100
C	-6.67674900	7.02588300	-0.19752300
C	-5.95351800	6.38172000	0.81632400
C	-6.75498800	6.37552700	-1.43763100
C	-5.30117700	5.16599100	0.59925100
C	-6.11038600	5.15990500	-1.67504800
C	-5.35647500	4.56021400	-0.66241000
O	-4.66465100	3.40506800	-0.95002900
C	-4.28005800	-1.05477500	4.72612800
C	-4.10568100	-0.24455400	3.43245200
C	-2.69144300	-0.27743100	2.85662500
O	-2.18086100	-1.40046600	2.54909600
C	1.27912500	0.65083300	6.16546500

C	0.90675500	0.12023500	4.77700800
C	1.84810200	-0.91688200	4.19622000
C	3.20352300	-0.97959200	4.54357100
C	1.36079900	-1.83874800	3.25622900
C	4.04863200	-1.93923200	3.97393000
C	2.20140400	-2.78779700	2.67763500
C	3.55154800	-2.84519200	3.03522700
C	1.31388700	4.46469100	6.22060100
C	1.39407900	4.16481300	4.71213900
C	0.19706800	4.69684100	3.95731700
C	0.19958100	6.00103800	3.43980200
C	-0.95808100	3.91928200	3.79365200
C	-0.92678500	6.52095000	2.79309600
C	-2.08226600	4.43113800	3.14399900
C	-2.07391200	5.73618400	2.64629900
C	-4.29889200	8.75128300	-2.68919000
C	-3.55212700	7.43712300	-2.87496300
C	-2.77495300	7.04933100	-1.61756100
C	-2.16577000	5.66221300	-1.75096300
N	-1.34834700	5.35117500	-0.58042200
C	-0.92331200	4.12738200	-0.24093200
N	-1.43770900	3.05159400	-0.85210600
N	0.05208700	3.98782900	0.65984000
O	-1.66188300	0.37593500	-0.14853500
C	-0.91119300	-0.62781300	-0.04417200
O	-1.33974100	-1.82470400	-0.31192000
C	0.51899600	-0.48258500	0.29347500
C	1.45663000	-1.40654100	-0.23498800
C	3.26482100	0.00983200	0.46529500
C	1.00857100	0.61749800	1.03235700
O	8.72152600	-0.81110300	0.29956800
O	4.72580600	3.53212300	1.43615500
O	-3.95774900	2.28121500	1.32689800
H	8.70782400	-4.69219300	-2.29019700
H	8.59115300	-5.92161500	-1.04066600
H	8.03102100	-2.94301000	-0.66108800
H	5.63796800	-3.60933700	-0.52216100
H	6.31820000	-4.07419100	-2.09773900
H	6.07575500	-5.30220600	-0.83562800
H	7.64776000	-5.26722100	1.29492400
H	8.77600400	-3.91335500	1.47225800
H	7.03716600	-3.62623100	1.54467700
H	10.48055400	-4.84463100	0.22190300
H	13.26066500	0.68550300	-1.74430000
H	13.10393600	-0.67366400	-2.82454200
H	11.25910000	1.77804600	-2.83498500
H	11.09363000	0.40674800	-3.90276300
H	13.69936800	2.22153100	-3.72066900
H	8.39818200	2.49739600	4.78892300
H	8.96859600	0.98097100	4.11261300
H	10.08819400	2.21996600	2.24348100
H	9.55904700	3.74730800	2.92782600
H	8.05303500	3.16689300	1.11083500
H	7.16225500	3.06114000	2.62101800
H	8.29184500	0.64340700	1.20450200
H	7.26797700	-1.16973100	1.39689800
H	5.60997100	-1.24009200	2.00038100
H	5.25716900	2.14437700	2.38481800
H	4.71303800	0.61639500	2.98417000
H	10.78715400	3.23758800	5.15872800
H	4.32313300	-6.74031700	-2.33609800
H	3.01193600	-7.73964700	-2.97347600
H	2.18755700	-4.04348300	-1.42694300
H	3.81022700	-4.67146900	-1.54059000
H	4.11116700	-8.89209300	-1.04699700
H	6.73661000	6.25125000	-0.34946000
H	5.73354700	6.12364500	-1.77771600
H	8.87853900	4.16562900	-2.70560400
H	8.48617800	1.65129900	-2.13868200
H	6.35957800	1.38508400	-0.81614200
H	7.75168500	6.54347400	-3.24447100
H	4.59788700	2.83069500	-4.05118300
H	3.92020700	3.17918900	-2.47704800
H	2.63629800	1.35103900	-4.55518800
H	3.44559600	-0.53222900	-3.07441300
H	4.78463500	0.39859000	-3.78173800
H	4.31717800	0.64906000	-2.09616200
H	1.21598300	0.54120800	-2.66351000
H	0.94652200	2.26168100	-2.96075000
H	1.94569900	1.74902900	-1.59529700
H	1.90033900	4.23341900	-3.57439000
H	0.25530300	1.28227500	-5.42555900
H	-1.27492100	0.76900200	-6.11689600
H	2.06250600	-0.71874200	-5.16824000

H	-2.18912700	-1.25953100	-5.32665500
H	2.29735600	-2.85961000	-3.96194200
H	-1.94876600	-3.43889500	-4.15037800
H	0.29992800	-4.22769000	-3.43847000
H	-0.04907800	-0.19895800	-8.10632900
H	-6.42884700	-0.81736600	-5.03806700
H	-7.22206200	-1.58117100	-3.65074700
H	-5.09759400	1.26800200	-2.10948600
H	-2.95780500	0.08635000	-1.15968400
H	-2.67543800	-2.19431000	-2.10200000
H	-7.07614900	1.43908100	-4.13087200
H	-10.62390800	-1.50833100	-0.10544100
H	-10.58081000	-3.18237600	-0.59863000
H	-12.94467500	-2.31262800	-0.67776900
H	-8.53605300	0.44843900	-0.38325500
H	-6.41599600	0.39993700	0.88313000
H	-6.52241500	1.88425500	-0.09027600
H	-6.83302300	1.95903900	1.64990000
H	-8.53552200	-0.68313000	1.80284500
H	-8.94735100	0.83612500	2.62656300
H	-10.11431000	0.08312100	1.53568900
H	-9.16455400	3.02399200	1.16865900
H	-6.67154000	8.95873300	0.71506400
H	-7.28293300	8.94184900	-0.92667200
H	-5.88318000	6.84664300	1.79688200
H	-7.31434100	6.83951600	-2.24578500
H	-4.74601300	4.68279900	1.39552100
H	-6.17079300	4.67734000	-2.64548400
H	-4.33661000	2.99905000	-0.10485300
H	-8.80802700	7.88736100	1.52843500
H	0.83538000	0.95384400	4.06691300
H	-0.10122100	-0.30894800	4.80564100
H	3.60706900	-0.29420700	5.28214500
H	0.31436900	-1.79231600	2.96672100
H	5.09048900	-1.98511000	4.27877600
H	1.80392800	-3.47702700	1.93833800
H	4.20458100	-3.59607400	2.59853600
H	2.21786500	1.21360700	6.15223800
H	1.46989100	3.08352400	4.55903900
H	2.31124100	4.60446100	4.30224900
H	-0.97663500	2.89546800	4.15516300
H	1.08577100	6.61920500	3.56107000
H	-2.95221800	3.79905400	3.00457300
H	-0.91166100	7.54258700	2.42177900
H	-2.94697200	6.13410800	2.13893100
H	0.41547400	4.01749000	6.65681200
H	-2.86307100	7.50772600	-3.72635400
H	-4.26796900	6.64149900	-3.10925800
H	-1.98377500	7.78982300	-1.43129300
H	-3.44453500	7.05872800	-0.75010500
H	-1.55425000	5.58814300	-2.66026100
H	-2.97993500	4.93868000	-1.82952600
H	-0.97935300	6.12477200	-0.04726600
H	-1.25054100	2.11194900	-0.48809700
H	-2.33556000	3.16341700	-1.30424700
H	0.30904800	4.79207400	1.21699500
H	0.15557900	3.07496400	1.11799300
H	-5.01410900	8.67419900	-1.86574500
H	-6.81171700	-5.58210600	3.11317000
H	-8.66075400	-3.15661100	2.68577400
H	-8.56225200	-4.53806400	1.61396700
H	-8.19323400	-2.16790000	-0.73559800
H	-4.97291500	-3.82535500	2.68922100
H	-5.57668500	-1.49082600	-0.74383500
H	-1.76593800	-7.98835200	5.78610800
H	-1.02197300	-5.03544300	6.32547100
H	0.02300300	-6.21781300	5.56915600
H	-2.01473300	-7.39975500	3.48199600
H	-1.48075100	-3.28285500	4.14314800
H	-2.69743300	-6.17023900	1.35257600
H	-3.42920800	-5.41449800	-5.12786800
H	-3.33900600	-7.76679800	-3.13982800
H	-4.67035400	-6.64092000	-3.29900600
H	-0.85176600	-6.31717500	-2.45129700
H	-4.59111200	-4.66906700	-1.33063100
H	-0.44545000	-4.73708000	-0.62449700
H	-4.01458500	-2.10229800	4.55886000
H	-4.38036700	0.79844500	3.61040800
H	-4.77110200	-0.66191600	2.66723400
H	9.67843000	-1.04131000	0.25095600
H	8.67600600	-0.43922300	-0.63944100
H	4.86945100	4.40427700	1.82205400
H	5.18115600	3.57241700	0.53893200
H	-3.18394700	1.82802300	1.73590400

H	-4.73493400	1.78690400	1.61588300
O	-2.09637900	0.83216100	2.69343200
H	-4.68941700	-2.64715600	-3.62374600
H	14.87609800	0.90224000	-3.65374200
H	13.54321100	0.84289400	-4.81542100
H	8.76802300	6.67013900	-1.80090100
H	7.49571800	7.86461000	-2.09442200
H	2.53747400	3.79664700	-5.16494800
H	3.38685800	4.98051700	-4.16365500
H	10.45782600	1.71054400	5.98879800
H	11.36201800	1.71475300	4.46892800
H	10.53233100	-3.52206200	-0.95445900
H	10.96774500	-5.16771800	-1.44183900
H	2.43428600	-8.48362600	-0.63712300
H	3.76961600	-7.51011600	0.00675100
H	-1.36092500	-7.27817000	7.34448400
H	-2.85273600	-6.79505900	6.51851400
H	-6.92073600	-4.18655000	4.19698100
H	-8.30964200	-5.26278300	4.00060700
H	-12.44912900	-1.03825700	-1.81265800
H	-12.40518100	-2.72435300	-2.32051800
H	-8.77839900	2.94637900	-0.55478800
H	-10.26550500	2.19846500	0.05481600
H	-9.42364300	7.87107900	-0.12820300
H	-9.11216600	9.41671400	0.68368600
H	-8.50164600	0.37238000	-4.23366600
H	-7.73977300	0.65461700	-2.67756300
H	1.48878700	0.31731600	-7.40505100
H	0.29801800	1.52884500	-7.90155300
H	-2.06254800	-6.51763900	-4.94959200
H	-3.62021000	-7.13152600	-5.52094200
H	-3.64776000	-0.66225200	5.52881300
H	-5.32029100	-1.01433800	5.05848600
H	0.50397500	1.32799900	6.53562300
H	1.38986600	-0.16501300	6.88663800
H	-3.60969400	9.57192200	-2.46047600
H	-4.85376300	9.02777700	-3.59086400
H	2.18517400	4.06282400	6.74667100
H	1.26998500	5.54289100	6.40178800
O	0.18369900	1.45443700	1.70940000
H	-0.70941200	1.05940300	1.99376200
O	1.04843100	-2.54667600	-0.84644200
H	0.05653400	-2.54026700	-0.80761800
C	2.82419300	-1.14832800	-0.16631800
H	3.52006000	-1.84152100	-0.62380800
C	2.37791700	0.88188500	1.09201600
H	2.72868100	1.77249900	1.60113200
H	4.32532800	0.23009700	0.48404400
Mn	-2.71074200	-2.81200300	1.01469900
